# Influence of Granulocyte-Macrophage Colony-Stimulating Factor or Influenza Vaccination on HLA-DR, Infection and Delirium Days in Immunosuppressed Surgical Patients: Double Blind, Randomised Controlled Trial

**DOI:** 10.1371/journal.pone.0144003

**Published:** 2015-12-07

**Authors:** Claudia Spies, Alawi Luetz, Gunnar Lachmann, Markus Renius, Clarissa von Haefen, Klaus-Dieter Wernecke, Marcus Bahra, Alexander Schiemann, Marco Paupers, Christian Meisel

**Affiliations:** 1 Department of Anesthesiology and Intensive Care Medicine, Campus Charité Mitte and Campus Virchow-Klinikum, Charité – Universitätsmedizin, Berlin, Germany; 2 Sostana GmbH, Berlin, Germany; 3 Department of General, Abdominal and Transplantation Surgery, Charité – Universitätsmedizin Berlin, Campus Virchow-Klinikum, Berlin, Germany; 4 Institute of Medical Immunology, Charité – Universitätsmedizin Berlin, Campus Virchow-Klinikum, Berlin, Germany; University of Pittsburgh, UNITED STATES

## Abstract

**Purpose:**

Surgical patients are at high risk for developing infectious complications and postoperative delirium. Prolonged infections and delirium result in worse outcome. Granulocyte-macrophage colony-stimulating factor (GM-CSF) and influenza vaccination are known to increase HLA-DR on monocytes and improve immune reactivity. This study aimed to investigate whether GM-CSF or vaccination reverses monocyte deactivation. Secondary aims were whether it decreases infection and delirium days after esophageal or pancreatic resection over time.

**Methods:**

In this prospective, randomized, placebo-controlled, double-blind, double dummy trial setting on an interdisciplinary ICU of a university hospital 61 patients with immunosuppression (monocytic HLA-DR [mHLA-DR] <10,000 monoclonal antibodies [mAb] per cell) on the first day after esophageal or pancreatic resection were treated with either GM-CSF (250 μg/m^2^/d), influenza vaccination (Mutagrip 0.5 ml/d) or placebo for a maximum of 3 consecutive days if mHLA-DR remained below 10,000 mAb per cell. HLA-DR on monocytes was measured daily until day 5 after surgery. Infections and delirium were followed up for 9 days after surgery. Primary outcome was HLA-DR on monocytes, and secondary outcomes were duration of infection and delirium.

**Results:**

mHLA-DR was significantly increased compared to placebo (p < 0.001) and influenza vaccination (p < 0.001) on the second postoperative day. Compared with placebo, GM-CSF-treated patients revealed shorter duration of infection (p < 0.001); the duration of delirium was increased after vaccination (p = 0.003).

**Conclusion:**

Treatment with GM-CSF in patients with postoperative immune suppression was safe and effective in restoring monocytic immune competence. Furthermore, therapy with GM-CSF reduced duration of infection in immune compromised patients. However, influenza vaccination increased duration of delirium after major surgery.

**Trial Registration:**

www.controlled-trials.com ISRCTN27114642

## Introduction

Postoperative infections and delirium occur in up to 50% of high-risk patients undergoing high-risk surgery requiring intensive care unit (ICU) and prolonged hospital stay [1–6]. Surgical inflammatory stress can cause delirium and immune suppression [7–10]. Postoperative immune suppression increases the risk for infections and delirium [11–13].

Immune suppression can be measured by a decreased level of human monocyte leukocyte antigen-DR receptor (mHLA-DR) expression on monocytes [12], a hallmark of monocyte deactivation associated with impaired innate and adaptive immune responses [14, 15]. Prolonged downregulation of mHLA-DR has been associated with worse outcomes such as infectious complications, severe sepsis and increased mortality in ICU patients [16–19].

Several studies have demonstrated that suppressed mHLA-DR could be restored by administration of vaccination or Granulocyte-Macrophage Colony-stimulating factor (GM-CSF) [20–27]. Our research group found an increase in mHLA-DR after influenza vaccination in patients with untreated cancer of the upper aero-digestive tract [23] and after preoperative vaccination [28]. Others found the normalization of the immunoregulatory index and the stimulation of the phagocytic function in the absence of essential influence on the level of HLA-DR+ expression [29]. GM-CSF was known to restore reduced mHLA-DR in patients after cardiac surgery *ex vivo* [25] as well as *in vivo* in patients with sepsis [24, 26, 27]. However, whether GM-CSF or vaccination restore HLA-DR after major surgery in immunosuppressed patients is not known.

The primary aim of the study was to investigate the effect of postoperative treatment with GM-CSF or influenza vaccination on mHLA-DR expression. Secondary endpoints were the number of infection and delirium days.

## Materials and Methods

### Study Participants and Design

The first patient was enrolled on October 26, 2008. However, due to technical issues including the payment process, the first acceptance of the corresponding ISRCTN application was delayed (December 05, 2008). As a consequence 4 patients were included in the study before acceptance of the trial registration. The authors confirm that all ongoing and related trials for this drug/intervention are registered.

Patients with immune suppression on day 1 after esophageal or pancreatic resection (*pod1*) were included in this prospective, randomized, placebo-controlled, double-blind, double dummy trial. Immune suppression was defined as reduced mHLA-DR levels of less than 10,000 monoclonal antibodies (mAb) per cell [12]. Patient enrollment was from October 2008 to April 2011. Patients were included if they were scheduled for elective esophageal or pancreatic surgery and had a mHLA-DR expression ≤ 10,000 antibodies per cell on pod1. Exclusion criteria were acquired or congenital blood cell disease, leukemia, autoimmune diseases, allergies to GM-CSF or the influenza vaccine, chemotherapy or radiotherapy within the last 28 days, proven infection within the last 7 days, infection with HIV or hepatitis B or C, pharmacological immune suppression within the last 4 weeks, unstable angina pectoris, untreated arrhythmia, thromboembolic events, cachexia, thrombocytopenia (≤ 100,000 /μl), neutropenia (≤ 1,500 /μl), anemia (hemoglobin ≤ 8 g/dl), hyperbilirubinemia (> 2 g/dl), creatinine > 1.5 g/dl, aspartate aminotransferase (AST) or alanine aminotransferase (ALT) above 90 U/I, pregnancy or lactation, or participation in a German Drug Law (AMG) one month before and during the study.

**Fig 1:**
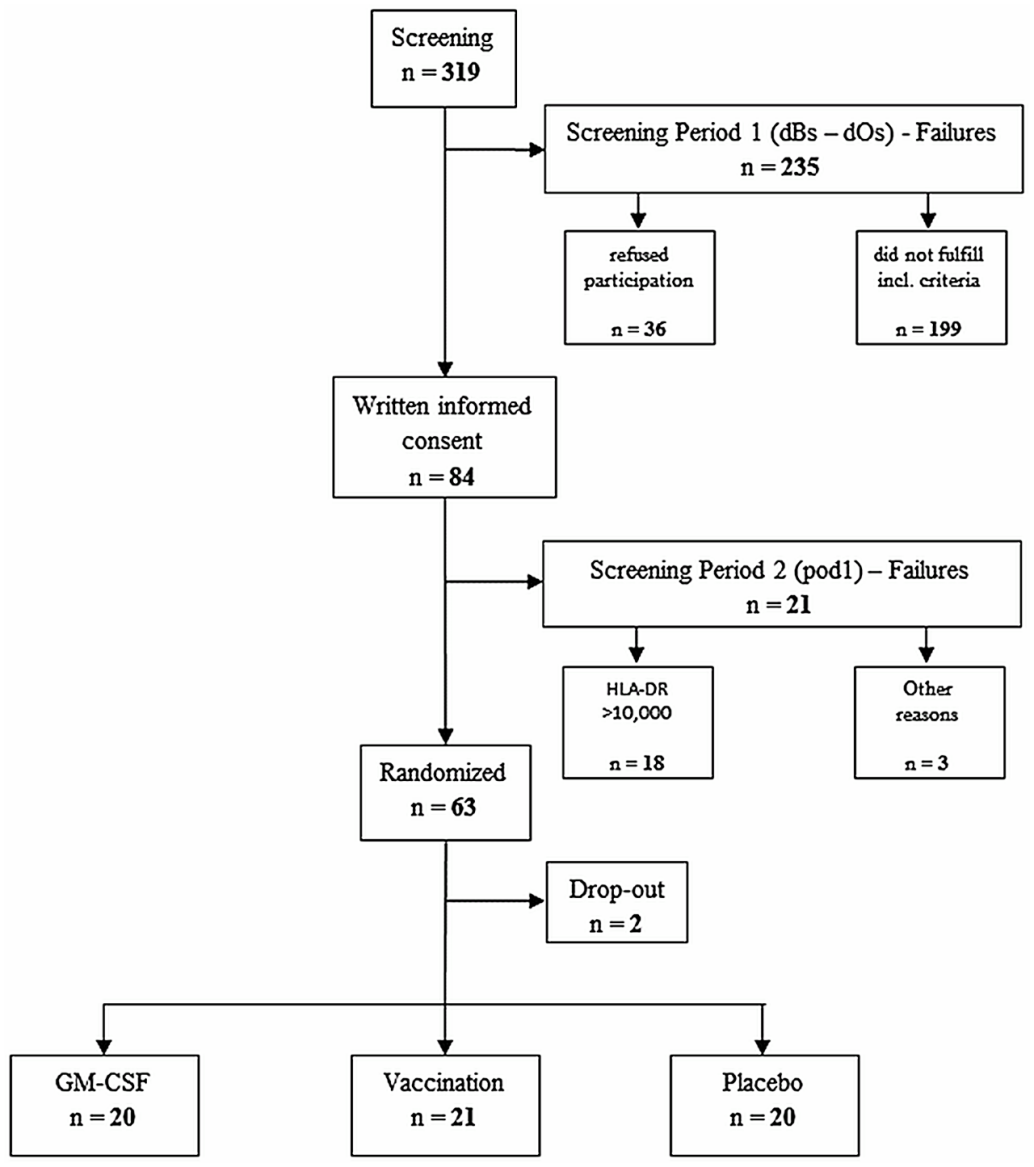
Consort diagram. *dBs*, day before surgery. *dOs*, day of surgery. *pod*, postoperative day.

A total of 319 patients scheduled for esophageal or pancreatic surgery were screened. Of these, 235 did not fulfil inclusion criteria or refused to participate and 84 patients gave their written informed consent to participate. Of the 84 patients who consented, 21 patients had to be excluded for the following reasons: 18 patients were not immune suppressed after surgery, 1 patient’s operation was cancelled, in 1 patient pancreatic resection was not performed and 1 patient had abnormal preoperative laboratory values which were not available preoperatively and therefore delayed after surgery. The remaining 63 patients were immune suppressed and were randomized according to the study protocol. Two of the patients were excluded (major protocol violations) after randomization because one patient was inadvertently unblinded (placebo group) and one patient died of multiorgan failure after receiving study medication for 12.5 h (GM-CSF group, no causal relation to the study drug). Three additional patients were randomized due to these two dropouts of the initially planned 60 patients. Finally, 61 patients were included in data analysis (Fig 1). There were 23 minor protocol violations of inclusion criteria and 1 minor protocol violation regarding termination of the study medication.

The study was approved by the Ethics Committee of the Landesamt für Gesundheit und Soziales Berlin (LaGeSo), Germany (ref ZSEK15287/08) on September 01, 2008. This clinical trial meets the requirements set out by the ICH-GCP, Declaration of Helsinki and the German Drug Law (AMG). Written informed consent was obtained from patients.

### Randomization and Treatment with Study Drugs

For blinding, consecutively numbered (randomization numbers) closed envelopes were used, which contained the assignment to the therapy. In case of adverse reactions, severe deterioration of patient’s state or other unforeseen events the patient and/or the physician had to be unblinded. A list with the randomization numbers and instructions for treatment was available only to the study statistician and the local pharmacy. The medications were prepared by an independent medically qualified person, who covered all medications for blinding. This person was otherwise not involved in this study.

GM-CSF was administered intravenously (i.v.) and vaccination subcutaneously (s.c.). To avoid bias, we applied a “double dummy” design: after inclusion patients were randomized in permutated blocks of six to receive either GM-CSF i.v. and 0.9% sodium chloride (NaCl) s.c., vaccination with the haemagglutinin antigens of the influenza virus s.c. and NaCl i.v. or placebo (NaCl i.v. and NaCl s.c.). Starting on *pod1*, study drugs were administered as follows:

*- GM-CSF group*: 1 s.c. syringe with 0.5 ml of 0.9% NaCl and 1 perfusor syringe with 24 ml of **sargramostim** (Leukine, Bayer Health Care, LLC. Seattle, WA 98101, USA) **250 μg/m** ^**2**^ **body surface** in 0.9% NaCl (1 ml/h i.v.)
*- Vaccine group*: 1 s.c. syringe with **0.5 ml of influenza vaccine** (Mutagrip 2009/2010, Sanofi Pasteur MSD GmbH, Leimen, Germany), 1 perfusor syringe with 24 ml of 0.9% NaCl (1 ml/h i.v.)
*- Placebo group*: 1 s.c. syringe with 0.5 ml of 0.9% NaCl, 1 perfusor syringe with 24 ml of 0.9% NaCl (1 ml/h i.v.)

**Fig 2:**
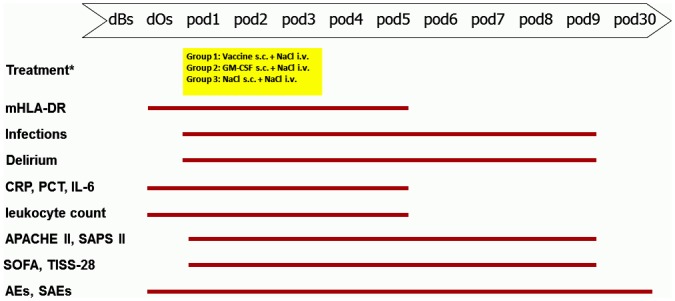
Flow diagram. *dBs*, day before surgery. *dOs*, day of surgery. *pod*, postoperative day. *Study drug treatment for a maximum of 3 consecutive days (*pod1* –*pod3*) depending on mHLA-DR expression.

All patients were treated with the study medication for 24 hours. If mHLA-DR levels remained below 10,000 mAb per cell on day 2 and day 3 (*pod2* and *pod3*) after surgery, administration of the study drugs was continued for a maximum of 72 hours (Fig 2).

Physicians in charge were unaware of group assignments and provided treatment without interference by the researchers. All patients received standard perioperative treatment according to our standard operating procedures [30].

### Flow Cytometry and Measurement of Soluble Mediators

Blood samples were drawn daily starting on the day of surgery (dOs) until day 5 after surgery (*pod5*) for measurement of mHLA-DR expression, leukocytes, C-reactive protein (CRP), procalcitonin (PCT) and interleukin 6 (IL-6).

For detailed information see (S1 Methods).

### Clinical Outcome Parameters

During the follow-up period until day 9 after surgery (*pod9)*, we measured infections according to CDC and ATS for pneumonia [31, 32], incidence of delirium using the Delirium Detection Score (DDS, [6]) with a DDS > 3, hospital and ICU stay and disease severity using the following scores: Acute Physiology and Chronic Health Evaluation II (APACHE-II), Sequential Organ Failure Assessment (SOFA), Simplified Acute Physiology Score-II (SAPS-II) and Therapeutic Intervention Scoring System (TISS)-28. All adverse and serious adverse events that occurred within 9 days after surgery were followed up until day 30 after surgery (Fig 2).

### Statistical Analysis

Data were summarized using arithmetic mean ± standard deviation (SD), median [25%, 75% quartiles], or frequency [%], as appropriate. Since this was an exploratory pilot study, sample size calculations were not performed. Due to the distribution of the data, tests were performed using non-parametric methods.

For the primary endpoint mHLA-DR, infection duration, and delirium duration, differences among treatment groups on the first and second postoperative days (pod1 and pod2) were assessed using the Kruskal-Wallis test followed by pairwise comparisons using the Mann-Whitney test. Differences within treatment groups between pod1 and pod2 (pre and post treatment) were assessed using the paired Wilcoxon test. Basic patient characteristics and post intervention characteristics were evaluated for group differences using the Kruskal-Wallis test for continuous variates and the Fisher exact test for categorical variates.

All tests were two-tailed. Statistical significance was declared at the 0.05 level. Since this is an exploratory analysis, no adjustments for multiple testing were carried out. All calculations were performed with IBM SPSS Statistics, Version 19, and SAS (Version 9.1) software.

## Results

### Study Population and Study Groups

Basic patient characteristics did not differ between groups except for gender (p = 0.045) (Table 1). In the GM-CSF group, study medication was given to all patients on *pod1*, to 1 patient on *pod2*, and to 3 patients on *pod3*. In the vaccination group, study medication was given to all patients on *pod1*, to 20 patients on *pod2* and to 15 patients on *pod3*. In the placebo group, study medication was administered in all patients on *pod1* and *pod2* as well as in 17 patients on *pod3*.

**Table 1:** Basic patient characteristics and pre-interventional course. Continuous quantities in median (25%-75% percentiles), frequencies with n (%); NRS, Numeric Rating Scale; *dBs*, day before surgery; *pod1*, day 1 after surgery; ASA, American Society of Anesthesiologists; AUDIT score, Alcohol Use Disorders Identification Test; PONV, postoperative nausea and vomiting; APACHE, Acute Physiology and Chronic Health Evaluation; SAPS, Simplified Acute Physiology Score; SOFA, Sequential Organ Failure Assessment; TISS, Therapeutic Intervention Scoring System; ICU, Intensive Care Unit.

	Placebo (n = 20)	Vaccine (n = 21)	GM-CSF(n = 20)	p-value (all groups)
Age [years]	64 (55–74)	67 (62–72)	64 (53–70)	*0*.*251*
Gender male/female [n (%)]	11/9 (55/45)	15/6 (71/29)	18/2 (90/10)	*0*.*045*
Body Mass Index [kg/m^2^]	25.5 (23.6–28.3)	24.5 (21.2–29.3)	26.9 (23.2–28.1)	*0*.*917*
Pancreatic/esophageal resection [n]	9/11	13/8	10/10	*0*.*588*
ASA score II/III [n]	12/8	9/12	9/11	*0*.*532*
smoker/nonsmoker/did never smoke [n]	8/5/7	6/10/5	5/9/6	*0*.*601*
AUDIT score	3 (0–6)	1 (0–7)	3 (0–5)	*0*.*989*
Metabolic equivalent (MET) <4/4-10/>10	1/17/2	2/18/1	0/19/1	*0*.*853*
NRS at rest *dBs*	0 (0–3)	0 (0–0)	0 (0–2)	*0*.*356*
NRS at rest *pod1*	3 (1–3)	3 (0–5)	3 (0–5)	*0*.*525*
NRS during movement *pod1*	5 (2–8)	4.5 (3–8)	6 (3–9)	*0*.*351*
Surgical time [min]	360 (304–440)	345 (275–430)	360 (345–430)	*0*.*508*
Blood loss [ml]	625 (338–1000)	600 (400–800)	700 (500–1100)	*0*.*616*
Blood glucose [mg/dl]	131 (120–139)	140 (128–164)	137 (122–147)	*0*.*150*
Blood lactate [mmol/l] (max.)	1.44 (1.10–1.87)	1.67 (1.06–1.95)	1.72 (1.24–2.64)	*0*.*549*
Systolic blood pressure [mmHg]	115 (112–120)	122 (110–130)	115 (112–125)	*0*.*446*
PONV	2 (1.00–3.00)	1 (0.00–2.00)	1 (1.00–2.00)	*0*.*510*
APACHE II scoreon admission to ICU	16 (11–18)	14 (7–18)	12 (11–16)	*0*.*411*
SAPS II scoreon admission to ICU	27 (21–42)	25 (17–30)	28 (22–33)	*0*.*829*
SOFA scoreon admission to ICU	2.5 (0.3–5.0)	0.0 (0.0–3.0)	1.0 (0.0–4.8)	*0*.*185*
TISS 28 score on admission to ICU	32.5 (28.0–38.5)	32.0 (25.0–37.0)	32.0 (29.0–35.5)	*0*.*876*

### Primary endpoint (mHLA-DR)

**Fig 3:**
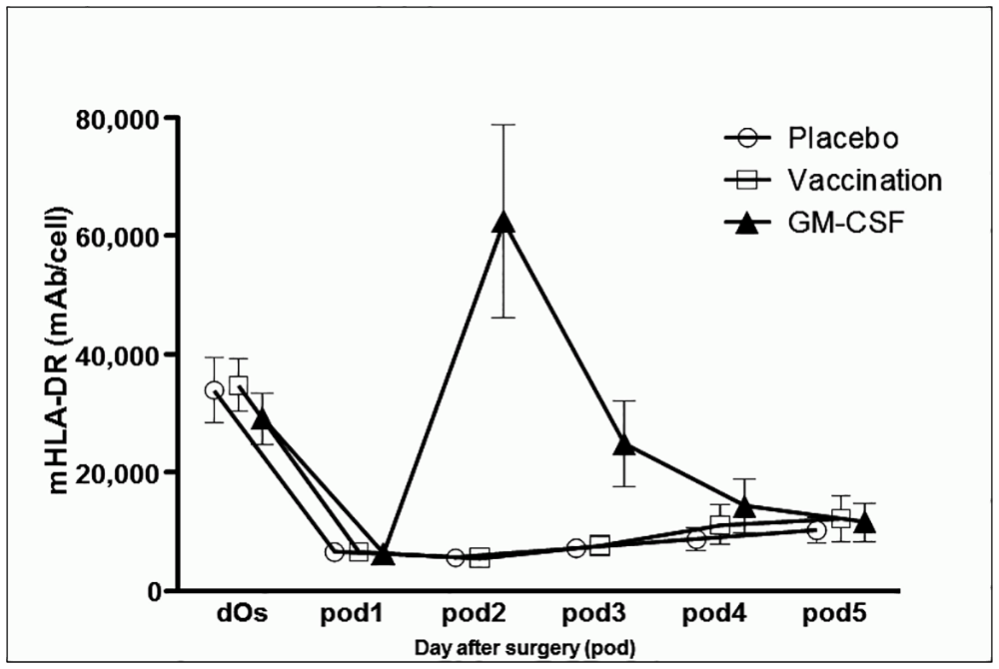
Monocytic HLA-DR (mHLA-DR) over time. Immediately before surgery (*dOs*) until day 5 after surgery (*pod5*) between groups.

HLA-DR was increased compared to placebo (p < 0.001) and influenza vaccination (p < 0.001) on *pod2* (Fig 3 and Table 2). Differences between groups with respect to pre-post (*pod1 –pod2*) were seen for patients who received GM-CSF compared to those who received vaccination (p < 0.001) and placebo (p < 0.001; Table 2).

**Table 2:** Monocytic HLA-DR (mHLA-DR) on day 1 (*pod1)* and day 2 *(pod2)* after surgery. Paired Wilcoxon Test (***pod1 versus pod2***): Placebo: p = 0.0027; Vaccination: p = 0.013; GM-CSF: p < 0.001; Overall (Kruskal-Wallis): ***pod1***: p = 0.667; ***pod2***: p < 0.001; ***pod1 –pod2***: p < 0.001; IQR: Interquartile Range.

	Mean	Standard deviation	Median	IQR	p-values (Mann-Whitney)		
***pod1***					1 vs. 2	1 vs. 3	2 vs. 3
1: Placebo	6,688	1,805	6,709	2,707	0.906	0.426	
2: Vaccination	6,740	1,614	6,777	2,287			0.467
3: GM-CSF	6,306	2,272	6,080	4,062			
***pod2***					1 vs. 2	1 vs. 3	2 vs. 3
1: Placebo	5,712	1,853	5,668	2,759	0.633	< 0.001	
2: Vaccination	5,643	2,294	5,448	3,372			< 0.001
3: GM-CSF	62,566	34,975	61,257	34,021			
***pod1 –pod2***					1 vs. 2	1 vs. 3	2 vs. 3
1: Placebo	976	1,730	1,146	3,005	0.876	< 0.001	
2: Vaccination	1,097	1,748	1,278	2,828			< 0.001
3: GM-CSF	-56,260	33,910	-54,649	33,541			

### Secondary endpoints (infection and delirium, infection days and delirium days)

**Fig 4:**
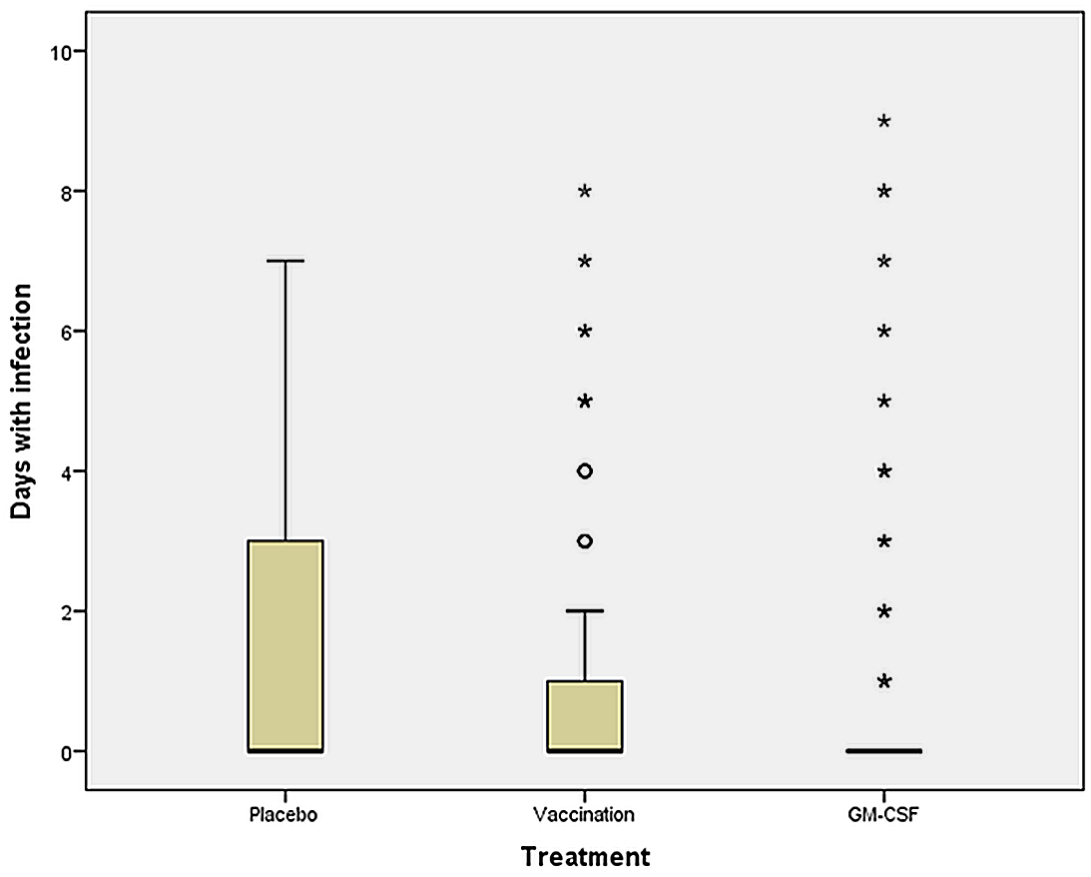
Number of infection days. From day 1 (*pod1*, before intervention) until day 9 after surgery (*pod9*). Between all three groups: p = 0.001. Placebo vs. GM-CSF: p < 0.001. Placebo vs. vaccination: p = 0.068. Vaccination vs. GM-CSF: p = 0.044.

Fewer infection days were seen in the GM-CSF-treated patients (p < 0.001) after surgery (Fig 4; Table 3) compared to placebo.

**Table 3:** Infection and delirium days (DDS > 3). Overall (Kruskal-Wallis): **Infection days**: p = 0.001; **Delirium days (DDS > 3)**: p = 0.004; IQR: Interquartile Range.

	Mean	Standard deviation	Median	IQR	p-values (Mann-Whitney)		
**Duration of Infection (days)**					1 vs. 2	1 vs. 3	2 vs. 3
1: Placebo	1.35	1.982	0.00	3	0.068	< 0.001	
2: Vaccination	1.05	1.883	0.00	2			0.044
3: GM-CSF	0.74	1.807	0.00	0			
**Duration of Delirium (days)**					1 vs. 2	1 vs. 3	2 vs. 3
1: Placebo	0.6222	1.1194	0.00	1	0.003	0.737	
2: Vaccination	0.9788	1.3247	0.00	2			0.007
3: GM-CSF	0.5556	0.8471	0.00	1			

During *pod1* to *pod9*, infection was seen in 11 patients in the placebo group, in 9 patients in the vaccine group and in 8 patients in the GM-CSF group. Pneumonia was the most frequent type of infection (n = 18) followed by sepsis (n = 6), wound infection (n = 5), urinary tract infection (n = 3) and other infections (n = 2). These results did not differ between groups.

**Fig 5:**
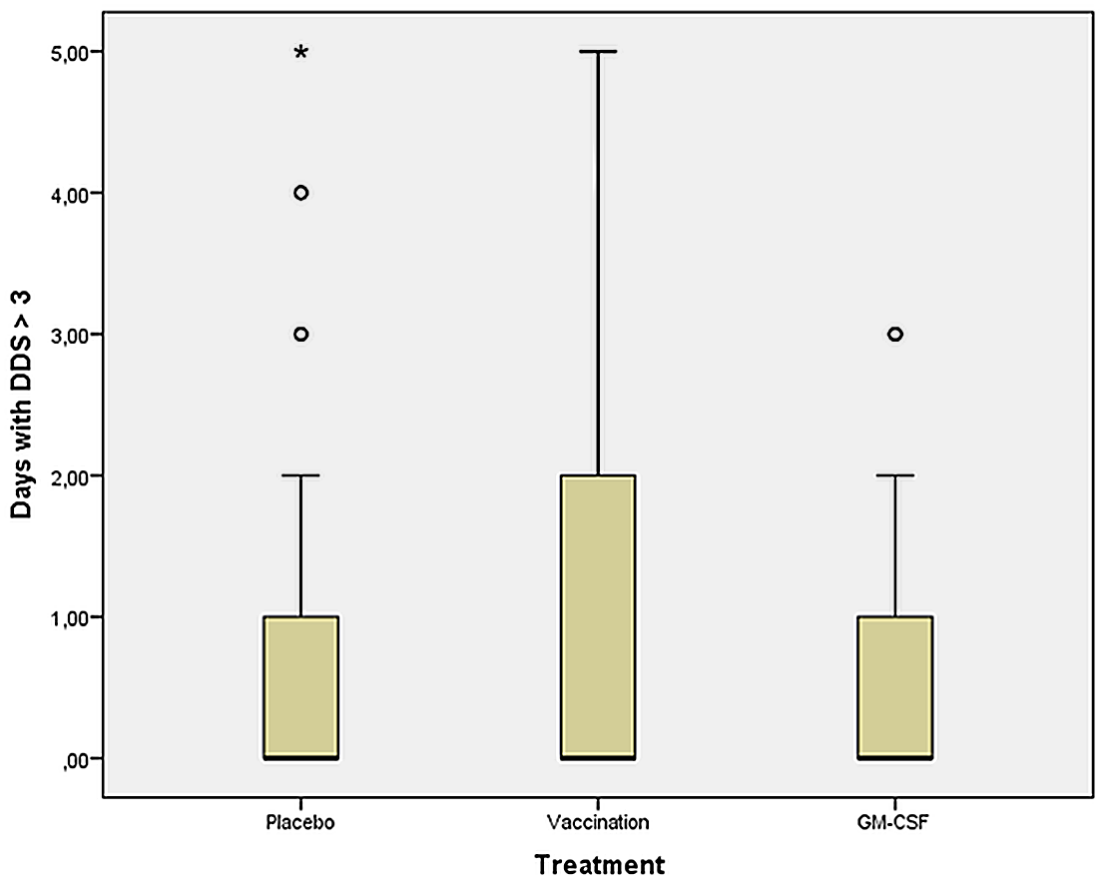
Number of delirium days (DDS > 3). From day 1 (*pod1*, before intervention) until day 9 after surgery (*pod9*). Between all three groups: p = 0.004. Placebo vs. GM-CSF: p = 0.737. Placebo vs. vaccination: p = 0.003. Vaccination vs. GM-CSF: p = 0.007.

Delirium days were increased after influenza vaccination compared to the other groups (p = 0.003 and p = 0.007) after surgery (Fig 5; Table 3).

For the period of *pod1* to *pod9*, delirium rate did not differ significantly between groups (11 patients in the placebo group, 14 patients in the vaccine group and 12 patients in the GM-CSF group).

**Fig 6:**
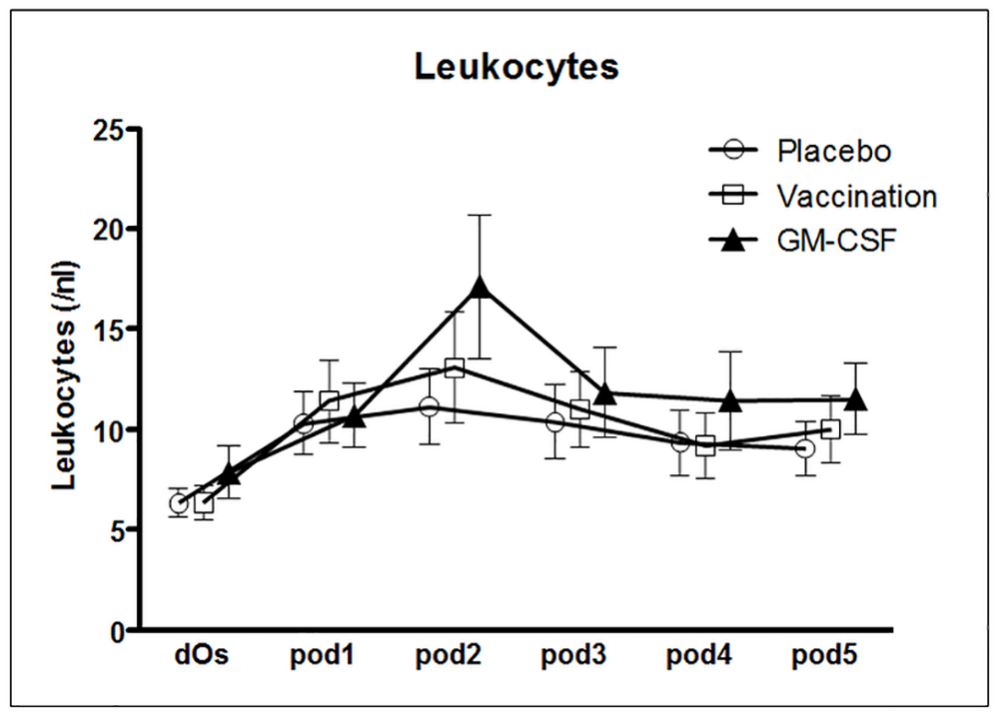
Leukocytes over time. From day of surgery (dOs) until day 5 after surgery (*pod5*) between groups. Leukocytes significantly increased on pod2 (p = 0.003) after stimulation with GM-CSF.

Leukocytes increased on *pod2* after stimulation with GM-CSF and differed significantly from placebo (p = 0.003; Fig 6). Temperature, CRP, PCT and IL-6 did not show any significant differences between the groups.

There were no significant differences in intra- and other postoperative parameters or in postoperative pain, postoperative nausea and vomiting or in ICU scores on admission (Table 1). In addition, maximum of SOFA and TISS 28 score as well as time on ventilator, ICU and hospital length of stay did not significantly differ between the groups (Table 4).

**Table 4:** Post-interventional course and outcome parameters. Continuous quantities in median (25%-75% percentiles), frequencies with n; APACHE, Acute Physiology and Chronic Health Evaluation; SAPS, Simplified Acute Physiology Score; SOFA, Sequential Organ Failure Assessment; TISS, Therapeutic Intervention Scoring System; ICU, Intensive Care Unit.

	Placebo (n = 20)	Vaccine (n = 21)	GM-CSF (n = 20)	p-value (all groups)
SOFA score (max.)	3.5 (0.3–6.0)	2.0 (0.0–4.5)	4.0 (1.0–7.0)	*0*.*414*
TISS 28 score (max.)	34.5 (31.0–40.0)	33.0 (25.0–40.0)	36.0 (31.0–39.5)	*0*.*788*
ICU stay (d)	3.9 (2.3–4.9)	4.4 (0.8–21.3)	1.9 (0.8–5.0)	*0*.*327*
Hospital stay (d)	15.4 (12.3–22.2)	15.0 (13.0–31.5)	15.2 (11.2–25.3)	*0*.*553*

No study drug-related serious adverse events, adverse drug-related reactions, unexpected adverse reactions or suspected unexpected severe adverse reactions were observed. A detailed documentation of the safety data is given in (S1 Data).

## Discussion

The major finding of this study was that GM-CSF treatment after surgery in immune compromised patients increased mHLA-DR expression and decreased the number of infection days whereas influenza vaccination increased the number of delirium days after major surgery. To the best of our knowledge, there have been no studies that investigated postoperative *in vivo* biomarker-guided administration of GM-CSF or vaccination in immune compromised surgical patients.

Several previous studies have demonstrated that GM-CSF can improve immune responses in immunosuppressed patients both *ex vivo* and *in vivo*. Börgermann et al. showed that decreased mHLA-DR expression could be restored by GM-CSF *ex vivo* in patients undergoing cardiopulmonary bypass [25]. The same group demonstrated that GM-CSF also increases *ex vivo* endotoxin-induced monocyte cytokine release in immunosuppressed trauma patients [33]. In recent open-label and randomized trials, we and others have shown that GM-CSF treatment can restore innate immune responses in adult patients with severe sepsis-associated immunosuppression and in children with multiple organ dysfunction syndrome [24, 26, 34]. In accordance with previous studies, we observed an immediate increase of mHLA-DR to pre-operative or even supernormal levels in patients with surgery-induced immunosuppression on the 1^st^ day after administration of GM-CSF. This increase most likely relies on an induction of key molecules in the class II MHC pathway of monocytes [35]. However, mHLA-DR levels rapidly decreased when GM-CSF treatment was discontinued which is likely due to the short half-life of GM-CSF and replacement of circulating monocytes from the bone marrow pool. Noteworthy, on the fifth day after surgery it decreased to levels comparable to the placebo group (mHLA-DR was over 10,000 mAb/cell but still depressed compared to preoperative levels). GM-CSF treatment also increased the postoperative leukocyte counts while other markers of inflammation and infection such as temperature, CRP, PCT and IL-6 remained unaffected. These data are in accordance with previous studies, which showed no effect of GM-CSF treatment on the infection parameters PCT and IL-6 in critically ill patients [24, 26, 34].

Recent studies suggested that influenza vaccination could also increase suppressed mHLA-DR expression. Haining et al. demonstrated increased mHLA-DR expression after influenza vaccination in children after hematopoietic stem cell transplantation [20]. We showed similar effects in patients with upper aerodigestive tract cancer [23]. In contrast, in the same study influenza vaccination failed to significantly prevent downregulation of mHLA-DR expression when the vaccine was administered pre-operatively [23]. Thus, in conjunction with the current study demonstrating no beneficial effect of post-operative influenza vaccination in immunosuppressed surgical patients, vaccination at least with influenza vaccine did not appear to be an effective immunostimulatory treatment approach either to prevent or to reverse surgery-induced immunosuppression. After influenza vaccination, healthy volunteers showed a significant increase in the frequency of H1N1-specific IL-4+CD4+ T cells after vaccination. However, healthy volunteers also showed an increase in IFN-γ+CD4+ T cells after vaccination despite the fact that no significant changes in HLA-DR- and CD86 expression from before to after vaccination were observed. In addition, the cytokine profile after administration of vaccine showed that healthy controls had significantly greater levels of fractalkine, IFN-γ, MCP-3, interleukin 1β, interleukin 6, and MIP-1α [36] i.e. they had a higher inflammatory cytokine load that might have influence on microglial activation and delirium days [37].

A recent meta-analysis of 12 randomized controlled trials including 2,380 patients by Bo et al. analyzed the effect of GM-CSF on clinical outcome parameters in sepsis. GM-CSF was demonstrated to significantly increase the reversal rate of infections in sepsis patients [38]. In addition, Hall et al. found a significant reduction in secondary infectious complications in critically ill children under GM-CSF treatment [34]. However, until now no study has shown a decreased rate of nosocomial infections in surgical patients at risk after treatment with GM-CSF. In our study, the infection days after surgery were significantly lower under GM-CSF treatment compared to placebo and vaccination group. Nevertheless, despite the fact that the infection days were decreased up to day 9 after surgery, the total number of infections did not differ between groups. These data suggest that GM-CSF treatment may mitigate the severity and duration but may not prevent the development of post-operative infectious episodes. However, given that GM-CSF best protected patients from infections up to day 5 after surgery, short-term immunostimulatory treatment may not be sufficient. In addition, mortality and comorbidity endpoints other than infection were not influenced by GM-CSF. However, the current study was not powered to assess these endpoints. Thus, additional studies are needed to determine the most effective duration of immunostimulatory treatment regimens and appropriate biomarker signatures indicative for sustained recovery of immune function and improved clinical outcome.

Another finding of our study is that vaccination increased the number of delirium days. The number of delirium days is positively associated with increased 1-year mortality [39, 40]. To the best of our knowledge, there is currently no study that describes a risk for delirium after influenza vaccination at all [41–43] aside from one case report [44]. However, the pathogenesis of delirium is still not clearly understood. Some studies suggest that systemic inflammation may affect cholinergic and immune system are of major relevance in the development of delirium [45–47]. Hingorani et al. [48] and Clapp et al. [49] showed that vaccination leads to a systemic inflammatory response which is associated with endothelial dysfunction. However, they used salmonella typhi vaccination. Thus, we hypothesize that in our study influenza vaccination most likely induced a systemic inflammation which increased the risk of delirium perhaps due to disruption of the blood-brain barrier [37, 50, 51]. Influenza vaccination may have negative effects on delirium that might be related to unspecific inflammatory stimulation and should be investigated in further studies.

This study has several limitations. First, study drugs were administered only for a maximum of 3 days. The effect of longer duration of therapy is unclear. Second, we observed small differences in univariate analyses for gender between the groups, but this was only descriptive because multivariate analysis is not considered appropriate in that setting and would not show any difference. Third, the threshold level for mHLA-DR (≤ 10,000 mAb/cell) used to select patients with severe surgery-induced immunosuppression for immunostimulatory therapy in this study is unclear. Previous studies have suggested using a mHLA-DR threshold between 5,000 and 10,000 mAb/cell as an indicator of severely impaired immune function in critical ill patients [12, 24, 52]. However, it is currently unclear whether single cut-off values for mHLA-DR can be applied to identify best patients at risk with different entities of trauma-associated immunosuppression. It was recently demonstrated that mHLA-DR levels of < 20,000 mAb/cell were associated with the development of pneumonia in patients with severe stroke [53]. Thus, further studies are needed to determine outcome-relevant thresholds for biomarkers of immune competence in order to optimize the risk-benefit profile of immunostimulatory therapeutic approaches to overcome severe immunosuppression in critical illness. Fourth, the use of influenza vaccine might have been insufficient to treat the HLA-DR response despite the fact that proinflammatory stimulation might have increased delirium. A potential improvement would be to use a vaccine known to increase HLA-DR response such as vaccination against hepatitis B [54]. Finally, we observed patients for 9 days after surgery for infections. Infections occurring after this time were not prospectively documented and could not be evaluated. Furthermore, in this study we included patients after pancreatic and esophageal resection.

## Conclusions

Postoperative application of GM-CSF significantly increased HLA-DR expression on monocytes and decreased the duration of infection after surgery in patients undergoing esophageal or pancreatic surgery. Postoperative influenza vaccination had no influence on post-surgical immunosuppression but increased the number of delirium days. Further studies are warranted to evaluate the benefit of immunostimulatory therapy with GM-CSF to improve clinical outcome. The negative effects of influenza vaccination on cognition should be investigated as well.

## Supporting Information

S1 Methods Methods of flow cytometry and measurement of soluble mediators. (DOC)

S1 Data Safety data: Adverse events (AE) and Serious adverse events (SAE). (DOC)

S1 Protocol Study protocol (German). (PDF)

S2 Protocol Translation of study protocol (synopsis). (DOC)

S1 Checklist CONSORT Checklist. (DOC)

S1 Diagram CONSORT Flow Diagram. (DOC)
