# Supplementary material for: Influence of Granulocyte-Macrophage Colony-Stimulating Factor or Influenza Vaccination on HLA-DR, Infection and Delirium Days in Immunosuppressed Surgical Patients: Double Blind, Randomised Controlled Trial
Source: PLoS One. 2015 Dec 7;10(12):e0144003. doi: 10.1371/journal.pone.0144003 (PMC4671639; doi:10.1371/journal.pone.0144003)
Supplement: S1 Methods — (DOC) [file pone.0144003.s001.doc]

**Methods of flow cytometry and measurement of soluble mediators**

All parameters were analyzed in cooperation with the Institute of Medical Immunology and Berlin-Brandenburg Center for Regenerative Therapies (BCRT), Charité – Universitätsmedizin Berlin, Berlin, Germany.

Expression of HLA-DR on monocytes was determined by flow cytometry. Briefly, 50 µl of EDTA-blood was stained with 20 µl of monoclonal phycoerythrin-conjugated anti-human leukocyte antigen-DR (HLA-DR) antibodies and peridin chlorophyll (PerCP-Cy5.5)-conjugated anti-CD14-antibodies (BD Quantibrite™ HLA-DR / Monocyte reagent, BD Biosciences) for 30 min in the dark at room temperature. For lysis of erythrocytes, samples were incubated with 500 µl BD FACS Lysing solution (BD Biosciences) for 15 min in the dark at room temperature. Subsequently, cells were washed with 1 ml of FACS buffer and analyzed on a FACS Calibur flow cytometer using CellQuest™ software after QuantiBrite calibration for 1:1 quantification. Final analysis was performed using Quanticalc™ software (all from BD Biosciences) to obtain the molecules HLA-DR per cell from the measured geometric means.

White blood counts were performed on a standard hematology analyzer (Sysmex), plasma CRP levels were determined by an immunotur-bidometric assay (Roche Diagnostics). After centrifugation of whole blood IL-6 was deter-mined in the supernatant with the IMMULITE Automated Analyzer (DPC Biermann, Bad Nauheim, Germany), Procalcitonin concentration was measured using a commercial immunoluminometric assay (Brahms, Hennigsdorf, Germany).
