# Supplementary material for: Influence of Granulocyte-Macrophage Colony-Stimulating Factor or Influenza Vaccination on HLA-DR, Infection and Delirium Days in Immunosuppressed Surgical Patients: Double Blind, Randomised Controlled Trial
Source: PLoS One. 2015 Dec 7;10(12):e0144003. doi: 10.1371/journal.pone.0144003 (PMC4671639; doi:10.1371/journal.pone.0144003)
Supplement: S1 Data — (DOC) [file pone.0144003.s002.doc]

**Safety data: Adverse events (AE) and Serious adverse events (SAE)**

During the present trial in 63 enrolled patients (including 2 drop-outs) 335 adverse events (AE) and 70 severe adverse events (SAE) were seen. No study drug-related serious adverse events or adverse drug-related reactions, no unexpected adverse reactions and no suspected unexpected severe adverse reactions (SUSAR) were seen. There were 111 AEs (33%) in all vaccination patients, 111 AEs (33%) in all placebo patients and 113 AEs (34%) in all GM-CSF patients. There were 32 SAEs (46%) in 12 vaccination patients who received vaccination, 20 SAEs (28%) in 10 study placebo patients and 18 SAEs (26%) in 10 study GM-CSF patients. One patient who received GM-CSF died during the study because of a bowel ischemia in hospital (no study drug-related SAE). 4 patients died because of septic multiorgan failure, one patient 33 days after administration of GM-CSF, one patient due to sepsis and peritonitis 30 days after vaccination, one patient due to septic shock 34 days after vaccination and one patient due to cardiac arrest 135 days after vaccination.
