# Supplementary material for: Influence of Granulocyte-Macrophage Colony-Stimulating Factor or Influenza Vaccination on HLA-DR, Infection and Delirium Days in Immunosuppressed Surgical Patients: Double Blind, Randomised Controlled Trial
Source: PLoS One. 2015 Dec 7;10(12):e0144003. doi: 10.1371/journal.pone.0144003 (PMC4671639; doi:10.1371/journal.pone.0144003)
Supplement: S1 Protocol — (PDF) [file pone.0144003.s003.pdf]

ChariteCentrum für Anästhesiologie, OP-Management und Intensivmedizin

Charité - Universitätsmedizin Berlin | 13344 Berlin

Universitätsklinik für Anästhesiologie
mit Schwerpunkt operative Intensivmedizin
CCM / CVK

Klinikdirektorin:
Univ.-Prof. Dr. C. Spies

Zertifiziert nach DIN EN ISO 9001

Campus Virchow-Klinikum
Augustenburger Platz 1
13353 Berlin
Tel: +49 30 450 551-001/002/022
Fax: +49 30 450 551909
anaesthesie-virchow-klinikum@charite.de

Campus Charité Mitte
Chariteplatz 1
10117 Berlin
Tel: +49 30 450 531-012/52
Fax: +49 30 450 531911
anaesth@charite.de

http://anaesthesieintensivmedizin.charite.de/

PRÜFPLAN

EudraCT-Nr.: 2007-003111-31

Kurztitel: ART VI

Studientitel:

„Einfluss einer postoperativen Vakzination oder von GM-CSF bei immunsupprimierten Patienten nach Pankreas- oder Ösophagusresektion auf den Verlauf einer Immunsuppression und die postoperative Infektionsrate.“

- Eine randomisierte, dreiarmige, placebokontrollierte, doppelblinde Pilotstudie im Double-Dummy-Design -

Version 2.1

Datum: 31.08.2010

Sponsor der klinischen Prüfung:

Charité – Universitätsmedizin Berlin, Charitéplatz 1, 10117 Berlin

Vertreter des Sponsors und Hauptprüfer der klinischen Prüfung:

Prof. Dr. med. Claudia Spies

Die nachfolgenden Personen stimmen den Inhalten dieses Protokolls durch ihre Unterschrift zu und bestätigen, dass ihnen die ICH-GCP-Guidelines, die Anforderungen des AMG und der GCP-Verordnung bekannt sind und dass die klinische Prüfung nach diesen Vorschriften durchgeführt wird.

**Vertreter des Sponsors, medizinischer Experte und Hauptprüfer der klinischen Prüfung**	[signature]<br>**Prof. Dr. Claudia Spies**	Berlin 15.09.10<br>**Ort, Datum**

- Vertraulich -

Die Informationen in diesem Protokoll sind streng vertraulich zu behandeln. Sie dienen nur zur Information des Prüfarztes, seiner Mitarbeiter, der Ethikkommission, der Bundesoberbehörde (PEI), der CRO, der KKS und zur Patientenaufklärung.

Inhaltsverzeichnis

Synopsis 10
1 Ablaufschema 22
2 Einleitung 23
2.1 Einleitung und Hintergrund zur klinischen Prüfung 23
2.2 Stand des Wissens über die Prüfpräparate 25
2.2.1 Influenza-Impfstoff: MUTAGRIP® 2007/2008 (Sanofi Pasteur MSD GmbH) 25
2.2.2 Sargramostim (=GM-CSF, Leukine®, Bayer Health Care™) 27
2.2.3 Isotonische Kochsalzlösung (Braun) 34
2.3 Begründung der Behandlungs- und Untersuchungsverfahren 34
3 Ziele der klinischen Prüfung 35
3.1 Primärer Endpunkt 35
3.2 Sekundäre Endpunkte 36
3.3 Studiendesign 36
3.4 Zeitplan 37
4 Auswahl der Patienten 38
4.1 Einschlusskriterien 38
4.2 Ausschlusskriterien 38
5 Behandlungsplan 39
5.1 Beschreibung der Prüfmedikation MUTAGRIP® 2007/2008 (Sanofi Pasteur MSD GmbH) 39
5.1.1 Verzeichnis der Nebenwirkungen und Wechselwirkungen 40
5.2 Beschreibung der Prüfmedikation Sargramostim (Leukine®, Bayer Health Care ™) 41
5.2.1 Verzeichnis der Nebenwirkungen und Wechselwirkungen 42
5.3 Isotonische Kochsalzlösung (Braun) 43
5.3.1 Verzeichnis der Nebenwirkungen und Wechselwirkungen 43
5.4 Prüfpräparate und ihre Zubereitung 43
5.4.1 Behandlungsschema 43
5.4.2 Prüfpräparate 44
5.4.3 Bezeichnung und Materialien 44
5.4.4 Volumenberechnung 46
5.4.5 Dosierung der Prüfpräparate 47
5.5 Kriterien für einen Behandlungsabbruch 47
5.5.1 Vorzeitiger Studienabbruch eines einzelnen Patienten 47
5.5.2 Vorzeitiger Abbruch der gesamten klinischen Prüfung 47
5.6 Lagerung, Aus- und Rückgabe 48
5.7 Compliance 48

5.8 Placebo / Vergleichsmedikation ... 48
5.9 Begleitmedikation / Begleittherapie ... 48
5.10 Verblindung und Notfallumschläge ... 48
5.11 Entblindung (Brechung des Codes) ... 49
6 Studienablauf ... 49
6.1 Verfahren des „Run-In“ (Prä-Screening) ... 49
6.1.1 Verfahren der Einwilligung nach Aufklärung ... 50
6.1.2 Verfahren zur Vermeidung von zeitgleichem Einschluss in mehrere Studien ... 50
6.2 Verfahren des Screenings ... 50
6.3 Randomisierung (Zuteilung der Studienmedikation) (1. Visite) ... 50
6.4 Patienteneinschluss (Enrolment) ... 51
6.5 Behandlung der Studienpatienten ... 51
6.5.1 Pankreasresektion ... 51
6.5.2 Ösophagusresektion ... 54
6.5.3 Definitionen behandlungsrelevanter Infektionen ... 56
6.6 Klinische Untersuchungen und Abweichungen von der üblichen klinischen Praxis ... 58
6.6.1 1. Visite ... 59
6.6.2 2. und 3. Visite ... 59
6.6.3 4. und 5. Visite ... 59
6.6.4 6. bis 8. Visite ... 59
6.6.5 9. Visite (Abschlussuntersuchung) ... 60
6.6.6 Follow-Up ... 60
6.6.7 Laboruntersuchungen ... 60
6.6.8 Quantifizierung von Expressionsmustern ... 63
6.6.9 Alkoholmarker ... 63
6.6.10 Wissenschaftliche Begleituntersuchung ... 64
6.7 Dauer der Studienteilnahme für den einzelnen Patienten ... 64
7 Risiko-Nutzen-Abwägung ... 65
Risiken, Nebenwirkungen, Belastungen, Vor- und Nachteile für den Teilnehmer ... 65
8 Abbruch und Weiterbehandlung ... 66
8.1 Vorzeitiger Studienabbruch eines einzelnen Patienten ... 66
8.2 Vorzeitiger Abbruch der gesamten klinischen Prüfung ... 66
8.3 Plan für die Weiterbehandlung und medizinische Betreuung nach Aus-/Abschluss ... 66
9 Unerwünschte Ereignisse ... 67
9.1 Definitionen (nach Richtlinie 2001/20/EG) ... 67
Unerwünschtes Ereignis (Adverse Event - AE) ... 67
Schwerwiegendes unerwünschtes Ereignis (Serious Adverse Event - SAE) ... 67

Verdachtsfall einer unerwarteten schwerwiegenden Nebenwirkung“ (Serious Unexpected Suspected Adverse Reaction - SUSAR) 67
9.2 Beurteilung der Intensität 68
9.3 Beurteilung des Kausalzusammenhanges 68
9.4 Dokumentation von AEs und SAEs 68
9.5 Meldung von SAEs und von Verdachtsfällen schwerwiegender unerwarteter unerwünschter Nebenwirkungen (SUSARs) 69
9.6 Ansprechpartner und Verantwortlicher für die Meldungen 71
9.7 Data Monitoring Committee 71
10 Dokumentation 72
10.1 Elektronische Prüfbögen (eCRF) 72
10.2 Prüfarztordner 72
10.3 Dokumentation der Studienmedikation (Drug Accountability) 72
11 Qualitätsmanagement 73
11.1 Überwachung des Studienablaufs und der Datenqualität 73
11.1.1 Monitoring 73
12 Dateneingabe und Datenmanagement 73
12.1 Datenerhebung / Dokumentationsbögen 74
12.2 Datenverarbeitung 74
12.3 Generierung des Pseudonyms 74
13 Statistische Analyse 74
13.1 Fallzahlschätzung 75
13.2 Randomisierung 75
13.3 Statistische Auswertung 75
13.3.1 Hypothesen 75
13.3.2 Definition der Auswertungspopulation 76
13.3.3 Auswertung primärer und sekundärer Zielparameter 76
13.3.4 Auswertung der Sicherheit 76
13.3.5 Mögliche Zwischenauswertungen 77
13.3.6 Auswertungsmethodik 77
14 Berichterstattung 77
14.1 Biometrischer Bericht 77
14.2 Abschlussbericht 78
14.3 Publikation 78
15 Ethische, rechtliche und verwaltungstechnische Aspekte 78
15.1 Rechtliche Voraussetzungen für die Studie 78
15.2 Aufbewahrung der Daten und Zugang zu den Daten 79
16 Literaturverzeichnis 81
17 Appendices 88

**Abkürzungsverzeichnis**	
A.	Arterie
AB	Antibiotika
ADCC	Antibody Dependent Cellular Cytotoxicity
AE	Adverse Event = unerwünschtes Ereignis
AMG	Arzneimittelgesetz
AP	Alkalische Phosphatase
APACHE II	Acute Physiology And Chronic Health Evaluation
ART	Addiction Research Team
ALAT	Alanin-Aminotransferase
ASAT	Aspartat-Aminotransferase
AU	Abschlussuntersuchung
AZV	Atemzugvolumen
ßHCG	humanes Choriongonadotropin
BE	Blutentnahme
BGA	Blutgasanalyse
Bili	Bilirubin
BIPAP	Bilevel Positive Airway Pressure
C	Celsius
Ca	Karzinom
CAM-ICU	Confusion Assessment Method for the Intensive Care Unit
CBA	Cytometric Bead Array-Technologie
CDC	Centers for Disease Control
CD	Cluster of Differentiation
CDT	Carbohydrat-defizientes Transferrin
CHMP	Committee For Human medicinal Products
ConA	Concanavalin A
COPD	Chronisch obstruktive Lungenerkrankung
CPAP	Continuous Positive Airway Pressure
CRO	Contract Research Organization
CYTEL	Statistische Software
d	Tag /dezi
DDS	Delirium Detection Score
Diff. BB	Differentialblutbild
d.h.	das heißt
DLT	Doppellumentubus
Dr.	Doktor
E	Einschluss
eCRF	elektronische Case Report Form
EDTA	Ethylendiamintetraessigsäure
EG	Europäische Gemeinschaft
EK	Erythrozytenkonzentrate
EMEA	European Medicines Agency
ENTR	Entrance
ETG	Ethylglucuronid.
EU	Europäische Union
EWR	Europäischen Wirtschaftsraum
FAS	Full-Analysis-Set

g	Gramm
ggf.	gegebenenfalls
GmbH	Gesellschaft mit beschränkter Haftung
GCP	Good Clinical Practice
G-CSF	Granulozyten-Kolonie stimulierender Faktor
GCS	Glasgow Coma Scale
γ-GT	Gamma-Glutamyl-Transferase
GM-CSF	Granulozyten-Makrophagen-Kolonie stimulierender Faktor
h	Stunde
HAP	Hospital Acquired Pneumonia
HBS-Ag	Hepatitis B Surface Antigen
HCV	Hepatitis C Virus
HF	Herzfrequenz
HIV	Humanes Immundefizienz-Virus
HLA-*DR*	Humanes Leukozytenantigen
HTLV1	Humanes T-Zellleukämievirus 1
HPRT	Hypoxanthin-Phosphoribosyl-Transferase
ICH GCP	International Conference on Harmonisation Good Clinical Practice
ICU	Intensive Care Unit
i.d.R.	in der Regel
IFN-γ	Interferon-gamma
IL	Interleukin
IMPD	Investigational Medicinal Product Dossier
inkl.	inklusive
ITBVI	intrathorakaler Blutvolumen Index
ITT	Intent to treat Population
i.v.	intravenös
Kap.	Kapitel
kcal	Kilokalorien
kg	Kilogramm
KG	Körpergewicht
KKS	Koordinierungszentrum für klinische Studien
KOD	Kolloidosmotischer Druck
l	Liter
LPS	Lipoploysaccharid
m	Meter
MCV	Mittleres Corpuskuläres Volumen
ml	Milliliter
mm	Millimeter
mmHG	Millimeter Quecksilbersäule
MS	Mitgliedsstaaten
MSD	Merck Sharp and Dohme
MSR	Macrophage Scavanger Receptor
µ	Mikro
N	Fallzahl
n	nano
NaCl	Natriumchlorid
NCBI	National Center of Biotechnology Information
Nr.	Nummer
NuDESC	Nursing Delirium Screening Scale
OP	Operation

p	Ergebnis eines statistischen Signifikanztests
Pa	Pascal
Pa_{CO2}	Kohlenstoffdioxidpartialdruck
Pa_{O2}	Sauerstoffpartialdruck
PACU	Post-Anaesthesia-Care-Unit
PBMC	Peripheral Blood Mononuclear Cell
PCA	Patient Controlled Analgesia
PCR	Polymerase-Kettenreaktion
PDA	Periduralanästhesie
PDK	Periduralkatheter
PEEP	Positive End Expiratory Pressure
PEI	Paul-Ehrlich-Institut
PERI	während
PEth	Phosphatidylethanol
pH	pondus Hydrogenii
PIP	Postinspiratorischer Druck
po	Per os
PONV	Postoperatives Nausea und Vomiting
pOP	postoperativ
POST	nach
PP	Per Protocol
PPP	Per Protocol Population
PRÄ	vor
Prof.	Professor
PRRs	Pathogen recognition receptors
PSUR	Periodic safety update reports
PTCA	Transluminale Coronare Angioplastie
PTT	Partielle Thromboplastinzeit
ROCs	Receiver Operating Characteristics
RS	Rücksprache
RR	Riva-Rocci (Blutdruck)
SAE	Severe Adverse Event
SAPS	Simplified Acute Physiology Score
SAS	Statistical Analysis System
s.c.	subkutan
SE	Studienende
SecuTrial	Web based data entry für klinische Studien
SIRS	Systemisches inflammatorisches Response Syndrom
SM	Studienmedikament
SNP	Single Nucleotide Polymorphism
SOCS	Suppressor Of Cytokine Signaling
SOFA	Sepsis-related Organ Failure Assessment
SOP	Standard Operating Procedure
S-Plus 2000	Programmiersprache von Bell Laboratories zur Verarbeitung stat. Daten

SPSS	Statistical Package for the Social Sciences
StatXact 5	Statistisches Programm zur Datenanalyse
STIKO	Ständige Impfkommission
SUSAR	Suspected Unexspected Serious Adverse Drug Reaction
sVEGFR1	soluble Vascular Endothelial Growth Factor Receptor 1
SVR	Systemvaskulärer Widerstand
TGF	Transforming Growth Factor
tgl.	täglich
Th-Zelle	T-Helferzelle
TLR	Toll-like-Rezeptor
TNF-alpha	Tumor-Nekrose-Faktor alpha
Treg	Regulatorische T-Zelle
TISS	Therapeutic Intervention Scoring System
TIVA	Totale Intravenöse Anästhesie
U	Unit
V	fünf
V.	Vene
vOP	vor der Operation
Vs.	versus
WHO	World Health Organization
z.B.	Zum Beispiel
ZVD	Zentralvenöser Druck
ZVK	Zentraler Venenkatheter

Synopsis

**Titel der Studie**	Einfluss einer postoperativen Vakzination oder von GM-CSF bei immunsupprimierten Patienten nach Pankreas- oder Ösophagusresektion auf den Verlauf einer Immunsuppression und die postoperative Infektionsrate.“
**Art des Vorhabens**	Monozentrische, prospektive, dreiarmige, placebokontrollierte, randomisierte Doppelblindstudie im Double-Dummy-Design (Klinische Prüfung nach dem AMG der Phase III)
**Sponsor**	Charité, Universitätsmedizin – Berlin<br>Charitéplatz 1<br>10117 Berlin<br>Tel.: 030-450 570 142<br>Fax: 030-450 570 914<br>E-Mail: gerrit.fleige@charite.de
**Vertreter des Sponsors, medizinischer Experte und Hauptptüfer der klinischen Prüfung**	Prof. Dr. med. Claudia Spies<br>Universitätsklinik für Anästhesiologie mit Schwerpunkt operative Intensivmedizin<br>Charité – Universitätsmedizin Berlin<br>Charité Campus Virchow Klinikum<br>Augustenburger Platz 1, 13353 Berlin<br>Charité Campus Mitte<br>Charitéplatz 1, 10117 Berlin<br>Tel.: 030-450 55 11 02<br>Fax: 030-450 55 10 19<br>E-mail: claudia.spies@charite.de
**Prüfarzt**	Alawi Lütz, Alexander Schiemann, Lilit Sargsyan, Marco Paupers, Ulrike Wittkowski, Markus Renius<br>Universitätsklinik für Anästhesiologie mit Schwerpunkt operative Intensivmedizin<br>Charité – Universitätsmedizin Berlin,<br>Charité Campus Virchow Klinikum<br>Augustenburger Platz 1, 13353 Berlin<br>Charité Campus Mitte<br>Charitéplatz 1, 10117 Berlin<br>Tel.: 030-450 531 025<br>Fax: 030-450 551 019<br>E-mail: markus.renius@charite.de
**Hypothesen**	Primärhypothese: Eine postoperative Vakzinierung mit Influenza-Impfstoff oder eine postoperative Behandlung mit Sargramostim im Stadium der schweren Immunsupression führt, im Vergleich zur Placebogabe, zu einer suffizienteren

	Immunreaktivität bei Ausbleiben einer Immunparalyse, gemessen an der Normalisierung der monozytären HLA-DR Expression (> 10.000 Antigene/Monozyt).<br><br>Sekundärhypothese: Die Verabreichung von Sargramostim oder Influenza-Impfstoff führt zu einer Normalisierung der Zytokinproduktion, des Th1/Th2-Verhältnisses, des Th17/Treg-Verhältnisses sowie weiterer Immunfunktionsparameter (siehe auch Kap. 6.7.4). Die Expression spezifischer Transkriptionsfaktoren sowie die Synthese der entsprechenden Proteine normalisiert sich unter der Gabe oben genannter Prüfpräparate signifikant schneller als in der Kontrollgruppe.<br>Die Normalisierung der monozytären HLA-DR-Expression korreliert mit einer signifikanten Reduktion postoperativer Infektionen und der Verbesserung weiterer klinischer Parameter (APACHE-II-Score, SAPS-II-Score, SOFA-Score, TISS-28-Score).
**Fragestellung aufbauend auf wissenschaftlichem Hintergrund**	Postoperative Immunsuppression spielt eine wesentliche Rolle bei der Entstehung postoperativer Infektionen, welche bei immunsupprimierten Patienten 2- bis 5-fach erhöht ist [1; 2]. Dabei tritt nach größeren operativen Eingriffen entsprechend dem Gewebeschaden regelhaft unmittelbar postoperativ eine veränderte Immunreaktivität auf [1]. Bei alkoholkranken Patienten ist die Immunreaktivität bereits am ersten postoperativen Tag in Richtung Antiinflammation verschoben und führt darüber im Falle einer Immunparalyse, im Median, 3 bis 5 Tage später zu einer Infektion [2-5]. Eine postoperative Immundepression bis zur Entwicklung einer Immunparalyse kann durch Messung der HLA-DR Expression auf Monozyten nachvollzogen werden. Ein Abfall der HLA-DR Expression korreliert hoch signifikant mit dem Auftreten postoperativer Infektionen und dem Outcome der Patienten [6; 7]. Darüber hinaus konnte in mehreren Studien eine postoperative Dysbalance der T-Zell vermittelte Immunreaktivität nachgewiesen werden. Dabei ist operativer Stress postoperativ mit einer Imbalance der Th1/Th2-Antwort zu Gunsten der Th2-Antwort assoziiert [2; 8-10]. Th1-Zytokine fallen ab, während die Th2- Zytokinproduktion unbeeinflusst bleibt oder sogar gesteigert wird [8; 10].<br>In verschiedenen Studien konnte durch Impfung immunstimulierende Effekte beobachtet werden. In einem Maus-Parasiten-Modell wurde durch Vakzination eine gemischte Th1/Th2-Antwort induziert [11]. Daneben wurden auch erhöhte IFN-γ-Spiegel sowie erhöhte IL-10-Spiegel gemessen [11]. In einem weiteren Maus-Modell gelang die Rekrutierung von Th-1-Zellen in der Lunge durch nasale Immunisierung mit einem Influenza-Impfstoff [12].<br>Auch in klinischen Studien konnte eine Verbesserung der zellvermittelten Immunität durch Impfung erzielt werden [13]. Neben Th1-produzierten Zytokinen wurde auch die Produktion Th2- abhängiger Zytokine stimuliert [14]. In einer Untersuchung an seronegativen Probanden wurde durch HIV-Immunisierung eine dominante Th-1-Antwort erzielt [15].

	Blazevic et al. konnten zeigen, dass durch eine Influenza-Impfung eine vermehrte HLA-DR Expression auf Monozyten erreicht werden kann [16]. Dies konnte in eigenen Arbeiten bestätigt werden. Durch Vakzination mit Influenza-Impfstoff kam es zu einem Anstieg der HLA-DR Expression auf Monozyten bei alkoholkranken Patienten [17].<br>GM-CSF aktiviert Makrophagen und steigert deren Phagozytosefähigkeit [18]. Kurt-Jones et al. konnten in vitro durch die Gabe von GM-CSF eine vermehrte TLR2 Expression auf neutrophilen Granulozyten erreichen, was mit einer verbesserten Funktionsfähigkeit assoziiert war [19].<br>In einer klinischen Studie bei Patienten mit einer Ösophagusresektion wurde durch die prophylaktische Gabe von Filgrastim die TH1/TH2 Ratio gesteigert und die Inzidenz postoperativer Infektionen gesenkt [20]. Eine immunstimulierende Wirkung von G-CSF und GM-CSF, gemessen anhand der HLA-DR Expression auf Monozyten wurde sowohl bei septischen Patienten als auch bei Patienten nach Traumen nachgewiesen. Diese war mit einer geringeren Morbidität und Mortalität der Patienten assoziiert [20-23]. Bei Patienten nach kardiopulmonalen Bypass und schweren Trauma konnte durch GM-CSF die monozytäre HLA-DR Expression in vitro erhöht werden [24; 25].<br>Bisher wurden keine klinischen Untersuchungen durchgeführt, in denen die Vakzinierung mit Influenza-Impfstoff oder die Gabe von Sargramostim im Stadium der schweren postoperativen Immundepression, vor Beginn klinischer Zeichen einer Infektion, geprüft wurde. Eine Vakzinierung mit Influenza-Impfstoff oder die Gabe von Sargramostim im Stadium der schweren postoperativen Immundepression könnte zu einer verbesserten zellulären Immunreaktivität führen, die dann zu einer signifikanten Reduktion postoperativer Infektionen und anderer Komplikationen führt. Eine damit möglicherweise verbundene Verkürzung der intensivstationären Behandlung und Gesamtkrankenhausverweildauer könnte mit einem potentiell besseren Outcome für die Patienten verbunden sein.
**Prüfmedikation / Behandlungsstrategie**	Alle Studienpatienten erhalten das jeweilige Prüfpräparat als <u>Add-on-Therapie</u> zur Standardbehandlung.<br><br>Patienten <u>im 1. Studienarm (N = 20)</u> erhalten<br>Eine postoperative Vakzinierung mit dem Influenza-Impfstoff MUTAGRIP® 2009/2010 (Sanofi Pasteur MSD GmbH). Eine Impfdosis von 0,5 ml enthält gespaltene, inaktivierte Influenza-Viren1, die Antigene der folgenden Stämme beinhalten:<br>A/Brisbane/59/2007 (H1N1) – entsprechender Stamm (A/Brisbane/59/2007 [IVR-148]) 15 Mikrogramm HA2<br>A/Brisbane/10/2007 (H3N2) – entsprechender Stamm (A/Uruguay/716/2007 [NYMC X-175C]) 15 Mikrogramm HA2 B/Brisbane/60/2008 – entsprechender Stamm (B/Brisbane/60/2008) 15 Mikrogramm HA2 1 gezüchtet in befruchteten Hühnereiern aus gesunden Hühnerbeständen 2 Hämagglutinin sowie

	Pufferlösung: Natriumsalze (Natriumchlorid, Dinatriumhydrogenphosphat-Dihydrat), Kaliumsalze (Kaliumchlorid, Kaliumdihydrogenphosphat), Wasser für Injektionszwecke in Spuren Hühnereiproteine, Hühnerproteine, Neomycin, Formaldehyd und Octozinol 9. Die Zusammensetzung entspricht den jeweils aktuellen Empfehlungen der WHO (für die nördliche Hemisphäre) und der Europäischen Gemeinschaft.<br>*Dosierung: 1 x tgl. 0,5 ml s.c.* (1., 2. und 3. postoperativer Tag)<br><br>**und**<br><br>2. NaCl 0,9% (Braun) intravenös verabreicht: 24ml/24h (Laufrate 1ml/h über Perfusor; 1., 2. und 3. postoperativer Tag)<br><br>Patienten im 2. Studienarm (N = 20) erhalten<br>1. eine postoperative Behandlung mit Sargramostim (Leukine®, Bayer Health Care™). Eine Ampulle Leukine® enthält 250 µg lyophilisiertes LEUKINE® (Pulver) (= Sargramostim, Rekombinanter humaner Granulozyten-Makrophagen Kolonie-stimulierender Faktor, GM-CSF). Dieses muss zu Injektionszwecken mit 1 ml sterilem Wasser aufbereitet werden. Das aufbereitete lyophilisierte LEUKINE® enthält ausserdem 40 mg/ml Mannitol, 10 mg/ml Saccharose und 1,2 mg/ml Tromethamine.<br>*Dosierung: 250µg/m² Körperoberfläche/24h i.v., (kontinuierliche Applikation über Perfusor mit einer Laufrate von 1 ml/h*; 1., 2. und 3. postoperativer Tag)<br><br>**und**<br><br>2. NaCl 0,9% (Braun) subkutan verabreicht: *1 x tgl. 0,5 ml s.c.* (1., 2. und 3. postoperativer Tag)
**Vergleichsmedikation (Placebo)**	Patienten der Kontrollgruppe (3. Studienarm, N = 20) erhalten gemäß Studienprotokoll NaCl 0,9% (Braun)<br>1. subkutan verabreicht: *1 x tgl. 0,5 ml s.c.* (1., 2. und 3. postoperativer Tag) **und**<br>2. intravenös verabreicht: 24ml/24h (Laufrate 1ml/h über Perfusor; 1., 2. und 3. postoperativer Tag)<br><br>*Da die Frage nach möglichen Vorteilen einer postoperativen Sargramostim-Gabe/Influenza-Impfung für den Patienten Gegenstand dieser Studie ist, kann hier nicht von einem Vorenthalten einer Therapie für Patienten der Kontrollgruppe ausgegangen werden.*
**Studiendesign**	- Pilotstudie<br>- Dreiarmig<br>- Monozentrisch<br>- Prospektiv<br>- Doppelblind<br>- Randomisiert

<table>
<tr><td></td><td>- Placebokontrolliert
- Double Dummy Design

Die Verabreichung des jeweiligen Prüfpräparates erfolgt als <u>„Add-on-Therapie“</u> zu den Standards der Universitätsklinik für Anästhesiologie mit Schwerpunkt operative Intensivmedizin, Campus Virchow-Klinikum und Campus Charité Mitte, Charité – Universitätsmedizin Berlin.</td></tr>
<tr><td>Zeitplan</td><td><u>Beginn</u> der Studie: 26.10.2008
Vorgesehene <u>Dauer</u> der Rekrutierungsphase: 2 Jahre und 5 Monate
Voraussichtliches <u>Ende</u> der Studie: 31.03.2011

Ein generelles <u>„Follow-Up</u>“ wird nicht durchgeführt. Mit der Abschlussuntersuchung am 9. postoperativen Tag noch nicht abgeschlossene AEs werden im Rahmen eines „Follow-Up“ von 7 Tagen, noch nicht abgeschlossene SAEs im Rahmen eines „Follow-Up von 21 Tagen, nachverfolgt (siehe auch Kapitel 1 und 9.4).
Geschätzte Dauer der Auswertung bis zur Vorlage des Abschlussberichts: 24 Monate</td></tr>
<tr><td>Gesamtzahl Patienten</td><td>
<table>
<tr><td colspan="3">Elektive Pankreas-/Ösophagusresektion</td></tr>
<tr><td colspan="3">Randomisierung</td></tr>
<tr><td>Influenza-Impfung
+ NaCl 0,9%
N = 20</td><td>Sargramostim
+NaCl 0,9%
N = 20</td><td>NaCl 0,9 %

N = 20</td></tr>
</table>
</td></tr>
<tr><td>Studienpopulation</td><td>Die Studienpopulation setzt sich aus volljährigen, einwilligungsfähigen Patienten zusammen, die sich einer elektiven Pankreas- oder Ösophagusresektion unterziehen. In diese Studie werden sowohl männliche als auch weibliche Patienten eingeschlossen. Eine Untersuchung auf geschlechtsspezifische Unterschiede wird nicht durchgeführt.
Es handelt sich bei dieser Untersuchung um eine Pilotstudie. Eine Anpassung der Größe der Studienpopulation aufgrund eventueller „drop-outs“ erfolgt nicht.</td></tr>
<tr><td>Einschlusskriterien</td><td>- Elektive Pankreasresektion oder Ösophagusresektion
- Alter ≥ 18 Jahre
- Durchgeführte Patientenaufklärung
- Schriftliche Einwilligung (laut AMG §40 (1) 3b)
- negativer Schwangerschaftstest (ßHCG im Urin), (Run-In)
- hocheffektive Kontrazeption bei Frauen (definiert als Pearl Index < 1) oder anamnestisch mehr als 2 Jahre postmenopausal
- keine Teilnahme an einer anderen Studie nach dem AMG einen Monat vor Studieneinschluss sowie während der gesamten Studienteilnahme.</td></tr>
</table>

	- HLA-DR-Expression auf Monozyten ≤ 10.000 Antigene/Monozyt am 1. postoperativen Tag (BE 2)
**Ausschlusskriterien**	- Fehlende Einverständniserklärung<br>- Alter < 18 Jahre<br>- fehlende Bereitschaft zur Speicherung und Weitergabe pseudonymisierter Krankheitsdaten im Rahmen der klinischen Prüfung<br>- Unterbringung in einer Anstalt auf gerichtliche oder behördliche Anordnung (laut AMG §40 (1) 4)<br>- Teilnahme an einer anderen Studie nach dem AMG<br>- Mitarbeiter der Charité<br>- Bekannte Schwangerschaft oder positiver Schwangerschaftstest (Nachweis von ßHCG im Urin), (Run-In)<br>- Stillzeit<br>- Frauen im gebärfähigen Alter ohne gesicherte Kontrazeption<br>- Angeborene oder erworbene Bluterkrankung<br>- Chemo- oder Radiotherapie innerhalb der letzten 28 Tage<br>- Leukämie<br>- Notfalloperationen<br>- Nachgewiesene Infektion in den letzten 7 Tagen vor der Operation<br>- Bekannte Hepatitis B oder C Infektion oder positiver Labortest im Rahmen des Screenings<br>- Bekannte HIV-Infektion oder positiver Labortest im Rahmen des Screenings<br>- Allergie auf einen der arzneilich wirksamen oder sonstigen Bestandteile bzw. auf einen der möglichen Produktionsrückstände der genannten Prüfpräparate<br>- Autoimmunerkrankungen<br>- Einnahme von Immunsuppressiva bis zu 4 Wochen vor Studieneinschluss<br>- Nicht therapierte Herzrhythmusstörungen<br>- Instabile Angina pectoris<br>- Symptomatische angeborene Herzfehler<br>- Thrombosen oder thrombembolische Ereignisse in der klinischen Vorgeschichte<br>- Körpergewicht < 50 Kilogramm<br>- HLA-DR-Expression auf Monozyten > 10.000 Antigene/Monozyt am 1. postoperativen Tag (BE2)<br>- Labor am Tag vor der Operation (BE0):<br><br>Thrombozyten: ≤ 100.000/µl<br>Neutrophile: ≤ 1.500/µl<br>Hämoglobin: ≤ 8g/dl<br>Bilirubin: > 2g/dl<br>Kreatinin: > 1,5g/dl<br>ASAT/ALAT: > 90U/l<br><br>Alle Kontraindikationen und Wechselwirkungen der entsprechenden Prüfpräparate wurden bei der Festlegung von Ein- und Ausschlusskriterien mitberücksichtigt und entsprechend inkludiert.

**Randomisierung**	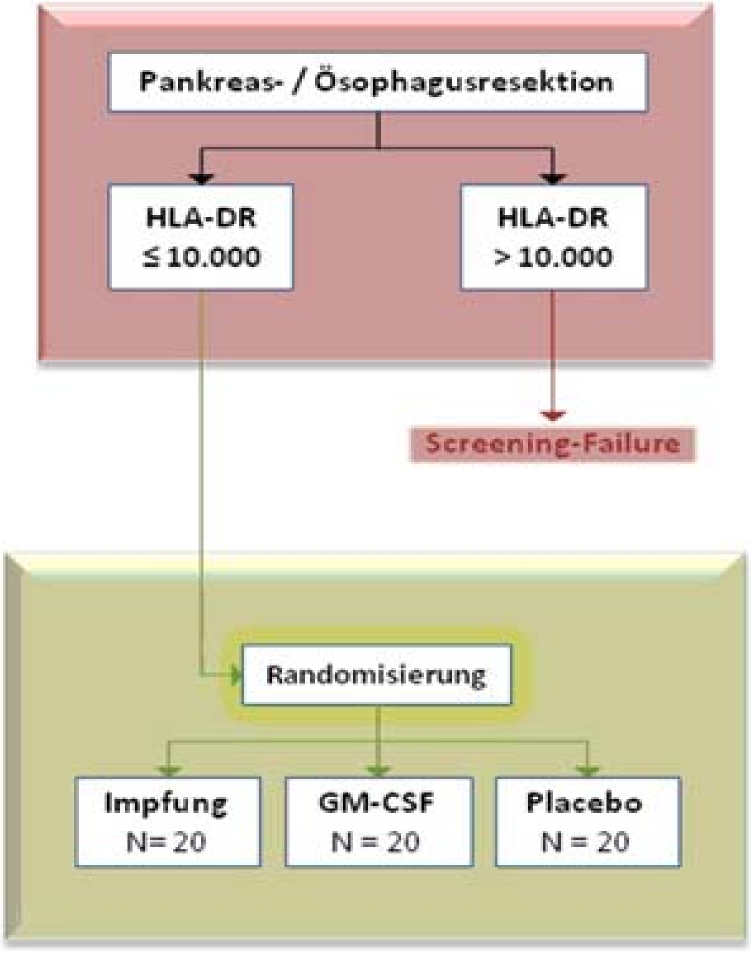<br><br>**„Screening-Phase“ „Studieneinschluss“**
**Dokumentation**	Die Daten werden in einem Papier-CRF dokumentiert.<br><br>Alle Patienten erhalten nach ihrer Einwilligung in die Studienteilnahme eine Screening-Nr. Mit dem Studieneinschluss des Patienten bekommt der Patient zusätzlich eine Randomisierungsnummer zugeteilt. Damit werden alle patientenbezogenen Daten in pseudonymisierter Form erfasst.<br>Der Prüfarzt führt eine vertrauliche Patientenidentifikationsliste, in der das jeweilige Pseudonym mit dem vollen Patientennamen verbunden ist. Jeder Patient ist durch die Pseudonymisierung unverwechselbar gekennzeichnet. Zu dieser Liste hat nur das lokale Studienteam und der Monitor Zugriff. Die Quelldokumente sowie der CRF können von Monitoren, Auditoren und Inspektoren eingesehen werden.
**Zielgrößen**	<u>Primäre Zielgröße:</u> HLA-DR-Expression auf Monozyten<br><br><u>Sekundäre Zielgrößen:</u><br>▪ Postoperative Infektionsrate<br>▪ Postoperative Delirrate (CAM-ICU, NuDESC, DDS)<br>▪ Beatmungsstunden, ITS-Liegedauer, Charité-

	Liegedauer, APACHE-II-Score, SAPS-II-Score, SOFA-Score, TISS-28-Score<br>▪ Für die ersten 33 eingeschlossenen Patienten der Behandlungsgruppen Auswertung folgender Zielparameter: 1. Zytokinproduktion im Serum, das Th_1/Th_2-Verhältnis, das Th_{17}/T_{reg}-Verhältnis, andere Immunfunktionsparameter 2. Quantitative Expression von Transkriptionsfaktoren (T-bet, Eomesodermin, GATA-3, Foxp3, RORγt, PU.1, STAT-1, STAT-3, STAT-5, NFκB) und Proteinen (SOCS-3, SOCS-1, SOCS-5, TGF-ß, IL-17, IL-6, IL-10, IFN-γ, TNF-α, IL-23, IL-10) sowie die Synthese der entsprechenden Effektorproteine
**Sicherheit**	Es gelten die intensivmedizinischen Sicherheitsparameter der Universitätsklinik für Anästhesiologie mit Schwerpunkt operative Intensivmedizin, Campus Virchow-Klinikum und Campus Charité Mitte, Charité- Universitätsmedizin Berlin. Diese intensivmedizinischen Sicherheitsparameter entsprechen den strengsten international akzeptierten Sicherheitsstandards.<br>Sämtliche schwerwiegenden unerwünschten Ereignisse (SAEs), alle unerwünschten Ereignisse (AEs) sowie alle Verdachtsfälle schwerwiegender unerwarteter unerwünschter Nebenwirkungen (SUSARs) sind zu dokumentieren, unabhängig davon, ob nach Meinung des Prüfarztes ein ursächlicher Zusammenhang mit dem Prüfmedikament besteht oder nicht. Die Dokumentation umfasst die Art des Ereignisses, Beginn, Dauer, Ausprägung/Schweregrad und Kausalität.<br>Für jedes dieser Ereignisse ist ein Berichtsbogen auszufüllen und umgehend an den Hauptprüfer weiterzuleiten. Wenn die erforderlichen Informationen zu diesem Zeitpunkt nicht verfügbar sind, müssen Folgeberichte abgefasst werden. Bei Todesfällen sollte nach Möglichkeit eine Kopie des Autopsieberichts beigelegt werden.
**Abbruchkriterien**	Ein Abbruch der Studienteilnahme für den einzelnen Patienten kann jederzeit erfolgen, wenn der Prüfarzt dies für erforderlich hält.<br>Die Abbruchkriterien der Studienteilnahme für den einzelnen Patienten sind wie folgt definiert:<br>- Widerruf der Einwilligung<br>- schwere Krankheitserscheinungen (z.B. anaphylaktischer Schock, Perikarditis, schwere Krämpfe, Vaskulitis, Enzephalomyelitis, Guillain-Barre-Syndrom, etc.) insofern nicht eine andere Ursache (z.B. chirurgische, anästhesiologische oder diagnostische Prozeduren) erkannt wird und die Entblindung des verabreichten Studienmedikamentes notwendig ist oder eine ab diesem Zeitpunkt vorgesehene Gabe von Studienmedikamenten für den Patienten von Nachteil wäre oder der Patient auf andere Art und Weise

	bezüglich der Krankheitserscheinungen und ihrer Behandlung von einem Studienabbruch profitieren würde.<br>- Eintritt einer Schwangerschaft<br>- jede andere Situation, bei der nach Ansicht des Prüfarztes eine weitere Teilnahme an der klinischen Prüfung nicht im besten Interesse des Patienten sein würde<br>- Umstände für eine therapeutische Intervention, die durch den Prüfplan nicht zugelassen sind<br>- Nachträgliches Auftreten eines Ausschlusskriteriums<br>- signifikante Protokollverletzungen (z.B. Verabreichung der Studienmedikamente nicht möglich, Nichtdurchführen der Operation, vorzeitige Entblindung, etc.)<br><br>Die gesamte Studie kann abgebrochen werden, wenn der Prüfarzt dies aus einem der folgenden Gründe für erforderlich hält:<br>- medizinische oder ethische Gründe, die die Fortsetzung der Studie gefährden<br>- Entscheidung der Studienleitung bei unvertretbaren Risiken und Toxizitäten unter Nutzen-Risiko-Abwägung<br>- deutliche Häufung von den unter 8.1. bezeichneten, einen Studienabbruch bei den Patienten notwendig machenden schweren Krankheitserscheinungen.<br>- bei unzureichender Rekrutierungsrate<br>- neue (wissenschaftliche) Erkenntnisse während der Laufzeit der klinischen Prüfung, die die Sicherheit der Studienteilnehmer gefährden können (positive Nutzen-Risiko-Abwägung nicht mehr gegeben)<br>- Auftreten von unerwünschten Ereignissen, die bisher in ihrer Art, dem Schweregrad, der Dauer oder der Häufigkeit nicht in Zusammenhang mit dem Sicherheitsprofil von MUTAGRIP® 2009/2010 oder LEUKINE® bekannt waren.
**Statistische Auswertung**	Für alle Zielgrößen werden die Befunde ausschließlich exploratorisch untersucht und deskriptiv ausgewertet, d.h. statistische Maßzahlen wie Mittelwert und Varianz (metrische Merkmale), Median und Interquartildifferenz (ordinale Merkmale) sowie erreichte Häufigkeiten bei nominalen Merkmalen bestimmt. Die erhaltenen Ergebnisse dienen zur Planung einer nachfolgenden prospektiven kontrollierten klinischen Prüfung mit entsprechender statistischer Planung der Fallzahl.<br>Möglicherweise durchgeführte statistische Test zur Prüfung der unter 13.3.1 aufgestellten Hypothesen (und weiterer) sind ausschließlich explorativer Natur, d.h. können interessante Hinweise für die wissenschaftliche Fragestellung der Studie bedeuten, sind aber nicht im konfirmatorischen Sinne zu interpretieren bzw. zu verallgemeinern. Aus dem gleichen Grunde erfolgt keine Adjustierung des Fehlers 1. Art, falls mehrfach getestet wird.<br>Die Auswertungen sind jeweils nach möglichen

	Einflussfaktoren stratifiziert durchzuführen, darüber hinaus ist eine getrennte Analyse für einzelne Strata mit einem Vergleich der Ergebnisse vorgesehen.<br>Die Auswertung der Studienergebnisse erfolgt sowohl nach dem „intent-to-treat“- als auch nach dem „per-protocol“-Prinzip.<br>Als Statistik-Software kommen die Programmpakete SAS, Version 8.2, SPSS, Version 13, S-PLUS 2000 sowie StatXact 5 von CYTEL zum Einsatz.<br><br>Die festgelegte Fallzahl von 20 Patienten je Gruppe orientiert sich ausschließlich an den Gegebenheiten der Klinik sowie den Struktureigenschaften der primären Zielgröße und wurde nicht statistisch geplant.<br>()
**Pharmakologisch-toxikologische Prüfung**	Die biologische Aktivität von **Sargramostim (GM-CSF, Leukine®)** ist artspezifisch. Folglich wurden <u>in vitro</u> Studien auf die pharmakologische Aktivität von Sargramostim an menschlichen Zellen durchgeführt. *In vitro* Inkubation von menschlichen Knochenmarkzellen mit Sargramostim in Konzentrationen von 1 bis 100 ng/ml resultiert in der Proliferation von hämatopoetischen Vorläuferzellen und in der Bildung von Granulozyten-, Makrophagen- und gemischtzelligen Granulozyten-Makrophagen-Kolonien. Die chemotaktische, fungizide und antiparasitäre Aktivität von Granulozyten und Monozyten ist nach Inkubation mit Sargramostim *in vitro* verstärkt. Desweiteren erhöht Sargramostim die Zytotoxizität von Monozyten gegen bestimmte neoplastische Zelllinien und aktiviert segmentkernige Neutrophile, um das Wachstum von Tumorzellen *in vitro* zu hemmen.<br><u>In vivo</u> Studien bei Primaten zeigten keine Toxizität bei Bolusgaben von bis zu 300µg/kgKG i.v.. In zwei weiteren Studien wurden i.v. Injektionen von bis zu 200 µg/kgKG/d für 14 Tage und s.c. Injektionen von bis zu 200µg/kgKG/d für 28Tage verabreicht. Dabei wurden keine Organschäden festgestellt (Fachinformation Leukine®, aktualisierte Fassung 12/06).<br><u>In klinischen Untersuchungen</u> wurde GM-CSF sowie G-CSF zur Prophylaxe einer postoperativen Immundeppression eingesetzt.<br>Alle mit dieser Indikation durchgeführten Studien konnten zeigen, dass Sargramostim bei Patienten im peri- und postoperativen Einsatz auf Intensivstationen sicher ist. Die Gabe von Sargramostim führt über eine Steigerung der HLA-DR-Expression zu einer Verbesserung der Immunkompetenz [22; 23]. In drei verschiedenen Studien konnte die Sicherheit der Anwendung bei septischen Patienten bestätigt werden. Auch bei septischen Neugeborenen war sowohl die Gabe von G-CSF als auch von GM-CSF sicher und führte zu einem Anstieg der HLA-DR Expression [26].<br>*Für weitere Informationen zu wichtigen, aktuell veröffentlichten Studien mit GM-CSF als Prüfpräparat, wird auf das Kapitel 2.2.2 in diesem Prüfplan verwiesen.*

	Die jährliche Anpassung der Stammzusammensetzung von **Influenza-Impfstoffen** entspricht den jeweils aktuellen Empfehlungen der WHO (für die nördliche Hemisphäre) nach Genehmigung der Empfehlung durch das CHMP (Committee For Human Medicinal Products).<br>Die Chargenfreigabe und zeitgleiche Genehmigung der Änderungsanzeige erfolgt durch das Paul-Ehrlich-Institut (PEI). Dabei erfolgt die Sicherheitsbeurteilung des Impfstoffes insbesondere auf Grundlage des „Periodic Safety Update Reports“ (PSUR) als Maß für die Pharmakovigilanz.<br>Darüber hinaus erfolgt im Rahmen der jährlichen Aktualisierung der Stammzusammensetzung, die Durchführung offener Studien ohne Kontrollgruppe durch Sanofi-Aventis. Die Studienpopulation umfasst mindestens 50 Erwachsene im Alter zwischen 18 und 60 Jahren und mindestens 50 ältere Personen über 60 Jahren einbeziehen. Die Beurteilung der Sicherheit erfolgt im Rahmen der jährlich wiederholten klinischen Studien während der ersten drei Tage nach der Impfung.<br><br>Die Sicherheit inaktivierter Grippeimpfstoffe wurde in randomisierten Studien geprüft [27-29]. In diesen Studien zeigte sich insbesondere ein erhöhtes Auftreten von lokalen Ereignissen an der Einstichstelle bei Grippeimpfstoff im Vergleich zu Placebo (17,5% vs. 7,4%, p<0,0001). Es kam jedoch nicht zu einem signifikant häufigeren Auftreten von systemischen Reaktionen im Vergleich zu Placebo (11% vs. 9,4%, p=0,39). Erfahrungen zu einer zweimaligen Impfung mit Influenza-Impfstoff liegen aus verschiedenen an unserer Klink durchgeführten Vorläuferstudien vor. Hierbei konnte unter anderem gezeigt werden, dass durch eine zweimalige Influenza-Impfung bei immunsupprimierten Patienten, die HLA-DR-Expression auf Monozyten unmittelbar und signifikant gesteigert werden konnte [17]. In einer noch nicht entblindeten, laufenden Studie an unserer Klinik (EudraCT-Nr.:2004-004352-40, EA - Nr.: 1/048/04) wurden Patienten (aktuell 63 Patienten) mit einem Tumor des oberen Aerodigestivtrakts 1 Tag vor Operation und am Operationstag vor Operationsbeginn jeweils mit 0,5 ml Influenza-Impfstoff geimpft. Hierbei wurden bis dato keine schwerwiegenden Ereignisse aufgrund der Impfung beobachtet.
**Mögliche Risiken, Nebenwirkungen, Kontraindikationen**	Im Allgemeinen ist eine Vakzination mit **Influenza-Impfstoff** sehr gut verträglich. Am häufigsten ($\geq$1% und <10%) treten lokale Reaktionen wie Rötung, Schwellung, Schmerzen, kleinflächige Hautblutungen und Verhärtungen auf. Darüber hinaus können allgemeine (systemische) Unverträglichkeiten wie Fieber, Übelkeit, Schüttelfrost, Müdigkeit, Kopfschmerz, Schwitzen, Muskel- und Gelenkschmerzen sowie Schwellung der Lymphknoten. Diese Reaktionen klingen für gewöhnlich innerhalb 1-2 Tage ohne Behandlung wieder ab. Seltene Reaktionen ($\geq$0,01% und <0,1%) sind Neuralgie, Parästhesie, Krämpfe, vorübergehende Thrombozytopenie mit vereinzelten Blutungen oder Hämatomen. Als allergische

	Reaktionen können Urtikaria, Pruritus, erythematöser Ausschlag und Dyspnoe auftreten, die in seltenen Fällen bis zum Schock führen können. Sehr selten (<0,01%, einschl. Einzelfälle) wurde über Entzündungen der Blutgefäße mit vorübergehender Beteiligung der Niere berichtet. Darüber hinaus wurde über seltene neurologische Störungen berichtet wie z.B. Enzephalomyleitis, Neuritis und Guillan-Barré-Syndrom.<br>In eigenen Vorarbeiten wurden keinerlei negative Auswirkungen durch eine zweimalige präoperative Vakzinierung mit Influenza-Impfstoff beobachtet [17].<br>MUTAGRIP® darf nicht verabreicht werden wenn die Person gegen eines der arzneilich wirksamen oder sonstige Bestandteile bzw. auf eines der möglichen Produktionsrückstände (Hühnereiproteine, Hühnerprotein, Neomycin, Formaldehyd, Octoxinol 9) allergisch/überempfindlich reagiert. Desweiteren sollte MUTAGRIP® nicht angewendet werden, wenn eine akute fieberhafte Erkrankung vorliegt.<br>Zwecks weiterer detaillierter Ausführungen zu Verdachtsfällen auf Impfkomplikationen sowie über Verdachtsfälle schwerwiegender Nebenwirkungen nach Impfung sei auf die Ausführungen zur Datenbank des Paul-Ehrlich-Instituts vom 01.07.2007 im beiliegenden vereinfachten IMPD verwiesen.<br><br>Da viele, der in klinischen Studien berichteten, unerwünschten Wirkungen mit der Grunderkrankung oder deren Therapie assoziiert sind, konnte nie ein kausaler Zusammenhang zwischen diesen Effekten und **Leukine®** nachgewiesen werden.<br>Die meisten negativen Reaktionen waren leicht oder mäßig schwer. Selten waren sie schwerwiegend oder lebensbedrohlich. Die meisten unerwünschte Wirkungen, über die häufig berichtet wurde, waren Fieber, Übelkeit Dyspnoe, Durchfall, Ausschlag, Reaktionen an der Injektionsstelle (bei s.c. Gabe), Erbrechen, Anorexie, muskuloskeletale Schmerzen und Asthenie.<br>Weniger häufig werden folgende Ereignisse berichtet: unbestimmte Thoraxschmerzen, Stomatitis, Kopfschmerz, vermehrtes Schwitzen, Unterleibsschmerz, Pruritus, Schwindel, periphere Ödeme, Parästhesien und Myalgie.<br>Leukine® wurde selten mit Pleuritis, Pleuraerguß (Verum 1% vs. Placebo 0%), Perikarditis- und/oder Perikarderguß (Verum 4% vs. Placebo 1%) assoziiert. Sollten solche Reaktionen auftreten, sollte Leukine® abgesetzt werden.<br>Patienten mit vorbestehender Lungenkrankheit können durch eine vermindernder Lungenfunktion und Dyspnoe unter der Gabe von Leukine®, insbesondere nach erstmaliger Injektion, auffällig werden und sollte engmaschig überwacht werden.<br>Die Sargramostim-Gabe ist kontraindiziert bei Patienten mit leukämischen myeloiden Blasten im Knochenmark oder im peripheren Blut von mehr als 10% sowie bei Patienten mit bekannter Hypersensitivität gegenüber GM-CSF, von Hefen abgeleiteten Produkten oder einem anderen Bestandteil des

	Produktes.<br><br>Für weitergehende Informationen wird auf die Kapitel 6.1 – 6.3 sowie das beiliegende vereinfachte IMPD verweisen.
**Risiko-Nutzen-Abwägung**	Ziel der Studie ist, durch Vakzinierung mit Influenza-Impfstoff bzw. Verabreichung von GM-CSF zum Zeitpunkt der postoperativen Immundepression die Immunreaktivität wieder zu erhöhen und somit die Immunparalyse zu verhindern welche nachweislich mit einem signifikant erhöhten Auftreten infektionsbedingter Komplikationen verbunden ist [7]. Insbesondere bei großen abdominalchirurgischen Eingriffen wie Pankreas- und Ösophagusresektionen sind postoperative Infektionsraten von bis zu 34 % und eine allgemeine Komplikationsrate von bis zu 48 % beschrieben [30-33]. Eine mögliche Reduktion der Inzidenz postoperativer Infektionen und anderer Komplikationen könnte die Morbidität und Mortalität dieser Patienten nachhaltig senken. Eine damit verbundene Verkürzung der intensivstationäre Behandlung und Gesamtkrankenhausverweildauer ist nicht nur mit einem potentiell besserem Outcome der Patienten verbunden sondern reduziert auch die Kosten für die Behandlung..<br>Beide Prüfpräparate sind hinsichtlich ihres Wirkungs- und Risikoprofils in einer Vielzahl von Studien untersucht worden. Diese belegen die Sicherheit in der Anwendung am Patienten unter Berücksichtigung der entsprechenden Kontraindikationen. Die prä, peri- und postoperative Behandlung der Patienten erfolgt nach den Standards der Universitätsklinik für Anästhesiologie mit Schwerpunkt operative Intensivmedizin – Charité Universitätsmedizin Berlin. Durch die Add-on-Therapie mit einem der Prüfpräparate wird die Standardbehandlung der Patienten in keinster Weise nachteilig beeinflusst.

1 Ablaufschema

	Run-in	Screening	Screening	1. Visite	2. Visite	3. Visite	4. Visite	5. Visite	6. Visite	7. Visite	8. Visite	9. Visite	**Follow-up**	**Follow-up**
	vOP	OP	1. pOP	1. pOP	2. pOP	3. pOP	4. pOP	5. pOP	6. pOP	7. pOP	8. pOP	9. pOP	**16. pOP**	**30. pOP**
Patientenaufklärung	✓	-		-	-	-	-	-	-	-	-	-	-	-
Einverständniserklärung	✓	-		-	-	-	-	-	-	-	-	-	-	-
Anamnese einschl. früherer Therapien	✓	-		-	-	-	-	-	-	-	-	-	-	-
Überprüfung der Ein- und Ausschlusskriterien	✓	✓		-	-	-	-	-	-	-	-	-	-	-
Klinische Untersuchung / Diagnostik	✓	✓		-	-	-	-	-	-	-	-	-	-	-
Schwangerschaftstest*	✓	-		-	-	-	-	-	-	-	-	-	-	-
Blutentnahme der Zielparameter	-	✓	✓	-	✓	✓	✓	✓	-	-	-	-	-	-
Randomisierung	-	-	-	✓	-	-	-	-	-	-	-	-	-	-
Applikation der Studienmedikation	-	-		✓	✓	✓	-	-	-	-	-	-	-	-
Abfrage der Begleitmedikation	✓	✓		✓	✓	✓	✓	✓	✓	✓	✓	✓	-	-
Abfrage intensivmedizinischer Sicherheitsparameter**	-	✓		✓	✓	✓	✓	✓	✓	✓	✓	✓	-	-
Dokumentation von AEs, SAEs und SUSARs***	✓	✓		✓	✓	✓	✓	✓	✓	✓	✓	✓	-	-
Dokumentation AE's[#4]	-	-		-	-	-	-	-	-	-	-	-	✓	-
Dokumentation SAE's[#5]	-	-		-	-	-	-	-	-	-	-	-	✓	✓
Abschlussuntersuchung[#6]	-	-		-	-	-	-	-	-	-	-	✓	-	-

*bei gebärfähigen Frauen; gebärfähig definiert als: (bis 2 Jahre nach Menopause)
**Vorherige Beendigung der Dokumentation intensivmedizinischer Sicherheitsparameter wenn Verlegung auf Normalstation: RR, HF, ZVD, Arterieller Druck, Temperatur, APACHE-II-Score, TISS-28-Score, SAPS-II, SOFA-Score
***Verfahrensweise zur Meldung der SUSARs siehe Kap. 9.5
[#4]Betrifft bis Studienende (9. pOP Tag) noch nicht abgeschlossene AEs (siehe auch Kap. 9.4 im Prüfplan)
[#5]Betrifft bis Studienende (9. pOP Tag) noch nicht abgeschlossene SAEs (siehe auch Kap. 9.4 im Prüfplan)
[#6] Im Falle eines vorzeitigen Studienabbruches ist die Abschlussuntersuchung zum Zeitpunkt des Studienabbruches durchzuführen

2 Einleitung

2.1 Einleitung und Hintergrund zur klinischen Prüfung

Bisher wurden keine klinischen Untersuchungen durchgeführt, in denen die Vakzinierung mit Influenza-Impfstoff oder die Gabe von Sargramostim im Stadium der schweren postoperativen

Immundepression, vor Beginn klinischer Zeichen einer Infektion, geprüft wurde. Eine Vakzinierung mit Influenza-Impfstoff oder die Gabe von Sargramostim im Stadium der schweren postoperativen Immundepression könnte zu einer verbesserten zelluläre Immunreaktivität führen, die dann zu einer signifikanten Reduktion postoperativer Infektionen und anderer Komplikationen führt. Eine damit möglicherweise verbundene Verkürzung der intensivstationären Behandlung und Gesamtkrankenhausverweildauer könnte mit einem potentiell besseren Outcome für die Patienten verbunden sein.

Postoperative Immunsuppression spielt eine wesentliche Rolle bei der Entstehung postoperativer Infektionen, welche bei immunsupprimierten Patienten 2- bis 5-fach erhöht ist [1; 2]. Dabei tritt nach größeren operativen Eingriffen entsprechend dem Gewebeschaden regelhaft unmittelbar postoperativ eine veränderte Immunreaktivität auf [4]. Bei alkoholkranken Patienten ist die Immunreaktivität bereits am ersten postoperativen Tag in Richtung in Richtung Antiinflammation verschoben und führt darüber im Falle einer Immunparalyse, im Median, 3 bis 5 Tage später zu einer Infektion [2-5]. Eine postoperative Immundepression bis zur Entwicklung einer Immunparalyse kann durch Messung der HLA-DR Expression auf Monozyten nachvollzogen werden. Der Abfall der HLA-DR Expression korreliert statistisch signifikant mit dem Auftreten infektionsbedingter Komplikationen [7].
Identische Beobachtungen konnten auch bei Kindern, die sich einem elektiven kardiochirurgischem Eingriff unterzogen, gemacht werden [6].
Darüber hinaus konnte in mehreren Studien eine postoperative Dysbalance der T-Zell vermittelte Immunreaktivität nachgewiesen werden. Dabei ist operativer Stress postoperativ mit einer Imbalance der Th1/Th2-Antwort zu Gunsten der Th2-Antwort assoziiert [2; 8-10]. Th1-Zytokine fallen ab, während die Th2- Zytokinproduktion unbeeinflusst bleibt oder sogar gesteigert wird [8; 10].
Eine besondere Bedeutung bei der Erkennung von Pathogenen und der Einleitung einer adäquaten Abwehrreaktion kommt den Toll-like-Rezeptoren (TLR) zu. Derzeit sind beim Menschen 10 TLR identifiziert. Insbesondere TLR2 und TLR4 sind für die Abwehr von Pathogenen von entscheidender Bedeutung. Während TLR2 insbesondere Bestandteile der Wand von gram-positiven Bakterien erkennt, werden gram-negative Keime von TLR4 detektiert [34-38]. Mutationen von TLR 2 und 4 erhöhen das Risiko bakterieller Infektionen [39; 40]. Eine reduzierte Produktion proinflammatorischer Zytokine durch Monozyten scheint bei den Patienten mit Polymorphismen mitverantwortlich zu sein [41]. TLR Mutationen sind von großer klinisch Relevanz, da ca. 10% der Bevölkerung Mutationsträger sind [42].

Eine schwere Immundepression oder Immunparalyse ist bereits am ersten postoperativen Tag mittels Bestimmung der HLA-DR Expression auf Monozyten nachweisbar. Ein Abfall der HLA-DR Expression korreliert hoch signifikant mit dem Auftreten postoperativer Infektionen und dem Outcome der Patienten [6; 7].
In verschiedenen Studien konnte durch Impfung immunstimulierende Effekte beobachtet werden. In einem Maus-Parasiten-Modell wurde durch Vakzination eine gemischte Th1/Th2-Antwort induziert [11]. Daneben wurden auch erhöhte IFN-γ-Spiegel sowie erhöhte IL-10-Spiegel gemessen [11]. In einem weiteren Maus-Modell gelang die Rekrutierung von Th-1-Zellen in der Lunge durch nasale Immunisierung mit einem Influenza-Impfstoff [12].

Auch in klinischen Studien konnte eine Verbesserung der zellvermittelten Immunität durch Impfung erzielt werden [13]. Neben Th1-produzierten Zytokinen wurde auch die Produktion von Th2 abhängigen Zytokinen [14]. In einer Untersuchung an seronegativen Probanden wurde durch HIV-Immunisierung eine dominante Th-1-Antwort erzielt [15].

Blazevic et al. konnten zeigen, dass durch eine Influenza-Impfung eine vermehrte HLA-DR Expression auf Monozyten erreicht werden kann [16]. Dies konnte in eigenen Arbeiten bestätigt werden. Durch Vakzination mit Influenza-Impfstoff kam es zu einem Anstieg der HLA-DR Expression auf Monozyten bei alkoholkranken Patienten [17].

GM-CSF aktiviert Makrophagen und steigert deren Phagozytosefähigkeit [18]. Kurt-Jones et al. konnten in vitro durch die Gabe von GM-CSF eine vermehrte TLR2 Expression auf neutrophilen

Granulozyten erreichen, was mit einer verbesserten Funktionsfähigkeit assoziiert war [19]. In einer klinischen Studie bei Patienten mit einer Ösophagusresektion wurde durch die prophylaktische Gabe von Filgrastim die TH1/TH2 Ratio gesteigert und die Inzidenz postoperativer Infektionen gesenkt [20]. Eine immunstimulierende Wirkung von G-CSF und GM-CSF, gemessen anhand der HLA-DR Expression auf Monozyten wurde sowohl bei septischen Patienten als auch bei Patienten nach Traumen nachgewiesen. Diese war mit einer geringeren Morbidität und Mortalität der Patienten assoziiert [20-23]. Bei Patienten nach kardiopulmonalen Bypass und schweren Trauma konnte durch GM-CSF die monozytäre HLA-DR Expression in vitro erhöht werden [24; 25].

2.2 Stand des Wissens über die Prüfpräparate

2.2.1 Influenza-Impfstoff: MUTAGRIP® (Sanofi Pasteur MSD GmbH)

Aufgrund der Modulierbarkeit der Influenzavirus-Hämagglutinine und der Möglichkeit des Antigenshifts ist es offensichtlich, dass alle Formen der Lagerhaltung *(stockpiling)* von Impfstoffen letztendlich keinen Sinn ergeben. Aller Voraussicht nach würde eine von im Voraus produzierten Impfstoffen induzierte Immunantwort in ihrer Spezifität nur ungenügend oder gar nicht einem veränderten humanpathogenen Influenzavirus entsprechen. Daraus ergibt sich die absolute Notwendigkeit der jährlichen Änderung der bestehenden Zulassung für den entsprechenden Influenzaimpfstoff, welche die Änderungen in der Stammzusammensetzung berücksichtigt.
Die jährliche Anpassung der Stammzusammensetzung von Influenza-Impfstoffen (MUTAGRIP®) entspricht den jeweils aktuellen Empfehlungen der WHO (für die nördliche Hemisphäre) nach Genehmigung der Empfehlung durch das CHMP (Committee For Human Medicinal Products).
Die Chargenfreigabe und zeitgleiche Genehmigung der Änderungsanzeige erfolgt durch das Paul-Ehrlich-Institut (PEI). Dabei erfolgt die Sicherheitsbeurteilung des Impfstoffes insbesondere auf Grundlage des „Periodic Safety Update Reports“ (PSUR) als Maß für die Pharmakovigilanz.
Darüber hinaus erfolgt im Rahmen der jährlichen Aktualisierung der Stammzusammensetzung, die Durchführung offener Studien ohne Kontrollgruppe durch Sanofi-Aventis. Die Studienpopulation umfasst mindestens 50 Erwachsene im Alter zwischen 18 und 60 Jahren und mindestens 50 ältere Personen über 60 Jahren einbeziehen. Die Beurteilung der Sicherheit erfolgt im Rahmen der jährlich wiederholten klinischen Studien während der ersten drei Tage nach der Impfung.

<u>Pharmakodynamische Eigenschaften</u>

Der Impfschutz tritt in der Regel innerhalb von 2 – 3 Wochen ein. Die Schutzdauer gegen die im Impfstoff verwendeten Stämme oder entsprechende Stämme ist unterschiedlich, beträgt jedoch im Allgemeinen 6 Monate bis 1 Jahr.

<u>Pharmakokinetische Eigenschaften</u>

Es wurden keine tierexperimentellen Studien zur Embryotoxizität und Teratogenität durchgeführt.

<u>Anwendungsgebiete</u>

In der alltäglichen Praxis dient die Impfung mit einem Influenza-Impfstoff (mit aktueller von der WHO empfohlener Antigenkombination) der Vorbeugung der echten Virusgrippe. Gemäß Fachinformation wird die Impfung insbesondere Personen mit erhöhtem Risiko für Influenza-bedingte Komplikationen empfohlen.
Die STIKO empfiehlt eine Impfung mit einem Influenza-Impfstoff wie folgt [43]:

S	Personen über 60 Jahre
I	Kinder, Jugendliche und Erwachsene mit erhöhter gesundheitlicher Gefährdung infolge eines Grundleidens, wie z.B.:<br>▪ chron. Erkrankung der Atmungsorgane (inkl. Asthma und COPD)<br>▪ chron. Herz-Kreislauf- Leber- und Nierenkrankheiten, Diabetes und andere Stoffwechselkrankheiten<br>▪ Multiple Sklerose mit durch Infektionen getriggerten Schüben<br>▪ Personen mit angeborenen oder erworbenen Immundefekten<br>▪ Personen mit T- und/oder B-zellulärer Restfunktion<br>▪ HIV-Infektionen<br>▪ Bewohner von Alters- oder Pflegeheimen
B/I	Personen mit erhöhter Gefährdung, z.B. medizinisches Personal, Personen in Einrichtungen mit umfangreichen Publikumsverkehr sowie Personen, die als mögliche Infektionsquelle für von ihnen betreute ungeimpfte Risikopersonen fungieren können.
I/B	Personen mit erhöhter Gefährdung durch direkten Kontakt zu Geflügel oder Wildvögeln
R/I	Für Reisende aus den unter S und I genannten Personengruppen, die nicht über einen aktuellen Impfschutz verfügen; für andere Reisende ist eine Impfung nach Risikoabwägung entsprechend Exposition und Impfstoffverfügbarkeit sinnvoll.
I	Wenn eine intensive Epidemie aufgrund von Erfahrungen in anderen Ländern droht oder nach deutlicher Antigendrift bzw. einer Antigenshift zu erwarten ist und der Impfstoff die neue Variante enthält.

I = Indikationsimpfungen für Risikogruppen bei individuell (nicht beruflich) erhöhtem Expositions-, Erkrankungs- oder Komplikationsrisiko sowie auch zum Schutz Dritte; B = Impfung aufgrund eines erhöhten beruflichen Risikos; R = Impfung aufgrund von Reisen; P = Postexpositionelle Prophylaxe/Regelungsimpfung bzw. andere Maßnahmen der spezifischen Prophylaxe bei Kontaktpersonen in Familie und Gemeinschaft

Klinische Untersuchungen

Die Sicherheit inaktivierter Grippeimpfstoffe wurde in randomisierten Studien geprüft [27-29; 44]. In diesen Studien zeigte sich insbesondere ein erhöhtes Auftreten von lokalen Ereignissen an der Einstichstelle bei Grippeimpfstoff im Vergleich zu Placebo (17,5% vs. 7,4%, p<0,0001). Es kam jedoch nicht zu einem signifikant häufigeren Auftreten von systemischen Reaktionen im Vergleich zu Placebo (11% vs. 9,4%, p=0,39).
Immunsuppression oder immunsuppressive Behandlungen können den Impferfolg beeinträchtigen.

Dieser Impfstoff kann gleichzeitig mit anderen Impfstoffen an unterschiedlichen Körperstellen verabreicht werden. Bei gleichzeitiger Verabreichung verschiedener Impfstoffe kann es möglicherweise verstärkt zu Nebenwirkungen kommen.

Nach Impfungen gegen Influenza wurden falsch positive Ergebnisse serologischer Tests auf Antikörper gegen HIV1, Hepatitis C und besonders gegen HTLV1 beobachtet, die mittels ELISA durchgeführt wurden. Im Western-Blot-Verfahren sind verfälschte Ergebnisse nicht beobachtet worden. Die vorübergehenden falsch positiven Reaktionen könnten auf eine Ig-M Antwort auf den Impfstoff zurückzuführen sein.
Klinische Untersuchungen konnten eine Verbesserung der zellvermittelten Immunität nach Influenza-Impfung belegen [13]. Neben Th1-Zytokinen wie IL-2 und IFN-γ konnte auch eine erhöhte Produktion von Th2-abhängigen Zytokinen wie IL-4 und IL-10 beobachtet werden [14]. In einer anderen Untersuchung an seronegativen Probanden wurde durch HIV-Immunisierung eine Th1-dominante Antwort erzielt [15].

Vorerfahrungen des Antragsstellers mit Influenza-Impfung bei Immundepression

Es liegen bereits Erfahrungen bzgl. Einer mehrmaligen Impfung mit dem Influenza-Impfstoff MUTAGRIP im Rahmen von zwei an unserer Klinik durchgeführten klinischen Vorläuferstudien vor. In einer bereits abgeschlossenen Studie konnte im Zusammenhang mit einer zweimaligen präoperativen Vakzinierung mit MUTAGRIP keine Verdachtsfälle unerwarteter schwerwiegender Nebenwirkungen (SUSAR) beobachtet werden.
Bereits in dieser Studie konnten wir eine signifikante Steigerung der HLA-DR-Expression auf Monozyten durch Influenza-Impfung erzielen [17]. Der Effekt war jedoch ausschließlich bei konservativ behandelten Patienten sichtbar. Jene Patienten, welche sich einer operativen Tumorentfernung unterzogen, zeigten postoperativ keine signifikante Steigerung der HLA-DR-Expression auf Monozyten. Möglicherweise wurde eine durch Impfung induzierte Immunstimulation bei diesen Patienten durch den operativen Eingriff überdeckt. Möglicherweise könnte diese Patientenkollektiv von einer postoperativen Intervention mit Influenza-Impfstoff profitieren. In einer noch laufenden, nicht entblindeten Studie an unserer Klinik (EudraCT-Nr.: 2004-004352-40, EA-Nr.: 1/048/04) wurden Patienten (aktuell 63) mit einem Tumor des oberen Aerodigestivtraktes jeweils am Tage vor der Operation sowie am Operationstag unmittelbar vor Operationsbeginn mit einer Dosis Influenza-Impfstoff geimpft. Im Verlauf der Studie wurden bis dato keine SUSARs im Zusammenhang mit der Impfung beobachtet.

2.2.2 Sargramostim (=GM-CSF, Leukine®, Bayer Health Care™)

Pharmakodynamische Eigenschaften

Kolonie-stimulierende Faktoren sind Zytokine, die über eine Signalkaskade in die Proliferation und Differenzierung von hämatopoetischen Zellen eingreifen.
GM-CSF wirkt über die Bindung an spezifische Rezeptoren die von den Zielzellen auf der Zelloberfläche exprimiert werden. GM-CSF (Sargramostim, Molgramostim) hat ein weitaus breiteres Wirkungsspektrum als G-CSF, welches sich sowohl auf die Vorläuferzellen der Granulozyten, Makrophagen und Eosinophilen als auch auf diese Zellen selbst erstreckt.

Pharamakokinetik

Die Pharmakokinetik ist in einer kontrollierten Studie an 24 männlichen gesunden Freiwilligen untersucht worden (Leukine® (Sargramostim) [package insert], 2007). Hierbei betrug die β-Halbwertszeit nach zweistündiger i.v-Gabe von 250µg/m² circa 60 Minuten. Die maximalen Konzentrationen von GM-CSF im Serum wurden während oder sofort nach der vollständigen Sargramostim -Gabe gemessen.

Anwendungsgebiete

Für GM-CSF (Sargramostim) besteht derzeit in Deutschland keine Zulassung.
In den USA ist Leukine® derzeit für folgende Indikationen zugelassen:

- zur Verkürzung der Dauer der Neutropenie nach myelosuppressiver Chemotherapie sowie
- zur Verkürzung der Neutropenie nach myeloablativer Chemotherapie mit anschließender Knochenmark- oder Blutstammzelltransplantation.

In vitro Studien

Die biologische Aktivität von GM-CSF ist artspezifisch. Folglich wurden *in vitro* Studien auf die pharmakologische Aktivität von Sargramostim an menschlichen Zellen durchgeführt. *In vitro* Inkubation von menschlichen Knochenmarkzellen mit Sargramostim in Konzentrationen von 1 bis100 ng/ml resultiert in der Proliferation von hämatopoetischen Vorläuferzellen und in der Bildung von Granulozyten-, Makrophagen und gemischten Granulozyten-Makrophagen-Kolonien. Die chemotaktische, fungizide und anti-parasitäre Aktivität von Granulozyten und Monozyten ist nach Inkubation mit Sargramostim in vitro verstärkt [24].

Desweiteren erhöht Sargramostim die Zytotoxizität von Monozyten gegen bestimmte neoplastische Zelllinien und aktiviert segmentkernige Neutrophile, um das Wachstum von Tumorzellen *in vitro* zu hemmen [25].

In vivo Studien

In vivo-Studien an Primaten zeigten keine Toxizität bei Bolusgaben von bis zu 300µg/kg/KG i.v. . In zwei weitern Studien wurden i.v. Injektionen von bis zu 200 µg/kgKG/d für 14 Tage und s.c. Injektionen von bis zu 200µg/kgKG/d für 28Tage verabreicht. Dabei traten keine Organschäden auf. Phytopathologisch zeigte sich eine gesteigerte Zellularität in den hämatologischen Organen sowie in Herz- und Lungengewebe. Während des Behandlungszeitraums trat eine dosisabhängige Zunahme der Leukozyten auf, welche hauptsächlich aus segmentkernigen neutrophilen Granulozyten bestand., Zudem wurde eine Zunahme von Monozyten, basophilen und eosinophilen Granulozyten sowie Lymphozyten beobachtet. 1-2 Wochen nach Behandlung erreichte die Leukozytenzahl wieder Ausgangswerte.

Klinische Erfahrungen bei Patienten mit Immunsuppression

Da die pharmakologischen Wirkungen von GM-CSF und G-CSF sich über weite Teile überschneiden [45-47], werden diese hier sowohl im Text als auch im weiteren tabellarisch gemeinsam aufgeführt.

Eine postoperative Immunsuppression, wie sie unter anderem nach großen abdominal–chirurgischen Eingriffen vermehrt auftritt, prädisponiert zur Entwicklung nosokomialer Infektionen wie Pneumonien, Wundinfektionen und Sepsis. In klinischen Untersuchungen wurde GM-CSF sowie G-CSF zur Prophylaxe einer postoperativen Immundeppression eingesetzt.
Alle mit dieser Indikation durchgeführten Studien konnten zeigen, dass Sargramostim bzw. Filgrastim bei Patienten im peri- und postoperativen Einsatz auf Intensivstationen sicher ist. Es wurde über einen Anstieg der Leukozytenzahl, gelegentliche Knochenschmerzen sowie temporäre Hautausschläge berichtet [48-53]. In einer Vielzahl von Studien konnte eine Reduktion der Infektionsrate erreicht werden [23; 50; 51; 54].
In einer Studie bei Schädel-Hirn-Verletzten sank die Rate der Bakteriämien dosisabhängig [55]. Die Gabe von Sargramostim führt über eine Steigerung der HLA-DR-Expression zu einer Verbesserung der Immunkompetenz [22; 23]. In drei verschiedenen Studien konnte die Sicherheit der Anwendung bei septischen Patienten bestätigt werden. Auch bei septischen Neugeborenen war sowohl die Gabe von G-CSF als auch von GM-CSF sicher und führte zu einem Anstieg der HLA-DR Expression [26].
In einer Studie des Instituts für medizinische Immunologie der Charité- Universitätsmedizin Berlin, Campus Charité Mitte, die in Kooperation mit unserer Klinik durchgeführt wurde, wurde 19 septischen Patienten GM-CSF für 4 Tage in einer Dosis von 8µg/kgKG verabreicht. Dabei traten keine negativen Effekte auf, die der Studienmedikation zugeordnet werden konnten. Auch hier kam es unter GM-CSF-Behandlung zu einem signifikanten Anstieg der monozytären HLA-DR Expression sowie der TNF-α-Produktion.

Quelle	IP	Dosierung	Placebo	Population	N	Ergebnisse
Ahmad A et al., Pediatr Infect Dis J 2002 [56]	GM-CSF (I) G-CSF (II)	2 x 4 µg/kgKG/d (II) 2 x 5µg/kgKG/d (II)	ja (III)	Septische Neugeborene Dauer 5 Tage Alter 24,5 Wochen (23-27) (I) / 28,5 (24-31) (II) / 27,5 (25-31)	10 (I) 10 (II) 8 (III)	Keine negative Effekte im Vergleich zu Placebo Die Neutrophilen stiegen mit G-CSF schneller an
Apte SM et al., J Transl Med 2006 [57]	GM-CSF + IFNγ-1b	Beginn mit 400 µg/d	nein	Erwachsene Tumorpatienten, nach Chemotherapie		Keine negativen Effekte
Atzpodien J et al., Cancer Biother Radiopharm 2007 [58]	(+) GM-CSF		nein	Melanom-Patienten (Stadium IIA-IV)		Keine negativen Effekte
Barrio MM et al., J Immunther 2006 [59]	(+) GM-CSF	(+) 150 µg/d (I) 300 µg/d (II) 400 µg/d (III) 600 µg/d (IV)	ja (V)	Melanom-Patienten (Stadium IIB-IV)		AEs **(IV)**: Thoraxschmerz (3/4), abdominelle Krämpfe (2/4) Sonst gute Verträglichkeit
Bilgin K et al., Pediatrics 2001 [60]	GM-CSF	5 µg/kgKG/d (I) Kontrollgruppe (II)	nein	Neugeborene mit einer Sepsis-assoziierten Neutropenie Alter 33 (28-41) Wochen (I) / 33 (28-42) Wochen (II)	30 (I)	Senkung der Mortalität bei septischen, neutropenischen Neugeborenen
Bruno S et al., Cytokine 2006 [61]	G-CSF (I) + GM-CSF (II)	2 x 5 µg/kgKG/d (I) 2,5 µg/kgKG/d (II)	nein	Patienten mit akutem Myokardinfarkt (24 h nach PTCA) *1., 2. und 3. Tag (I) / 2. 4. und 6. Tag (II)*		Keine negativen Effekte
Bunn PAJ et al., J Clin Oncol 1995 [62]	(+) GM-CSF		nein	Patienten mit kleinzelligem Lungenkarzinom	230	Anwendung in Kombination mit Radio- und Chemotherapie sollte nicht angewendet werden SAE: Thrombozytopenie
Connor RF et al., Leuk Res 2006 [63]	GM-CSF (+ Imatinib)	100 µg/m²/d	nein	Chronisch myeloische Leukämie	CR	Keine negativen Effekte

Quelle	IP	Dosierung	Placebo	Population	N	Ergebnisse
Deng Z et al., Int J Cardiol 2006 [64]	GM-CSF	10 µg/kgKG/d (I)	ja (II)	Erwachsene Patienten mit akutem Myokardinfarkt<br>*Dauer 7 Tage*	10 (I)<br>10 (I)	Keine negativen Effekte<br>$CD34^+$-Zellen ↑
Drossou-Agakidou V et al., Cytokine 2002 [26]	GM-CSF (I)<br>G-CSF (II)	5 µg/kgKG/d (I)<br>10 µg/kgKG/d (II)	ja (III)	Septische Neugeborene<br>3 Tage (I), 4 Tage (II), 4 Tage (III)<br>Alter 32 Tage (I) / 33 Tage (II) / 32 Tage (III)	20 (I)<br>20 (II)<br>20 (III)	Keine negative Effekte<br>HLA-DR ↑
Hasskamp JH et al., J Clin Immunol 2006 [65]	GM-CSF (I)<br>(+ IL-2 (II))	125 µg/m²/d (I)	nein	Melanom-Patienten (Stadium IIb-IV)<br>*Postoperativ für 14 Tage gefolgt von II für 4 Tage; Wiederholung im monatlichen Rhythmus*		Keine negativen Effekte<br>Anti-Tumor-Potential
Heard SO et al., Crit Care Med 1998 [55]	G-CSF	75 µg/d (I)<br>300µg/d (II)	ja (III)	Patienten mit Schädel-Hirn-Trauma oder zerebraler Hämorrhagie<br>*(für 10 Tage / Neutrophile > 75.000/mm³ / Extubation)*<br>Alter 41(17-78) (I) /41(18-85)/45(20-81) (II)	21 (I)<br>20 (II)<br>20 (III)	In beiden GCS-Gruppen keine negativen Effekte<br>Risikoreduktion primärer Bakteriämien
Hueman MT et al., Breast Cancer Res Treat 2006 [66]	(+) GM-CSF		nein	Patienten mit Mamma-Karzinom (I) + Kontrollgruppe (II)	22 (I)<br>22 (II)	Keine negativen Effekte<br>T_{Reg} ↓, TGF-ß ↓
Hueman MT et al., Cancer Immunol Immunother 2007 [67]	(+) GM-CSF		nein	Patienten mit Mamma-Karzinom (I) + Kontrollgruppe (II)	22 (I)<br>17 (II)	Keine negativen Effekte<br>Aktivierung $CD4^+$- und $CD8^{+-}$T-Zellen
Kirman I et al., Eur J Surg Oncol 2007 [68]	GM-CSF (I)	125 µg/m²/d (I)	ja (II)	Patienten mit einem kolorektalen Karzinom (minimal invasive Resektion)<br>*Perioperativ (3., 2. und 1. Tag vor OP sowie am 1., 2., 3. und 4 Tag nach OP)*<br>Alter 59,1 (I) / 61 (II)	17 (I)<br>19 (II)	Keine negativen Effekte<br>HLA-DR ↑<br>sVEGFR1 ↑
Korzenik JR et al., N	GM-CSF	6 µg/kgKG (I)	ja (II)	Patienten mit Morbus Crohn	81 (I)	Keine negativen Effekte

Quelle	IP	Dosierung	Placebo	Population	N	Ergebnisse
Engl J Med 2005 [69]	(I)			*Behandlungsdauer 56 Tage*<br>Alter* 36 (20-72) (I) / 41 (21-74)	43 (II)	Reduktion des Schweregrades der Erkrankung, Erhöhung der Lebensqualität
Liljefors M et al., Cancer Immunol Immunother 2007 [70]	GM-CSF	200-250 µg/m²/d (I)<br>65-325 µg/m²/d (II)	nein	Patienten mit metastasiertem kolorektalen Karzinom<br>*10 Tage/Monat für 4 Monate*	50	ADCC der PBMCs ↓ am 10. Tag<br>Keine sonstigen negativen Effekte
McAleese JJ et al., Br J Radiol 2006 [71]	GM-CSF	2 µg/kgKG/d (I)<br>+<br>Kontrollgruppe	nein	Patienten mit Larynxkarzinom<br>*Behandlung für 14 Tage, 2 Wochen nach Bestrahlung*	29	AEs: Influenza-Symptome, 1 allergische Reaktion<br>Reduktion des Schweregra-des der Mukositis
Meisel C et al., noch nicht veröffentlichte Daten	GM-CSF (I)		ja (II)	Septische Patienten<br>Alter 66 (47-80) (I) / 68 (38-84) (II)		Keine negativen Effekte<br>HLA-DR ↑<br>TNF-α ↑
Mels AK et al., Br J Surg 2001 [72]	GM-CSF	8 µg/kgKG (I)	ja (II)	Patienten mit einem kolorektalen Karzinom<br>*Perioperativ (3 Tage vor OP bis 4 Tage nach OP)*	8 (I)<br>8 (I)	AE: leichte muskuloskeletale Schmerzen<br>HLA-DR ↑
Meropol NJ et al., Am J Surg 1996 [48]	GM-CSF	125 µg/m²/d (I)<br>250 µg/m²/d (II)	nein	Tumor-Patienten<br>*perioperativ (5 Tage vor OP und 5 Tage danach)*<br>Alter 61 (31 – 78)	12 (I)<br>11 (II)	Keine lebensbedrohlichen Ereignisse.<br>*Leukozytose (Median): 20.800/µl (125µg/m²) 29.300/µl (250µg/m²)*
Meropol NJ et al., J Clin Oncol 1998 [73]	GM-CSF	125 µg/m²/d (I)	ja (II)	Tumor Patienten<br>*perioperativ (3 Tage vor OP und 5 Tage danach)*<br>Alter 60,5 (22-86) (I) / 60,0 (21-85) (II)	198<br>201	GM-CSF wurde gut toleriert, Ø Fieber<br>Leukozytose: Median 10.900/µl
Nierhaus A et al., Intensive Care Med 2003 [22]	GM-CSF	5 µg/kgKG/d	nein	Intensivpatienten mit schwerer Sepsis und einer Immundepression (HLA-DR ↓)	9	Keine negativen Effekte<br>HLA-DR ↑
Oosterling SJ et al.,	GM-CSF	2,8 µg/kgKG/d	ja (II)	Patienten mit kolorektalem	10 (I)	Keine negativen Effekte

Quelle	IP	Dosierung	Placebo	Population	N	Ergebnisse
Immunobiology 2006 [74]		(I)		Karzinom<br>*Perioperative Behandlung (täglich vom 3. Tag vor OP bis 4 Tage nach OP)*<br>Alter 53 (I) / 64 (II)	8 (II)	
Orozco H et al., Arch Surg 2006 [75]	GM-CSF (I)	3 µg/kgKG/d (I)	ja (II)	Patienten mit abdomineller Sepsis<br>*Behandlungsdauer 4 Tage*<br>Alter 43 (I) / 49 (II)	28 (I)<br>30 (II)	Komplikationen im Rahmen der Infektionen ↓, Hospitalisierungsdauer ↓, Kosten ↓
Petilla V et al., Crit Care Med 2000 [76]	G-CSF	300 µg/d (I)	ja (II)	Beatmete Intensivpatienten (Posttraumatisch / postoperativ)<br>Alter 52.7 (28–73)/ 45.6 (20–76)	30 (I)<br>29 (II)	Keine negativen Effekte<br>Infektionen(↓):23,3%/ 37,9%, p=0,266
Powell A et al., Lung Cancer 2006 [77]	(+) GM-CSF		nein	Patienten mit einem malignem Mesotheliom		Keine negativen Effekte
Presneill JJ et al., Am J Respir Crit Care Med 2002 [78]	GM-CSF	3 µg/kgKG/d (I)	ja (II)	Patienten mit schwerer Sepsis und respiratorischer Dysfunktion<br>*Behandlungsdauer 5 Tage*<br>Alter 62 (32-72) (I) / 46,5 (37-60) (II)	10 (I)<br>8(II)	AE: Transiente reversible Oligurie bei einem Patienten mit Sepsis-assoziierter Niereninsuffizienz<br>Sonst keine negativen Effekte
Quittet P et al., Bone Marrow Transplant 2006 [79]	GM-CSF + G-CSF	5 x 2 µg/kgKG/d		Leukämie	60	Keine negativen Effekte
Rosenbloom AJ et al., Chest 2005 [23]	GM-CSF	125 µg/m² (3µg/kgKG/d) (I)	ja (II)	Intensivmedizinische Patienten mit Sepsis<br>Alter 56 (I) / 52 (II)	18 (I)<br>15 (I)	Keine negativen Effekte<br>Verbesserter Genesungsprozess von der Infektion
Schaefer H et al., Ann Surg 2004 [50]	G-CSF	300 µg/d (< 75 kgKG)<br>480µg/d (≥ 75 kgKG) (I)	ja (II)	Patienten mit Ösophagus-Ca (Ösophagusresektion)<br>*perioperativ (2 Tage vor OP und 7 Tage lang nach OP)*<br>Alter 58,7 (29-76) (I) / 60,2 (38-74) (II)	77 (I)<br>76(II)	Keine negative Effekte<br>AE: Leukozytose<br>Postoperative Infektionsrate nicht signifikant unterschiedlich zwischen

Quelle	IP	Dosierung	Placebo	Population	N	Ergebnisse
						den Gruppen
Schafer H et al., Ann Hematol 2000 [51]	G-CSF	300 µg/d (< 75 kgKG) 480µg/d (≥ 75 kgKG)	nein	Patienten mit Ösophagus-Ca (Ösophagusresektion) *perioperativ (2 Tage vor OP und 7 Tage lang nach OP)* Alter 60 (49-75)	19	Keine negativen Effekte Stimulation der Phagozytosefähigkeit
Schneider C et al., Ann Surg 2004 [20]	G-CSF	15 µgkgKG in 6 Tagen 5/5/5 (IA) 5/2/2/2/2/2 (IB)	ja	Allgemeinchirurgische Patienten *Perioperativ (2 Tage vor OP bis 3 Tage nach OP)* Alter 66 (63-69) (I) / 61 (59-63) (II)	40 (I) 20 (II)	Keine negativen Effekte Reduktion postoperativer Infektionen (13% (I) / 30 (II)
Schwaab T et al., Prostate 2006 [80]	GM-CSF	125 µg/m²/d (I) 250 µg/m²/d (II)	nein	Patienten mit Prostata-Karzinom 3 x/Woche		Keine negativen Effekte
Somani J et al., Vaccine 2002 [81]	(+) GM-CSF	250 µg (I)	ja (II)	Gesunde Probanden Alter 32 (I) / 30 (II)	15 (I) 15 (II)	Keine negativen Effekte
Vuylsteke RJ et al., Clin Cancer Res 2006 [82]	GM-CSF (I)		ja (II)	Melanom-Patienten	12	Keine negativen Ereignisse
Weiss M et al., Cytokine 1996 [83]	G-CSF	1 µg/kgKG/d *(erste 3 Tage)* 0,5 µ/kgKG/d *(weiter 5 Tage)*	nein	Postoperative/Posttrauma Patienten mit einem TISS-Score > 30	20	Keine negative Ereignisse
Weiss M et al., Intensive Care Med 2003 [53]	G-CSF	1µg/kgKG/d (I)	nein Kontroll-Gruppe (II)	SIRS-Patienten einer chirurgischen Intensivstation (posttraumatisch / -operative) Alter 64 (28–74) (I) / 63,5 (19–75) (II)	30 (I) 40 (II)	Keine schwerwiegenden Ereignisse AEs: Knochenschmerzen, temporärer Hautausschlag, Neutrophile (↑), CD 64 (↑)
Winter JN et al., J Clin Oncol 1996 [47]	G-CSF (I) + GM-CSF (II)	5 µg/kgKG/d (I) 5 µg/kgKG/d (II)	nein	Patienten mit Leukämie		Keine schwerwiegenden Erkrankungen

Bezüglich ergänzender Informationen zum Prüfpräparat Leukine® sei insbesondere auf die entsprechende *Investigator's Brochure (IB)* verwiesen, welche diesem Antrag beigefügt ist.

2.2.3 Isotonische Kochsalzlösung (Braun)

Pharmakodynamische Eigenschaften

Natrium und Chlorid sind die wichtigsten Elektrolyte im extrazellulären Raum und beeinflussen die Osmolarität und Flüssigkeitsverteilung entscheidend. Die Physiologische Kochsalzlösung "Fresenius" enthält Natrium und Chlorid in annähernd isotoner Konzentration.
Da bei der Infusion von isotoner Kochsalzlösung der Extrazellulärraum vergrößert wird, ist die Lösung zum initialen Volumenersatz geeignet. Aufgrund der guten Mischbarkeit sind isotone Kochsalzlösungen außerdem häufig verwendete Trägerlösungen.

Pharmakokinetik

Kochsalz ist ein im menschlichen Organismus natürlich vorkommender Bestandteil. Es wird unter Normalbedingungen in einer Menge von 8 - 15 g/d aufgenommen. Bei normaler Plasmakonzentration enthalten die 180 Liter, die in den Nieren täglich filtriert werden, ca. 1,5 kg NaCl. Davon werden normalerweise etwa 99 % rückresorbiert und weniger als 1 % ausgeschieden. Das genaue Ausmaß der Natriumausscheidung gleicht die Niere der Salzaufnahme so an, dass die Natriumkonzentration und damit das extrazelluläre Volumen im Körper konstant gehalten werden.

Anwendungsgebiete

Natrium- und Chloridverlust, geringgradige hypochlorämische Alkalose, kurzzeitige Volumensubstitution in Notfällen, Vehikel- und Spüllösung, isotone und hypotone Dehydratation wenn eine Kontraindikation für kaliumhaltige Lösungen besteht.

Präklinische Daten zur Sicherheit

Es kann davon ausgegangen werden, dass Physiologische Kochsalzlösung "Fresenius" per se untoxisch ist, abgesehen von der bei Gabe großer Mengen oder bei Schnellinfusion auftretenden Volumentoxizität.

2.3 Begründung der Behandlungs- und Untersuchungsverfahren

Bei vorgenannter Studie, handelt es sich um eine randomisierte (Blockbildung) und verblindete Studie. Diese Maßnahmen im Studiendesign werden damit begründet, die Aussagekraft der erhobenen Daten zu erhöhen.

Durch die Anwendung oben genannter Prüfpräparate (MUTAGRIP® oder LEUKINE®) soll eine Immunstimulation in der Phase der postoperativen Immundepression erzielt werden, die damit eine Immunparalyse verhindert und so zu einer signifikanten Reduktion postoperativer Komplikationen führt. Dabei fungiert die isotonische Kochsalzlösung als Kontrolle zu den anderen beiden Prüfpräparaten. Hinsichtlich der Behandlung postoperativer Immundepression existiert keine Standardtherapie. Somit kann z. Z. kein Nachteil für jene Patienten festgestellt werden, welche mit dem Placebo behandelt werden. Die Messung eines möglichen Vorteils für die Behandlung mit einem der Prüfpräparate ist ja gerade Gegenstand dieser Untersuchung.
Die Behandlung mit den Prüfpräparaten erfolgt als Add-on-Therapie. Allen Patienten ist zu jedem Zeitpunkt der Studienteilnahme die Behandlung, nach den Standards der Universitätsklinik für Anästhesiologie mit Schwerpunkt operative Intensivmedizin, Charité – Universitätsmedizin Berlin, garantiert.

3 Ziele der klinischen Prüfung

Eine postoperative Immunstimulation durch Vakzinierung mit Influenza-Impfstoff oder Gabe von Sargramostim im Stadium der klinisch unauffälligen Immundepression, könnte die Immunreaktivität verbessern und mit einer verminderten postoperativen Infektionsinzidenz einhergehen. Auf diese Weise könnte möglicherweise die postoperative Morbidität und Mortalität der Patienten gesenkt werden.

3.1 Primärer Endpunkt

Primärhypothese:
Eine postoperative Vakzinierung mit Influenza-Impfstoff oder eine postoperative Behandlung mit Sargramostim im Stadium der schweren Immundepression führt, im Vergleich zu Placebo, zu einer Verbesserung der Immunreaktivität bei Ausbleiben einer Immunparalyse, gemessen anhand der Normalisierung der monozytären HLA-DR Expression.

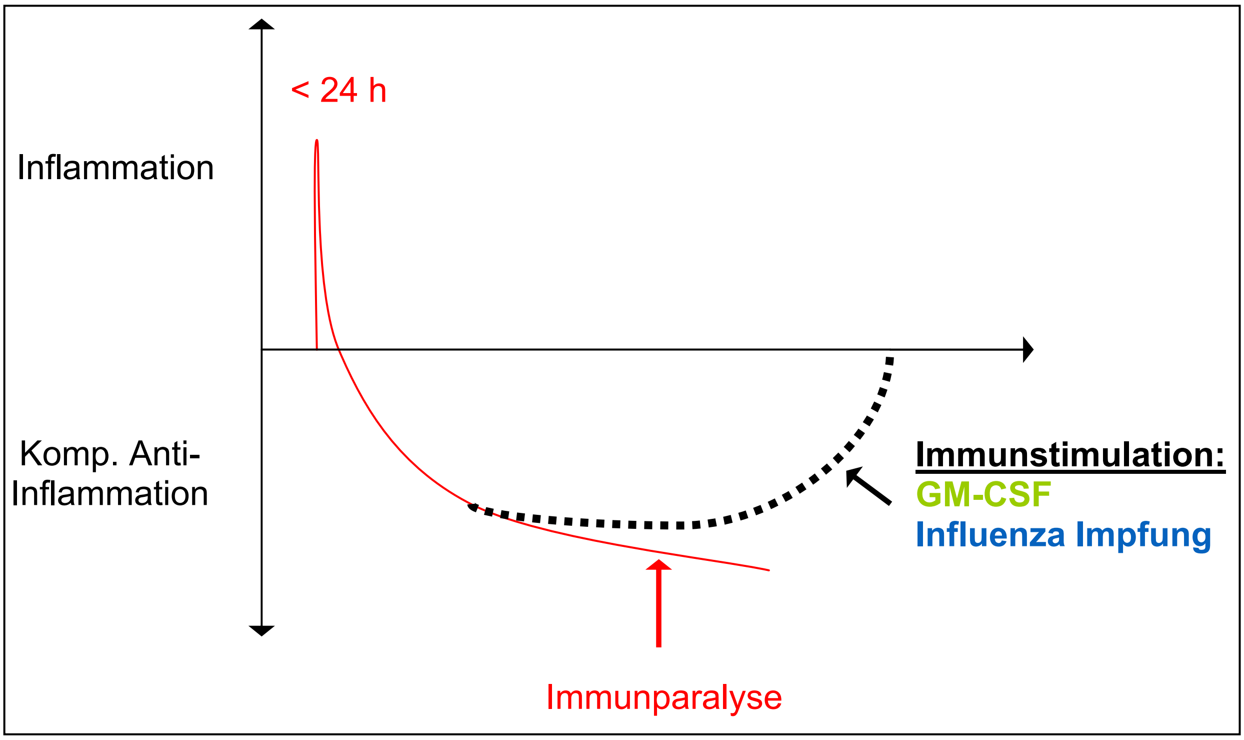

Immunstimulation durch Influenza-Impfung/GM-CSF in der Phase der Immundeppresion; Verhinderung der Immunparalyse

3.2 Sekundäre Endpunkte

- Postoperative Infektionsrate
- Postoperative Delirrate (CAM-ICU, NuDESC, DDS)
- Beatmungsstunden, ITS-Liegedauer, Charité-Liegedauer, APACHE-II-Score, SAPS-II-Score, SOFA-Score, TISS-28-Score
- Die ersten 33 eingeschlossenen Patienten der Behandlungsgruppen:

1. Zytokinproduktion im Serum, das Th_1/Th_2-Verhältniss, das Th_{17}/T_{reg}-Verhältnis, andere Immunfunktionsparameter
2. Quantitative Expression von Transkriptionsfaktoren (T-bet, Eomesodermin, GATA-3, Foxp3, RORγt, PU.1) und Proteinen (SOCS-3, SOCS-1, TGF-ß, IL-17) sowie die Synthese der entsprechenden Effektorproteine

3.3 Studiendesign

Bei der Studie handelt es sich um eine monozentrische, prospektive, kontrollierte, randomisierte, doppelblinde, placebokontrollierte Pilotstudie im Double Dummy Design.
Patienten, welche sich einer elektiven Ösophagus- oder Pankreasresektion unterziehen, werden nach ihrer Aufnahme in der Charité – Universitätsmedizin Berlin, Campus Virchow Klinikum, von dem Prüfarzt der Universitätsklinik für Anästhesiologie mit Schwerpunkt operative Intensivmedizin, Campus Virchow Klinikum, im Anschluss an das Narkoseaufklärungsgespräch auf die geplante Studie aufmerksam gemacht sowie über den Inhalt der Studie und mögliche Risiken mündlich als auch schriftlich aufgeklärt (Run-In).
Im Rahmen des „Screenings“ erfolgt am 1. postoperativen Tag u.a. die Bestimmung der HLA-DR-Expression auf Monozyten. Bei einem Wert von ≤ 10.000 Antigenen/Monozyt wird der Patient durch Randomisierung einem der drei Studienarme zugeordnet. Dabei erfolgt die Gabe der jeweiligen Studienmedikation doppelblind als Add-on-Therapie.

1. Influenza-Impfstoff (MUTAGRIP® 2008/2009, Sanofi Pasteur MSD GmbH) + NaCl 0,9% (Braun)
2. Sargramostim (GM-CSF, Leukine®, Bayer Health Care ™) + NaCl 0,9% (Braun)
3. NaCl 0,9% (Braun)

Ergibt die Bestimmung der HLA-DR-Expression auf Monozyten am 1. postoperativen Tag einen Wert von > 10.000 Antigenen/Monozyt erfolgt kein Studieneinschluss für diesen Patienten. () Insgesamt werden 60 Patienten in die Studie eingeschlossen. Jedem Studienarm werden 20 Patienten zugeteilt.

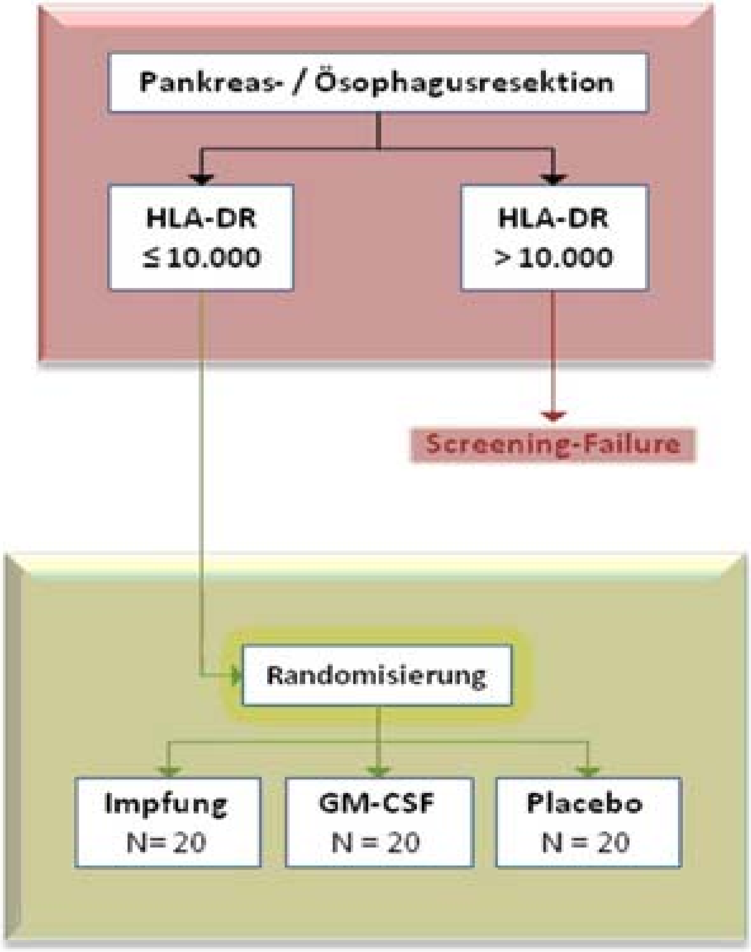

„Screening-Phase" **„Studieneinschluss"**

In diesem Zusammenhang wird auch auf die Kapitel 5.4.1 (Behandlungsschema) und 6 (Studienablauf) verwiesen.

()

3.4 Zeitplan

Beginn der Studie

- Zeitpunkt der Aktivierung der Studie: 26.10.2008
- geplante Dauer der Rekrutierungsphase: 2 Jahre und 5 Monate

Ende der Studie

- voraussichtlicher Zeitpunkt des Studienendes: 31.03.2011
- geschätzte Dauer der Auswertung bis zur Vorlage des Berichts: 12 Monate

Bedingungen für den Einschluss des letzten Patienten:

- Ein- und Ausschlusskriterien sind erfüllt.
- Die HLA-DR-Expression auf Monozyten am 1. postoperativen Tag ist ≤ 10.000 Antigene/Monozyt.
- Sechzigster Patient, welcher in einen der drei Studienarme randomisiert wurde und die entsprechende Studienmedikation erhält.

Die Studie endet für den einzelnen Patienten am 9. postoperativen Tag oder bei vorheriger Entlassung / Verlegung in eine Klinik außerhalb der Charité – Universitätsmedizin Berlin sowie bei vorzeitigem Studienabbruch (siehe auch Kapitel 8.1 und 8.2). Ein generelles „Follow-Up“ wird nicht durchgeführt. Mit der Abschlussuntersuchung am 9. postoperativen Tag noch nicht abgeschlossene AEs werden im Rahmen eines „Follow-Up“ von 7 Tagen, noch nicht abgeschlossene SAEs im Rahmen einer „Follow-Up von 21 Tagen, nachverfolgt (siehe auch Kapitel 1 und 9.4).

4 Auswahl der Patienten

4.1 Einschlusskriterien

- Elektive Pankreasresektion oder Ösophagusresektion
- Alter ≥ 18 Jahre
- Durchgeführte Patientenaufklärung
- Schriftliche Einwilligung (laut AMG §40 (1) 3b)
- negativer Schwangerschaftstest im Rahmen des Screenings (βHCG im Urin)
- hocheffektive Kontrazeption bei Frauen (definiert als Pearl Index < 1) oder anamnestisch mehr als 2 Jahre postmenopausal
- keine Teilnahme an einer anderen Studie nach dem AMG einen Monat vor Studieneinschluss sowie während der gesamten Studienteilnahme
- HLA-DR-Expression auf Monozyten ≤ 10.000 Antigene/Monozyt am 1. postoperativen Tag (BE2)

4.2 Ausschlusskriterien

- Fehlende Einverständniserklärung
- Alter < 18 Jahre
- fehlende Bereitschaft zur Speicherung und Weitergabe pseudonymisierter Krankheitsdaten im Rahmen der klinischen Prüfung
- Unterbringung in einer Anstalt auf gerichtliche oder behördliche Anordnung (laut AMG §40 (1) 4)
- Teilnahme an einer anderen Studie nach dem AMG
- Mitarbeiter der Charité
- Bekannte Schwangerschaft oder positiver Schwangerschaftstest (βHCG im Urin) im Rahmen des Screenings
- Stillzeit
- Frauen im gebärfähigen Alter ohne gesicherte Kontrazeption
- Angeborene oder erworbene Bluterkrankung
- Chemo- oder Radiotherapie innerhalb der letzten 28 Tage
- Leukämie
- Notfalloperationen
- Nachgewiesene Infektion in den letzten 7 Tagen vor der geplanten Operation
- Bekannte Hepatitis B oder C Infektion oder positiver Labortest im Rahmen des Screenings
- Bekannte HIV-Infektion oder positiver Labortest im Rahmen des Screenings
- Bekannte Allergien/Überempfindlichkeiten auf einen der arzneilich wirksamen oder sonstigen Bestandteile bzw. auf einen der möglichen Produktionsrückstände der genannten Prüfpräparate.
- Autoimmunerkrankungen
- Einnahme von Immunsuppressiva bis zu vier Wochen vor Studieneinschluss
- Nicht therapierte Herzrhythmusstörungen
- Instabile Angina pectoris

- Symptomatische angeborene Herzfehler
- Thrombosen oder thrombembolische Ereignisse in der klinischen Vorgeschichte
- Körpergewicht < 50 Kilogramm
- HLA-DR-Expression auf Monozyten > 10.000 Antigene/Monozyt am 1. postoperativen Tag (BE2)
- Labor (am Tag vor der Operation BE0):

Thrombozyten	≤ 100.000/µl
Neutrophile	≤ 1.500/µl
Hämoglobin	≤ 8g/dl
Bilirubin	> 2g/dl
Kreatinin	> 1,5g/dl
ASAT/ALAT	> 90U/l

Alle Kontraindikationen und Wechselwirkungen der entsprechenden Prüfpräparate wurden bei der Festlegung der Ein- und Ausschlusskriterien mitberücksichtigt und entsprechend inkludiert.

Es wird keine besondere Geschlechterverteilung berücksichtigt, da keine geschlechtsspezifischen Unterschiede bei der Wirksamkeit oder Verträglichkeit des zu prüfenden Arzneimittels zu erwarten sind (Vgl. GCP-V § 7 (2) Nr. 12).

5 Behandlungsplan

Im Rahmen dieser Pilotstudie kommen folgende Prüfpräparate zur Anwendung:
1. Influenza-Impfstoff (MUTAGRIP® 2008/2009, Sanofi Pasteur MSD GmbH)
2. Sargramostim (GM-CSF, Leukine®, Bayer Health Care ™)
3. Isotonische Kochsalzlösung (Braun)

5.1 Beschreibung der Prüfmedikation MUTAGRIP® *(2007/2008)** (Sanofi Pasteur MSD GmbH)

Im Folgenden wird die Information aus der Fachinformation für MUTAGRIP® 2009/2010 beispielhaft aufgelistet.

Allgemein

Inaktivierter trivalenter Grippespaltimpfstoff, konservierungsmittelfrei.
Suspension zur Injektion.

Wirkstoffe, Zusammensetzung

Eine Impfdosis von 0,5 ml enthält je 15µg Hämagglutinin von Grippeviren* entsprechend der Stämme
A/Brisbane/59/2007 (H1N1) – entsprechender Stamm (A/Brisbane/59/2007 [IVR-148]) 15 Mikrogramm HA2 A/Brisbane/10/2007 (H3N2) – entsprechenderStamm (A/Uruguay/716/2007 [NYMC X-175C]) 15 Mikrogramm HA2 B/Brisbane/60/2008 – entsprechender Stamm (B/Brisbane/60/2008) 15 Mikrogramm HA2 1 gezüchtet in befruchteten Hühnereiern aus gesunden Hühnerbeständen 2 Hämagglutinin

sowie Pufferlösungen aus Natriumsalzen (Natriumchlorid, Dinatriumhydrogenphosphat-Dihydrat), Kaliumsalzen (Kaliumchlorid, Kaliumdihydrogenphosphat) und Wasser für Injektionszwecke, in Spuren Hühnereiproteine, Hühnerproteine, Neomycin, Formaldehyd und Octozinol 9.
Die Zusammensetzung entspricht den jeweils aktuellen Empfehlungen der WHO (für die nördliche Hemisphäre) und der Europäischen Gemeinschaft.

** Vermehrt in Hühnereiern.*

*** Da es nicht immer möglich ist, diese Viren in ausreichendem Maße zu kultivieren, oder die Viren noch nicht in Reinform gewonnen werden konnten, werden von der EMEA auch dem Wildtyp-ähnliche Reassortanten akzeptiert. Dabei handelt es sich um folgende:*

Darreichungsform und Inhalt des Behältnisses
Ein Behältnis enthält 0,5 ml Suspension in einer vorgefüllten Spritze (Glasart Typ I) mit aufgesetzter Kanüle, Kolbenstopfen aus Chlorobromobutyl-Elastomer.

Bereitstellung
Die Ausgabe des Prüfpräparates erfolgt durch die Klinikapotheke.

Lagerung und Aufbewahrungshinweis
MUTAGRIP® 2009/2010 muss vor Licht geschützt im Kühlschrank bei einer Temperatur zwischen +2° C und +8° C aufbewahrt werden. Eine Aufbewahrung über oder unter der empfohlenen Temperatur vermindert die Wirksamkeit des Impfstoffes. MUTAGRIP® 2009/2010 darf nicht eingefroren werden. Impfstoffe, die versehentlich falsch gelagert oder eingefroren wurden, sind zu verwerfen.

Warnhinweise und Vorsichtsmaßnahmen
Wie bei allen injizierbaren Impfstoffen kann es in seltenen Fällen zu einer bedrohlichen allergischen Reaktion nach der Impfung bis hin zum Schock kommen. Geeignete Mittel zur Akutbehandlung (Adrenalin, Kortikosteroide, Antihistaminika, Volumenauffüllung, Sauerstoff) sind jederzeit unmittelbar verfügbar. Eine angemessene Überwachung in diesen Fällen ist gewährleistet.
MUTAGRIP® 2009/2010 darf auf keinen Fall intravasal verabreicht werden.

Anmerkungen
Für weitere Informationen wird auf die Fachinformation sowie das „Vereinfachtes IMPD“ gemäß Anlage II/1 der 3. Bekanntmachung zur klinischen Prüfung von Arzneimittel am Menschen und „Detailed Guidance“ ENTR/F2/BL D (2003) CT 1, für den aktuellen Impfstoff MUTAGRIP® 2009/2010 verwiesen.

5.1.1 Verzeichnis der Nebenwirkungen und Wechselwirkungen

Die Chargenfreigabe und zeitgleiche Genehmigung der Änderungsanzeige erfolgt durch das Paul-Ehrlich-Institut (PEI). Dabei erfolgt die Sicherheitsbeurteilung des Impfstoffes insbesondere auf Grundlage des „Periodic Safety Update Reports“ (PSUR) als Maß für die Pharmakovigilanz.
Darüber hinaus erfolgt im Rahmen der jährlichen Aktualisierung der Stammzusammensetzung die Durchführung offener Studien ohne Kontrollgruppe durch Sanofi-Aventis.
Desweiteren informiert die Datenbank des PEI detailliert über Verdachtsfälle auf Impfkomplikationen sowie über Verdachtsfälle schwerwiegender Nebenwirkungen nach Impfungen bei denen in unterschiedlichem zeitlichem Abstand nach einer oder mehrerer Impfungen ein bleibender Gesundheitsschaden berichtet worden ist oder Patient verstorben ist. Die Datenbank erlaubt es gezielt nach bestimmte Reaktionen oder Impfstoffen zu recherchieren [84].
Weiter Ausführungen hierzu siehe auch Kapitel 2.2.1 in diesem Antrag sowie in den Ausführungen des vereinfachten IMPD.

Beobachtete Nebenwirkungen werden mit folgenden Häufigkeitsangaben gelistet:

sehr häufig	-	>10%
Häufig	Lokale Reaktionen: Rötungen, Schwellungen, Schmerzen, Ekchymose (kleinflächige Hautblutung) und Verhärtungen Allgemeine Unverträglichkeitsreaktionen: Fieber, Unwohlsein, Schüttelfrost, Müdigkeit, Kopfschmerzen, Schwitzen, Muskel- und Gelenkschmerzen.<br>Diese Reaktionen verschwinden gewöhnlich nach 1 -2Tagen ohne Behandlung.	>1% und <10%
gelegentlich*	Allgemeine Hautreaktionen einschließlich Pruritus (Juckreiz), Urtikaria (Nesselsucht) oder nicht näher spezifizierte Hautausschläge.	>0,1% und <1%
selten*	Neuralgien (Nervenschmerzen), Parästhesien (Missempfindungen), Krämpfe, vorübergehende Thrombozytopenie (Verminderung der Anzahl der Blutplättchen). Allergische Reaktionen:<br>in seltenen Fällen bis hin zum Schock Angioödem (allergische Schwellungen im Bereich des Gesichts und Halses) in sehr seltenen Fällen	>0,01% und <0,1%
sehr selten*	Vaskulitis (Gefäßentzündung) mit vorübergehender Beteiligung der Nieren. Neurologische Erkrankungen wie Enzephalomyelitis (Entzündung des Gehirns und Rückenmarks), Neuritis (Nervenentzündung) und Guillain-Barre-Syndrom (aufsteigende Lähmung).	<0,01%, einschl. Einzelfälle

*** Diese Nebenwirkungen wurden zusätzlich während der Post-Marketing-Überwachung beobachtet.*

Zwecks weiterer detaillierter Ausführungen zu Verdachtsfällen auf Impfkomplikationen sowie über Verdachtsfälle schwerwiegender Nebenwirkungen nach Impfung sei auf die Ausführungen zur Datenbank des Paul-Ehrlich-Instituts im beiliegenden vereinfachten IMPD verwiesen.

5.2 Beschreibung der Prüfmedikation Sargramostim (Leukine®, Bayer Health Care™)

Allgemein

Sargramostim (Leukine®) ist ein mittels rekombinanter DNA-Technologie aus Hefekulturen (S. cervisiae) hergestellter humaner Granulozyten-Makrophagen Kolonie-stimulierender Faktor. Es handelt sich dabei um einen hämatopoetischen Wachstumsfaktor der die Proliferation und Differenzierung hämatopoetischer Vorläuferzellen stimuliert.
Desweiteren aktiviert Sargramostim voll differenzierte Granulozyten, Monozyten/Makrophagen und dendritische Zellen. Sargramostim wirkt wie endogenes humanes GM-CSF durch Bindung an spezifische Rezeptoren, die von den Zielzellen auf der Zelloberfläche exprimiert werden.

Wirkstoffe und Zusammensetzung

Eine Ampulle Leukine® enthält

- 250 µg (1,4 x 10^6 IU) Sargramostim in **lyophilisierter, pulverisierter Form** (Rekombinanter humaner Granulozyten-Makrophagen Kolonie-stimulierender Faktor GM-CSF),
- 40 mg/ml Mannitol
- 10 mg/ml Saccharose und
- 1,2 mg/ml Tromethamin.

Darreichungsform und Inhalt des Behältnisses
Eine Ampulle lyophilisiertes LEUKINE® enthält 250 µg Sargramostim.

Bereitstellung
Die Ausgabe des Prüfpräparates erfolgt durch die Klinikapotheke.

Lagerung und Aufbewahrungshinweis
Leukine® sollte bei 2° C bis 8° C im Kühlschrank gelagert werden. Die Ampullen sollten weder gefroren noch geschüttelt werden. Impfstoffe, die versehentlich falsch gelagert oder eingefroren wurden, sind zu verwerfen.

Warnhinweise und Vorsichtsmaßnahmen
Auf Grund der potenziellen Empfindlichkeit, auf sich rasch teilende, hämatopoetische Vorläuferzellen, sollte Sargramostim nicht bei gleichzeitig zytotoxischer Chemo- oder Strahlentherapie verabreicht werden oder innerhalb von 24 Stunden nach einer Chemo- oder Strahlentherapie angesetzt werden.
Leukine® sollte nur unter Aufsicht eines Mediziners benutzt werden, der in der Behandlung von onkologischen und hämatopoetischen Erkrankungen oder von Infektionskrankheiten erfahrenen ist. Die Erstdosis von Leukine® sollte unter enger medizinischer Überwachung verabreicht werden.
Akute schwere, lebensbedrohliche Hypersensitivitätsreaktion, einschließlich Anaphylaxie, Angioödem und Bronchokonstriktion sind bei der Einnahme von Leukine® aufgetreten. Wenn solche Reaktionen auftreten, sollte Leukine® sofort abgesetzt werden und darf nicht wieder angesetzt werden.
Leukine® wurde selten mit Pleuritis, Pleuraerguß, Perikarditis- und/oder Perikarderguß assoziiert. Sollten solche Reaktionen auftreten, sollte Leukine® abgesetzt werden.
Patienten mit vorbestehender Lungenkrankheit können durch eine vermindernder Lungenfunktion und Dyspnoe unter der Gabe von Leukine® auffällig werden und sollte engmaschig überwacht werden.

5.2.1 Verzeichnis der Nebenwirkungen und Wechselwirkungen

Wasserretention, Inzidenz: Leukine® vs. Placebo

- Periphere Ödeme	11% vs. 7%
- Pleuraerguß	1% vs. 0%
- Perikarderguß	4% vs. 1%
- Capillary-leek-Syndrom	< 1% (in vereinzelten Studien)

Respiratorische Syndrome
Dyspnoe, insb. unmittelbar nach erstmaliger Leukine®-Infusion

Kardiovaskuläre Symptome

Supraventrikuläre Arrhythmien

Renale und hepatische Dysfunktionen

Vereinzelt wurde bei Patienten mit einem renalen oder hepatischen Grundleiden nach Verabreichung von Leukine® eine Erhöhung Serum-Kreatinins, Bilirubins oder anderer hepatischer Enzyme beobachtet. Nach Dosisreduktion bzw. Absetzen von Leukine® waren diese Werte wieder rückläufig und erreichten in kürzester Zeit Ausgangswerte. In Placebo-kontrollierten Studien traten entsprechende Nebenwirkungen jedoch mit gleicher Häufigkeit auf.

5.3 Isotonische Kochsalzlösung (Braun)

Zusammensetzung

100 ml Injektionslösung enthalten 0,9 g Natriumchlorid

Na^+ 0,154 mmol/ml
Cl^- 0,154 mmol/ml

Hilfsstoffe: Wasser für Injektionszwecke, Salzsäure 25%, Natriumhydroxid.

Darreichungsform

Infusions- und Standardinjektionslösung; Trägerlösung für Medikamente.

Bereitstellung

Die Ausgabe des Prüfpräparates erfolgt durch die Klinikapotheke.

Lagerung und Aufbewahrungshinweis

Nicht über 25° C aufbewahren.

Warnhinweise und Vorsichtsmaßnahmen

In Abhängigkeit vom zugeführten Volumen und dem Zustand des Patienten können Kontrollen des Elektrolyt- und Flüssigkeitsstatus erforderlich sein.

5.3.1 Verzeichnis der Nebenwirkungen und Wechselwirkungen

Nebenwirkungen sind bei bestimmungsgemäßer Anwendung nicht bekannt. Zufuhr von zu großen Volumina von Isotonischer Kochsalzlösung kann zur Hypernatriämie und Hyperchlorämie führen.
Wechselwirkungen mit anderen Mitteln sind ebenfalls keine bekannt.

5.4 Prüfpräparate und ihre Zubereitung

5.4.1 Behandlungsschema

Geplant sind pro Patient sieben Blutentnahmen (BE0 bis BE6), jeweils am Tage vor der Operation (BE0), am Morgen vor der Operation (BE1) sowie am Morgen des 1. bis 5. postoperativen Tages (BE2 – BE6). Im Rahmen der 3. Blutentnahme (BE2) wird die HLA-DR Expression auf Monozyten bestimmt. Ein Abfall der HLA-DR Expression auf Monozyten ≤ 10.000 Antigene/Monozyt am 1. postoperativen Tag wird als schwere Immundepression gewertet. Patienten welche diesen „Cut-off“ unterschreiten, erhalten randomisiert und doppelblind die entsprechenden Prüfpräparate. Die entsprechenden Studienmedikamente

werden jeweils am 1., 2. und 3. postoperativen Tag nach Blutentnahme durch den zuständigen Prüfarzt subkutan bzw. intravenös verabreicht.

vOP	OP	1. pOP	2. pOP	3. pOP	4. pOP	5. pOP	6. pOP	7. pOP	8. pOP	9. pOP
**E**										**AU**
**BE0**	**BE1**	**BE2**	**BE3**	**BE4**	**BE5**	**BE6**				↓
		**SM1**	**SM2**	**SM3**						
**nach E**	**6.30 Uhr**	**6.30 Uhr**	**6.30 Uhr**	**6.30 Uhr**	**6.30 Uhr**	**6.30 Uhr**				**SE**
		**nach LE**	**8.00 Uhr**	**8.00 Uhr**						

E = Patienteneinschluss; BE = Blutentnahme; SM = Studienmedikation; AU = Abschlussuntersuchung; SE = Studienende; vOP = Tag vor der Operation; OP = Operation; pOP = postoperativ; LE = Vorliegen des Laborergebnisses der HLA-DR-Messung
Abweichungen von bis zu 60 Minuten vom der hier angegebenen Zeitpunkten sind keine Protokollabweichungen!

5.4.2 Prüfpräparate

Nr.	Arzneimittel	Zusammensetzung
1.	Prüfarzneimittel »Verum 1«	0,5 ml Grippeimpfstoff<br>1 Spritze mit 0,5 ml Grippeimpfstoff<br>+<br>1 Perfusor®-Spritze mit 24 ml Infusionslösung:<br>Isotone Natriumchloridlösung 0,9%
2.	Prüfarzneimittel »Verum 2«	Sargramostim 250 µg/m2 KOF<br>1 Spritze mit 0,5 ml isotoner Natriumchlorid-lösung 0,9%<br>+<br>1 Perfusor®-Spritze mit 24 ml Infusionslösung:<br>250 µg/m2 KOF Sargramostim in isotoner Natriumchloridlösung 0,9%
3.	Prüfarzneimittel »Placebo«	Isotone Natriumchloridlösung 0,9%<br>1 Spritze mit 0,5 ml isotoner Natriumchlorid-lösung 0,9%<br>+<br>1 Perfusor®-Spritze mit 24 ml Infusionslösung:<br>Isotone Natriumchloridlösung 0,9%
4.	Darreichungsform	Spritze: Injektionslösung zur subkutanen Injektion<br>Perfusor®-Spritze: Infusionslösung zur intravenösen Infusion

5.4.3 Bezeichnung und Materialien

	**Bezeichnung**	**Materialien**
1.	Ausgangsstoffe	
	»Verum 1«	Spritze:<br>Grippeimpfstoff Mutagrip®<br>Fertigspritze mit 0,5 ml Lösung zur Injektion<br>Perfusor®-Spritze:<br>Isotonische Kochsalzlösung 0,9% 50 ml Injektionsflasche

	»Verum 2«	Spritze:<br>Isotone Natriumchloridlösung 0,9% Braun 10 ml Amp.<br>Perfusor®-Spritze:<br>Vial mit 250 µg Sargramostim Trockensubstanz<br>Lösungsmittel: Aqua ad iniect. Braun 10 ml Amp.<br>Isotonische Kochsalzlösung 0,9% 50 ml Injektionsflasche
	Placebo	Spritze:<br>Isotone Natriumchloridlösung 0,9% Braun 10 ml Amp.<br>Perfusor®-Spritze:<br>Isotonische Kochsalzlösung 0,9% 50 ml Injektionsflasche
2.	Lagerbedingungen	Grippeimpfstoff Mutagrip® und<br>Leukine®:<br>Im Kühlschrank zwischen 2 und 8°C<br>Isotone Natriumchloridlösung 0,9% und Wasser für Injektionszwecke: Keine besonderen Lagerbedingungen
3.	Behältnisse	Spritze:<br>Sterile Einmalspritze Luer 1,0 ml Transcoject ® CE 0482 (Spritzen ohne Totvolumen),<br>Kombi-Stopfen<br>Perfusor®-Spritze:<br>Original-Perfusor®-Spritze OPS 50 ml Luer Lock<br>CE 0123<br>Kombistopfen
4.	Weitere Materialien für die Zubereitung	Spritze:<br>Kanüle<br>Perfusor®-Spritze:<br>Entnahme-Spike<br>Nur bei »Verum 2«: 5 ml Einmal-Spritzen, Kanülen
5.	Umverpackung<br>»Sterilfolie Spritze«<br>»Sterilfolie Perfusor®-Spritze «	Spritze:<br>Sterilfolie Tüten 300 x 100 mm<br>Perfusor®-Spritze:<br>Sterilfolie Tüten 400 x 100 mm
6.	Verpackung Sets<br>»Sterilfolie Set«	3 Sets/Patient für die Tage 1-3 postOP:<br>1 Spritze, 1 Perfusor®-Spritze,<br>1 Kanüle Sterican 26G x 1/2“ Braun,<br>1 Perfusor®-Leitung
		Umverpackung: Sterilfolie Tüte 410 x 210 mm
7.	Etiketten	Zweckform DIN A4

Für weitergehende Informationen wird auf die *Anweisung zur Zubereitung vor der Anwendung* im Prüfarztordner verwiesen.

5.4.4 Volumenberechnung

Benötigtes Volumen an verdünnter, rekonstituierter Leukine®-Lösung bezogen auf die Körperoberfläche des Patienten unter Berücksichtigung von 3 ml Totraumvolumen für die Perfusor®-Spritze

KOF (m^2)	Volumen (ml)
1,40	7,9
1,41	7,9
1,42	8,0
1,43	8,0
1,44	8,1
1,45	8,2
1,46	8,2
1,47	8,3
1,48	8,3
1,49	8,4
1,50	8,4
1,51	8,5
1,52	8,6
1,53	8,6
1,54	8,7
1,55	8,7
1,56	8,8
1,57	8,8
1,58	8,9
1,59	8,9
1,60	9,0
1,61	9,1
1,62	9,1
1,63	9,2
1,64	9,2
1,65	9,3
1,66	9,3
1,67	9,4
1,68	9,5
1,69	9,5
1,70	9,6
1,71	9,6
1,72	9,7
1,73	9,7
1,74	9,8
1,75	9,8
1,76	9,9
1,77	10,0
1,78	10,0
1,79	10,1
1,80	10,1

KOF (m^2)	Volumen (ml)
1,81	10,2
1,82	10,2
1,83	10,3
1,84	10,4
1,85	10,4
1,86	10,5
1,87	10,5
1,88	10,6
1,89	10,6
1,90	10,7
1,91	10,7
1,92	10,8
1,93	10,9
1,94	10,9
1,95	11,0
1,96	11,0
1,97	11,1
1,98	11,1
1,99	11,2
2,00	11,3
2,01	11,3
2,02	11,4
2,03	11,4
2,04	11,5
2,05	11,5
2,06	11,6
2,07	11,6
2,08	11,7
2,09	11,8
2,10	11,8
2,11	11,9
2,12	11,9
2,13	12,0
2,14	12,0
2,15	12,1
2,16	12,2
2,17	12,2
2,18	12,3
2,19	12,3
2,20	12,4
2,21	12,4

KOF (m^2)	Volumen (ml)
2,22	12,5
2,23	12,5
2,24	12,6
2,25	12,7
2,26	12,7
2,27	12,8
2,28	12,8
2,29	12,9
2,30	12,9
2,31	13,0
2,32	13,1
2,33	13,1
2,34	13,2
2,35	13,2
2,36	13,3
2,37	13,3
2,38	13,4
2,39	13,4
2,40	13,5
2,41	13,6
2,42	13,6
2,43	13,7
2,44	13,7
2,45	13,8
2,46	13,8
2,47	13,9
2,48	14,0
2,49	14,0
2,50	14,1
2,51	14,1
2,52	14,2
2,53	14,2
2,54	14,3
2,55	14,3
2,56	14,4
2,57	14,5
2,58	14,5
2,59	14,6
2,60	14,6

Für weitergehende Informationen wird auf die *Anweisung zur Zubereitung vor der Anwendung* verwiesen.

5.4.5 Dosierung und Art der Anwendung der Prüfpräparate

Influenza-Impfstoff (MUTAGRIP® 2008/2009)<br>**und**<br>NaCl 0,9% (Braun)	1 x tgl. 0,5 ml s.c.<br><br>24 ml/24h (1ml/h Laufrate) i.v.
Sargramostim (GM-CSF, Leukine®)<br>**und**<br>NaCl 0,9% (Braun)	1 x tgl. 5µg/kgKG s.c.<br><br>1 x tgl. 0,5 ml s.c.
Isotonische Kochsalzlösung (Braun)<br>**und**<br>NaCl 0,9% (Braun)	1 x tgl. 0,5 ml s.c.<br><br>24 ml/24h (1ml/h Laufrate) i.v.

5.5 Kriterien für einen Behandlungsabbruch

5.5.1 Vorzeitiger Studienabbruch eines einzelnen Patienten

- Widerruf der Einwilligung
- schwere Krankheitserscheinungen (z.B. anaphylaktischer Schock, Perikarditis, schwere Krämpfe, Vaskulitis, Enzephalomyelitis, Guillain-Barre-Syndrom, etc.) insofern nicht eine andere Ursache (z.B. chirurgische, anästhesiologische oder diagnostische Prozeduren) erkannt wird und die Entblindung des verabreichten Studienmedikamentes notwendig ist oder eine ab diesem Zeitpunkt vorgesehene Gabe von Studienmedikamenten für den Patienten von Nachteil wäre oder der Patient auf andere Art und Weise bezüglich der Krankheitserscheinungen und ihrer Behandlung von einem Studienabbruch profitieren würde.
- Eintritt einer Schwangerschaft
- jede andere Situation, bei der nach Ansicht des Prüfarztes eine weitere Teilnahme an der klinischen Prüfung nicht im besten Interesse des Patienten sein würde
- Umstände für eine therapeutische Intervention, die durch den Prüfplan nicht zugelassen sind
- Nachträgliches Auftreten eines Ausschlusskriteriums
- signifikante Protokollverletzungen (z.B. Verabreichung der Studienmedikamente nicht möglich, Nichtdurchführen der Operation, vorzeitige Entblindung, etc.)

5.5.2 Vorzeitiger Abbruch der gesamten klinischen Prüfung

Die gesamte Studie kann abgebrochen werden, wenn der Prüfarzt dies aus einem der folgenden Gründe für erforderlich hält:

- Medizinische oder ethische Gründe, die die Fortsetzung der Studie gefährden
- Probleme bei der Rekrutierung von Patienten
- Auftreten von unerwünschten Ereignissen, die bisher in ihrer Art, dem Schweregrad, der Dauer oder der Häufigkeit nicht in Zusammenhang mit dem Sicherheitsprofil von MUTAGRIP® 2008/2009 oder LEUKINE® bekannt waren.

Nach bisheriger Datenlage sowie Erfahrungen aus Vorläuferstudien an unserer Klinik ist ein Abbruch der gesamten klinischen Prüfung nicht zu erwarten.

5.6 Lagerung, Aus- und Rückgabe

Die Ausgabe und Verblindung der Studienmedikation wird von der Apotheke durchgeführt. Für jeden einzelnen Patienten wird ein Protokoll über den Verbleib der Studienmedikation ab Annahme von der Apotheke bis zur Verabreichung geführt. Das entsprechende Protokoll wird im Prüfarztordner abgelegt.
Nach der Ausgabe durch die Apotheke wird das entsprechende Prüfpräparat durch den zuständigen Prüfarzt dem Patienten ohne Zwischenlagerung verabreicht.
Ebenfalls wird für jeden einzelnen Patienten eine Liste zum Verbleib der Studienmedikation geführt ab der Rückgabe vom Patienten bis zur Kontrolle durch den Monitor. Danach werden die dem Studienpatienten verabreichten Perfusorspritzen und Spritzen vom Prüfarzt dokumentiert vernichtet. Die Vernichtung der Prüfpräparate wird von der Apotheke durchgeführt und protokolliert

5.7 Compliance

Die Studienmedikation wird wie oben beschrieben von dem verantwortlichen Prüfarzt verabreicht, demzufolge ist die Compliance gewährleistet.

5.8 Placebo / Vergleichsmedikation

Die Verblindung, Ausgabe und Etikettierung erfolgt wie bei den Prüfpräparaten durch die Klinikapotheke der Charité –Universitätsmedizin Berlin, Campus Virchow-Klinikum.
Die Zubereitung der Vergleichsmedikation vor der Anwendung erfolgt ebenfalls durch die hiesige Klinikapotheke.

5.9 Begleitmedikation / Begleittherapie

Zulässig sind alle Medikamente die nicht eines der Ausschlusskriterien erfüllen. Alle Begleitmedikamente werden im ( )CRF protokolliert. Eine Medikation, die nicht der Standardtherapie nach dem jeweiligen Eingriff entspricht bzw. nicht im Zusammenhang mit Vorerkrankungen steht, wird in Verbindung mit einer medizinischen Diagnose als unerwünschtes Ereignis gewertet und dokumentiert.

5.10 Verblindung und Notfallumschläge

Verblindung

Die Ausgabe, Etikettierung sowie Verblindung der Studienmedikation wird von der Klinikapotheke der Charité – Universitätsmedizin Berlin, Campus Virchow-Klinikum durchgeführt.

Notfallumschläge

Der Prüfarzt erhält ein verschlossenes Kuvert mit der Notfall-Dekodierung jedes Patienten, um bei notwendigen Abweichungen von der vorgesehen Therapie (Protokollverletzungen) oder eventuell aufgetretenen schwerwiegenden unerwünschten Ereignissen (SAE) im Notfall feststellen zu können, ob ein Zusammenhang mit der Einnahme der Prüfmedikation vorliegen könnte bzw. auszuschließen ist. Wird ein solches Kuvert aus irgendeinem Grunde geöffnet, so ist dies im Studienprotokoll zu notieren. Sollte die Entblindung eines Studienteilnehmers notwendig werden, scheidet dieser als „dop-out“ aus der Studie aus.

5.11 Entblindung (Brechung des Codes)

Vorzeitige Entblindung
Die Randomisierungsliste wird in der Apotheke und beim Biometriker der Studie verschlossen aufbewahrt. Die Entblindung kann jedoch jederzeit erfolgen, schnell erreichbare Notfallkuverts werden in einem verschlossenen Schrank im Forschungssekretariat der Universitätsklinik für Anästhesiologie mit Schwerpunkt operative Intensivmedizin, Charité Campus Virchow-Klinikum, hinterlegt.

Reguläre Entblindung
Die reguläre Entblindung erfolgt nach dem Schließen der Datenbank.

6 Studienablauf

Ergänzend zu den hier gemachten Ausführungen wird auf das Ablaufschema der *Synopsis*, das *Studiendesign* (Kapitel 3.3) sowie das *Behandlungsschema* (Kapitel 5.4.1) verwiesen.

6.1 Verfahren des „Run-In“ (Prä-Screening)

Der Operationsplan der Klinik für Allgemein-, Visceral- und Transplantationschirurgie, Campus Virchow-Klinikum, Charité – Universitätsmedizin Berlin wird auf einschlussfähige Patienten gescreent. Die Patienten werden nach Aufnahme in der Charité – Universitätsmedizin Berlin, Campus Virchow-Klinikum, von einem Studienarzt der Universitätsklinik für Anästhesiologie mit Schwerpunkt operative Intensivmedizin, Campus Virchow Klinikum und Campus Charité Mitte, Charité –Universitätsmedizin Berlin, im Anschluss an das Narkoseaufklärungsgespräch auf die geplante Studie aufmerksam gemacht und über den Inhalt der Studie, sowie mögliche Risiken mündlich und schriftlich aufgeklärt. Desweiteren werden aktuelle Begleitmedikation sowie bekannte Erkrankungen des Patienten (Anamnese) insbesondere im Hinblick auf die Ein- und Ausschlusskriterien hin abgefragt. Nach erfolgter Einwilligung in die Studienteilnahme durch den Patienten erfolgt die erste Blutentnahme (BE0). Im Rahmen dieser Blutentnahme werden folgende Laborparameter erhoben, die zur Überprüfung spezieller Ein- und Ausschlusskriterien notwendig sind:

- Screening auf Hepatitis B (anti-HBs und HBs-Antigen)
- Screening auf Hepatitis C (anti-HCV)
- Screening auf HIV (anti-HIV1 und anti-HIV2)
- Thrombozyten
- Neutrophile
- Hämoglobin
- Bilirubin
- Kreatinin
- ASAT/ALAT

Bei weiblichen Studienteilnehmern wird zum Ausschluss einer Schwangerschaft der Urin auf βHCG intersucht.

6.1.1 Verfahren der Einwilligung nach Aufklärung

Die mündliche und schriftliche Aufklärung des Patienten durch den Studienarzt der Universitätsklinik für Anästhesiologie mit Schwerpunkt operative Intensivmedizin, Campus Virchow-Klinikum und Campus Charité Mitte, Charité – Universitätsmedizin Berlin, erfolgt im angemessenen Zeitabstand zur Intervention, mindestens aber 12 Stunden zuvor, sodass eine ausreichende Bedenkzeit bis zur geplanten Studienintervention gewährleistet ist.

6.1.2 Verfahren zur Vermeidung von zeitgleichem Einschluss in mehrere Studien

Die zeitgleiche Teilnahme an einer anderen AMG-Studie ist als Ausschlusskriterium definiert. Wenn der Patient an anderen Studien teilgenommen hat, ist es erforderlich, dass die Teilnahme an der vorhergehenden Studie einschließlich aller Nachuntersuchungen mindestens eine Woche vor Einschluss in diese Studie beendet worden ist.

6.2 Verfahren des Screenings

Das Screening beginnt mit dem Tag der Operation. Spätestens am Morgen vor der Operation liegen die Laborergebnisse des „Run-In“, die über eine weitere Studienteilnahme des Patienten entscheiden vor. Am Morgen der Operation (6.30 Uhr) erfolgt eine zweite Blutentnahme (BE1) zur erstmaligen Bestimmung der definierten Zielparameter (siehe auch *Laboruntersuchungen* im Kapitel 6.7.4).

Bzgl. der Behandlung und Versorgung des Patienten während der Operation sowie während seines intensivstationären Aufenthaltes durch die Universitätsklinik für Anästhesiologie mit Schwerpunkt operative Intensivmedizin, Campus Virchow-Klinikum, Charité – Universitätsmedizin Berlin sei auf die Standards unserer Klinik (SOPs) verwiesen, die im Kapitel 6.5 abgebildet sind.

Am Morgen des ersten postoperativen Tages (ca. 6.30 Uhr) erfolgt die dritte Blutentnahme (BE2) zur erneuten Bestimmung der definierten Zielparameter. Sollte der Patient im Rahmen dieser Blutentnahme eine HLA-DR-Expression von ≤ 10.000 Antigene/Monozyt aufweisen, ist damit auch das letzte Einschlusskriterium erfüllt und es erfolgt der Randomisierung des Patienten in die Studie. Damit endet die Screeningphase.

()

6.3 Randomisierung (Zuteilung der Studienmedikation) (1. Visite)

Die Randomisierung in die vorgesehenen Behandlungsarme (*Studiendesign* Kapitel 3.3) erfolgt in Form von Blockbildung. Die Randomisierungsliste wird durch den Studienkoordinator (Biometriker) verwaltet. Der Studienkoordinator übergibt der Apotheke eine Liste mit den Randomisierungsnummern und der jeweiligen Zuteilung zu einer der drei Behandlungen. Weder dem behandelnden Arzt noch dem Patienten ist diese Liste zugänglich. Der Verpackung der Studienmedikation ist ebenfalls keinerlei Informationen über die Art der Therapie zu entnehmen. Bei Aufnahme des Patienten in die Studie wird ihm die laufende Patienten-Nummer (= Randomisierungsnummer) zugeordnet. Der Patient behält diese Nummer während

der gesamten Prüfung. Ebenso wird zu den Randomisierungsnummern weder eine hinzugefügt noch weggenommen. Wenn die klinische Prüfung bei einem Patienten aus medizinischen oder anderen Gründen abgebrochen werden muss, wird dafür kein neuer Patient in die Prüfung aufgenommen.
Das Protokoll sieht eine Stratifizierung in Bezug auf Pankreasresektion und Ösophagusresektion vor.

6.4 Patienteneinschluss (Enrolment)

Patienten, die alle Einschlusskriterien erfüllen und ihr Einverständnis zur Teilnahme an der Studie gegeben haben, werden in die Studie aufgenommen und in der Regel dem Hauptprüfer der klinischen Prüfung gemeldet.
Die Meldung erfolgt in Form der Übermittlung des Registrierungsbogens (siehe Appendices). Dieser Registrierungsbogen enthält folgende Angaben:

- Bezeichnung und Anschrift des zuständigen Hauptprüfers
- Prüfzentrum, Prüfarzt
- Randomisierungsnummer
- Geschlecht
- Alter
- Körpergewicht
- Körpergröße
- BMI
- Aufnahme Krankenhaus
- OP-Tag
- Diagnose

6.5 Behandlung der Studienpatienten

Vor und während der Operation sowie im postoperativen Verlauf werden alle Studienpatienten nach den Standards der Universitätsklinik für Anästhesiologie mit Schwerpunkt operative Intensivmedizin, Charité – Universitätsmedizin Berlin, behandelt.

6.5.1 Pankreasresektion

Prämedikation
Anamnese bezüglich Magenausgangstenose erheben,<br>Chronische Pankreatitiden sind häufig chronischen Schmerzen und mit Alkoholkrankheit assoziiert<br>Midazolam p. o; ggf. Aspirationsprophylaxe mit Ranitidin und Metoclopramid<br>Aufklärung: PDK, ZVK,

Narkoseeinleitung

Anschluss des Monitorings und periphervenöser Zugang am Arm

Infusionsbeginn

PDK-Anlage im Sitzen, Testdosis 3ml Bupivacain 0,5%

Fentanyl1-2 µg/kg KG

Thiopental 3-5 mg/kg KG Bolus, evtl. Etomidate 0,15-0,3 mg/kg KG

Rocuronium 0,6 mg/kg KG, ggf. RSI mit Succinylcholin

Intubation

Magensonde nasal legen

ZVK-Anlage V. jugularis interna

Gabe des Antibiotikums

2. periphervenöser Zugang

Anlage Blasenkatheter

Augenschutz

Narkoseführung

Balancierte Anästhesie mit Desfluran in O_2-Luft-Gemisch

Beatmung: O_2/ Luft-Gemisch, FiO_2:0,5-0,8, $P_{et}CO_2$ 35-45mmHg
PEEP 5 cm H_2O

kombiniert mit

PDA: [Ropivacain 0,2% mit Sufentanil 1µg/ml] 6-8ml Bolus, dann kontinuierliche Laufrate 6-8ml/h

Relaxierung nach Bedarf mit Rocuronium,

Wiederholung der AB-Prophylaxe nach 3 Stunden

Postoperatives Management

Extubation anstreben, postoperativ Verlegung auf Intermediate Care Station, ITS oder PACU

Diagnostik/ Labor

Standardlabor

Spezielles Labor: direkt post-op: Lipase, Protein

1. post-op Tag: Lipase, Protein, Albumin, AST, ALT, Bili, AP, γGT

2. post-op Tag: Lipase, Bili (nach Sekretbefund) aus Easy Flow (und zugleich aus Serum): Amylase,

Rö Thorax nach ZVK-Anlage

**Monitoring**
Standardmonitoring + invasive Blutdruckmessung + ZVD

Standardtherapie

- Sandostatin 3x 100 µg **s.c.** zur Hemmung der Pankreas-Sekretion nach RS mit dem Operateur (**Cave**: COPRA bietet nur i.v.-Gabe an, manuell ändern!)
- BZ-Kontrollen, strenge Insulintherapie (besonders bei Pankreatektomie)
- OP-Tag:

Anlage Engmaschig Ernährungssonde/Frekasonde intraoperativ

Lagekontrolle im Röntgenbild des Thorax

initial 25 ml/h Fresubin über Sonde, wenn Ernährungssonde distal der Anastomose

- Ab 1. Post-op Tag:

Fresubin-auf 1 kcal/kg/h steigern

- 5. Tag post-op nach **Pankreaticogastrostomie** Anastomosendarstellung mit Gastrografin am Magenplatz; bis dahin **Magensonde sichern und tief hängen**!!!
- Bei Pankreatikojejunostomie: besondere Gefahr der Anastomoseninsuffizienz
- Abführen: tgl. Klysma, frühestens ab 3. post-op Tag durch Einlauf unterstützen; medikamentöse Stimulation nur in RS mit Operateur

6.5.2 Ösophagusresektion

**Narkoseeinleitung**
Patientenidentifizierung<br>Periphervenöser Zugang und Infusion<br>Anschluss des Monitorings<br>PDK Anlage: Th 5/6/Th 6/7 bei thorakaler Resektion<br>Th 6/7/Th 8/9 bei abdomineller Resektion<br>**Einleitung:**<br>Fentanyl 0,15-0,2 mg<br>Propofol 2mg/kg KG<br>Rocuronium 0,6 mg/kg KG<br>Intubation (bei DLT die Tubuslage fiberoptisch prüfen)<br>TIVA starten mit Propofol 5-10mg/kg KG /h + Fentanyl je 0,1-0,15 mg nach Bedarf<br>Arteriellen Zugang nach Narkoseeinleitung: (A. radialis sinistra), ggf. Anlage in Lokalanästhesie<br>ZVK-Anlage nach Narkoseeinleitung: (V. jugularis interna dextra)<br>PDK vor Lagerung des Patienten im Saal anspritzen:<br>Bolusapplikation von 4-8 ml der Stammlösung (Ropivacain 0,2% +Sufentanil 1µg/m) via PDK, anschließend kontinuierliche Gabe mit 6-8 (10) ml/h<br>Magensonde transnasal platzieren, endgültige Positionierung erfolgt intraoperativ nach Kontrolle durch Operateur<br>Blasenkatheteranlage<br>Augenschutz (Augensalbe, vollständiger Lidschluß)

Narkoseführung

Beatmung: Sauerstoff-Luft-Gemisch (FiO_2: 0,5-0,8´), $etCO_2$: 35-45 mmHg

PEEP 5-8 mm H_2O

Bei 2-Lungenventilation AZV 6-8ml/kg KG

Bei 1-Lungenventilation FiO_2 zunächst 1,0, dann nach BGA ggf. reduzieren

AZV 6-8 ml/kg KG, auf Normocapnie achten, ggf. drucklimitierte Beatmung PIP≤ 30 cmH_2O

1. TIVA-PDA: Propofol initial 10mg/kg KG/h, dann stufenweise Reduktion bis min. 5mg/kg KG/h. bei Manipulationen an der Nase, z.B. Platzierung der Magensonde, zusätzliche Gabe von Fentanyl
2. PDA: Ropivacain (0,2% +Sufentanil 1µg/ml) über PDK mit 6-8 (10) ml/h
3. Muskelrelaxanziengabe: Rocuronium nach Relaxometrie
4. Magensondenposition und Dichtigkeitstest mit Methylenblau nach Absprache mit dem Operateur.

Postoperatives Management

Die unmittelbar postoperative Extubation wird angestrebt
Postoperative Überwachung auf der Intensivstation
Effektive Schmerztherapie (PDK oder PCA)

Lage der Thoraxdrainagen festhalten und der ITS übergeben,

Enterale Frühernährung über intraoperativ platzierte Duodenalsonde nach Absprache mit dem Operateur, (mindestens 5 Tagen absolute orale Nahrungskarenz)

Diagnostik/ Labor

Standardlabor + Spezielles Labor: KOD

Rö Thorax

Monitoring

Standardmonitoring + invasive Blutdruckmessung + ZVD

Standardtherapie / Management
• Restriktive Volumentherapie (Lungenödem/ Pleuraergüsse durch Unterbrechung der ösophagealen Lymphbahnen) aber Normovolämie (ITBVI-Ziel 800-900)<br>• OP-Tag:<br>Enterale Frühernährung falls intraoperativ eine jejunale Ernährungssonde (z.B. Freka Sonde) platziert wurde nach Rücksprache mit dem Operateur<br>Lagekontrolle im Röntgenbild des Thorax<br>Initial 25 ml/h Fresubin über Sonde, wenn Ernährungssonde distal der Anastomose (Rücksprache mit dem Operateur)<br>• Am 5. post-op Tag nach Anastomosendarstellung mit Gastrografin am Magenplatz; bis dahin **Magensonde sichern + auf Ablauf** (Schienung der Anastomose); absolute orale Nahrungskarenz!!!<br>**Cave:** Schluckstörungen/ Mikroaspirationen vor allem bei kollaren Anastomosen<br>• Abführen:: täglich Klysma; wenn kein spontaner Stuhlgang, frühestens ab 3. post-op Tag durch Einlauf unterstützen; medikamentöse Stimulation nur in RS mit Operateur<br>• Häufig Schulterschmerzen durch intraoperative Lagerung; durch PDK nicht erfasst<br>• Regelmäßige Röntgen Thorax Kontrollen. Hohes Risiko für Belüftungsstörungen, Pneumonien<br>• Clonidinperfusor mit OP-Beginn, falls kein PDK<br>• **Cave:** bei Ösophagus-Ca i.d.R. Delirprophylaxe erforderlich:<br>o Vegetative Symptomatik: Clonidin (30- 90 µg/h)<br>o Haluzinatorische Symptomatik: Haloperidol i.v.<br>o Angstsymptomatik: Benzodiazepine (z.B. Tavor i.v.)

6.5.3 Definitionen behandlungsrelevanter Infektionen

Pneumonie

Die Diagnose erfolgte anhand der HAP-Kriterien (2005). Dabei muss eines der folgenden Kriterien erfüllt sein:
Für die Diagnose folgender Infektionen gelten die CDC-Definitionen (5. Auflage, Berlin, 2005, Herausgeber: Robert-Koch-Institut)

Bronchitis, Tracheobronchitis, Tracheitis

Patient zeigt keine für die Diagnose einer Pneumonie ausreichenden klinischen oder röntgenologischen Anzeichen und hat **zwei** der folgenden Symptome ohne andere erkennbare Ursache:

- Fieber (> 38° C)
- Husten
- neue oder erhöhte Sputumproduktion
- trockene Rasselgeräusche
- Giemen

und eines der folgenden Kriterien:

- Kultureller Nachweis von Erregern aus Trachealsekret oder bronchoalveolärer Lavage.
- Positiver Antigennachweis in relevanten Atemwegssekreten.

Wundinfektionen

Postoperative oberflächliche Wundinfektion

Infektionen an der Inzisionsstelle innerhalb von 30 Tagen nach der Operation, die nur Haut oder subkutanes Gewebe mit einbezieht,
und eines der folgenden Kriterien trifft zu:

1. Eitrige Sekretion aus der oberflächlichen Inzision
2. Kultureller Nachweis eines Mikroorganismus aus einem aseptisch entnommenen Wundsekret oder Gewebekultur von der oberflächlichen Inzision
3. Eines des folgenden Anzeichen: Schmerz oder Berührungsempfindlichkeit, lokalisierte Schwellung, Rötung oder Überwärmung und Chirurg öffnet die oberflächliche Inzision bewusst. Dieses Kriterium gilt jedoch nicht bei Vorliegen einer negativen mikrobiologischen Kultur von der oberflächlichen Inzision.
4. Diagnose des Chirurgen

Postoperative tiefe Wundinfektion

Infektion innerhalb von 30 Tagen nach der Operation (innerhalb von 1 Jahr wenn Implantat in situ belassen),
und
Infektion scheint mit der Operation in Verbindung zu stehen
und
erfasst Faszienschichten und Muskelgewebe. Zudem muss eines der folgenden Kriterien erfüllt sein:

1. Eitrige Sekretion aus dem tiefen Einschnitt, aber nicht aus dem operierten Organ bzw. der Körperhöhle.
2. Spontan oder vom Chirurgen bewusst geöffnet, wenn der Patient mindestens eines der nachfolgenden Symptome hat: Fieber (> 38° C), lokalisierter Schmerz oder Berührungsempfindlichkeit. Dieses Kriterium gilt jedoch nicht bei Vorliegen einer negativen mikrobiologischen Kultur aus der Tiefe der Inzision.
3. Abszess oder sonstige Zeichen der Infektion, die tieferen Schichten betreffend, sind bei der klinischen Untersuchung, während der erneuten Operation, bei der phytopathologischen Untersuchung oder bei radiologischen Untersuchungen ersichtlich.
4. Diagnose des behandelnden Arztes.

Symptomatische Harnwegsinfektion

muss einem der folgenden Kriterien entsprechen:

1. Eines der folgenden Anzeichen ohne andere erkennbare Ursache: Harndrang, erhöhte Miktionsfrequenz, Dysurie oder suprapubische Missempfindungen
   und
   eine Urinkultur $>10^5$ Kolonien/ml Urin mit nicht mehr als zwei Spezies von Mikroorganismen.
2. Zwei der folgenden Anzeichen ohne andere erkennbare Ursache: Harndrang, erhöhte Miktionsfrequenz, Dysurie oder suprapubische Missempfindungen
   und
   mindestens eines der folgenden Kriterien:

- Harnteststreifen für Leukozytenesterase und/oder Nitrat positiv
- Pyurie (>10 weiße Blutkörperchen/ml^3 oder >3 Leukozyten/Gesichtsfeld bei starker Vergrößerung im nicht zentrifugiertem Urin)
- Bei Gram-Färbung einer nicht zentrifugierten Urinprobe Nachweis von Mikroorganismen.
- zwei Urinkulturen mit wiederholter Isolierung des gleichen Uropathogens mit $>10^2$ Kolonien/ml Urin im Katheterurin
- Urinkultur mit $>10^5$ Kolonien/ml Urin einzelner Uropathogene bei Patienten, die mit der entsprechenden antimikrobiellen Therapie behandelt werden

- Diagnose des Arztes
- Arzt beginnt entsprechende antimikrobielle Therapie

Sinusitis
muss einem der folgenden Kriterien entsprechen:
1. Kultureller Nachweis von Erregern im eitrigen Sekret der Nasennebenhöhle.
2. Eines der folgenden Anzeichen ohne andere erkennbare Ursache: Fieber (> 38° C), Schmerz oder Empfindlichkeit im Bereich der betroffenen Nebenhöhle, Kopfschmerzen, eitriges Exsudat oder Obstruktion der Nase
   und mindestens eines der folgenden:
   - Diaphonoskopie positiv.
   - Radiologischer Hinweis auf Infektion.

Sepsis
Sepsis wird entsprechend den Kriterien der „Society of Critical Care Medicine Consensus Conference" definiert:
- Atemfrequenz: <20/min oder P_aCO_2 <32mmHg oder mechanische Ventilation
- Herzfrequenz: >90/min ohne ß-Blockade
- Körpertemperatur: >38°C oder <36°C
- Leukozytenzahl: >12000/mm^3 oder <4000/mm^3 oder >10% unreife Neutrophile
- Systemische Toxizität oder schlechte Organperfusion mit zwei oder mehr charakteristischen Merkmalen:
  - Akutes Nierenversagen (Oligurie <0,5 ml*kg^{-1}*h^{-1})
  - Erhöhtes Plasmalaktat (>1,8 mmol/l)
  - CI >4,0 l/min mit SVR <800 dyn*s*cm^{-5}
  - Metabolische Azidose (pH <7,3 oder Basendefizit >5)
  - Arterielle Hypoxie: P_aO_2 <75 mmHg (<10kPa)
  - Thrombozytenabfall innerhalb der letzten 24h ohne anderweitige Erklärung (<100000/ml oder Abfall von 50% vom Ausgangswert)
  - Abnorme Gerinnungswerte innerhalb der letzten 24h ohne anderweitige Erklärung (Quick >1,5 oder PTT >1,2*Kontrollwert)
  - Plötzlicher Abfall des Glascow-Coma-Scale
  - Hypotension, die anhand eines der folgenden Kriterien definiert wurden:
    - Systolischer Blutdruck <90 mmHg
    - Anhaltender Blutdruckabfall von 40 mmHg mit entsprechendem Flüssigkeitsbedarf ohne Antihypertensiva
    - Therapie mit Vasopressoren, um den Blutdruck über 90 mmHg zu halten
  - Die Sepsis als Diagnose ist mit dem klinischen Bild vereinbar.

6.6 Klinische Untersuchungen und Abweichungen von der üblichen klinischen Praxis

Während des gesamten Zeitraumes der Studienteilnahme (prä-, peri- und postoperativ) erfolgt die Behandlung der Patienten gemäß der Standards der Universitätsklinik für Anästhesiologie mit Schwerpunkt operative Intensivmedizin, Campus Virchow-Klinikum und Campus Charité Mitte, Charité – Universitätsmedizin Berlin.
Die laut Studienprotokoll geplanten Interventionen (Blutabnahmen, Verabreichung der Studienmedikation) sind rein additiver Natur und beeinträchtigen in keinster Weise die dem Patienten zukommende Standardbehandlung während seines Klinikaufenthaltes. Bis auf die geplanten additiven Interventionen sind Abweichungen von der üblichen klinischen Praxis während der gesamten Studienteilnahme ausdrücklich nicht vorgesehen.

Bzgl. des studienrelevanten Untersuchungs- und Beobachtungsprogramms sowie der vorgesehenen Zeitpunkte bzw. Intervalle der Visiten sei auf das *Ablaufschema* (Kapitel 1) verwiesen.

Kontinuität der Untersuchungsmethode
Die Anwendung des gleichen Verfahrens für alle Untersuchungszeitpunkte ist zu fordern.

Evaluationskriterien / Remissionskriterien
Das Vorgehen zur Klassifizierung des Krankheitsverlaufs ist genau festzulegen.

Zeitplan
Bezüglich der Zeitpunkte oder -intervalle vor, während und nach der Behandlung bzw. der Therapiezyklus-abhängigen Zeitpunkte sei auf das *Ablaufschema* (Kapitel 1) sowie das *Behandlungsschema* (Kapitel 5.4.1) in diesem Antrag verwiesen.

6.6.1 1. Visite

Die erste Visite findet am ersten postoperativen Tag statt. Nach erfolgter *Randomisierung* (siehe Kapitel 6.3) erfolgt die erste Applikation des entsprechenden Prüfpräparates (SM1). Des Weiteren erfolgt die Protokollierung aller intensivstationären Parameter, die entsprechend den Standards der Universitätsklinik für Anästhesiologie mit Schwerpunkt operative Intensivmedizin, Campus Virchow-Klinikum und Campus Charité Mitte, Charité – Universitätsmedizin Berlin, erhoben werden, in den entsprechenden *( )CRF* (Kapitel 10.1). Es erfolgt ebenfalls die Dokumentation eventuell auftretender AEs, SAEs und SUSARs.

6.6.2 2. und 3. Visite

Die zweite und dritte Visite findet am zweiten bzw. dritten postoperativen Tag statt. An beiden Tagen wird jeweils eine Blutentnahme zur Bestimmung der definierten Zielparameter durchgeführt (BE3 und BE4). Direkt im Anschluss an die jeweilige Blutentnahme erfolgt die Applikation des Prüfpräparates (SM2 und SM3). Desweiteren erfolgt die Protokollierung aller intensivstationären Parameter, die entsprechend den Standards der Universitätsklinik für Anästhesiologie mit Schwerpunkt operative Intensivmedizin, Campus Virchow-Klinikum und Campus Charité Mitte, Charité – Universitätsmedizin Berlin, erhoben werden, in den entsprechenden *eCRF* (Kapitel 10.1). Es erfolgt ebenfalls die Dokumentation eventuell auftretender AEs, SAEs und SUSARs.

6.6.3 4. und 5. Visite

Die vierte und fünfte Visite findet am vierten bzw. fünften postoperativen Tag statt. An beiden Tagen wird jeweils eine Blutentnahme zur Bestimmung der definierten Zielparameter durchgeführt (BE5 und BE6). Desweiteren erfolgt die Protokollierung aller intensivstationären Parameter, die entsprechend den Standards der Universitätsklinik für Anästhesiologie mit Schwerpunkt operative Intensivmedizin, Campus Virchow-Klinikum und Campus Charité Mitte, Charité – Universitätsmedizin Berlin, erhoben werden, in den entsprechenden *eCRF* (Kapitel 10.1). Es erfolgt ebenfalls die Dokumentation eventuell auftretender AEs, SAEs und SUSARs.

6.6.4 6. bis 8. Visite

Die sechste bis achte Visite findet am sechsten bis achten postoperativen Tag statt. Hierbei erfolgt die Protokollierung aller intensivstationären Parameter, die entsprechend den Standards der Universitätsklinik für Anästhesiologie mit Schwerpunkt operative Intensivmedizin, Campus Virchow-Klinikum und Campus Charité Mitte, Charité – Universitätsmedizin Berlin, erhoben

werden, in den entsprechenden *eCRF* (Kapitel 10.1). Es erfolgt ebenfalls die Dokumentation eventuell auftretender AEs, SAEs und SUSARs.

6.6.5 9. Visite (Abschlussuntersuchung)

Die Abschlussuntersuchung am 9. postoperativen Tag umfasst eine zusammenfassende Beurteilung des Gesundheitszustandes des Patienten vor Entlassung aus der Studie insbesondere im Hinblick auf stattgehabte oder immer noch präsente AEs, SAEs oder SUSARs, unabhängig davon, ob ein Zusammenhang mit einer der studienbedingten Maßnahmen angenommen wird. Diese Untersuchung wird bei allen Studienpatienten, soweit möglich, durchgeführt, auch bei Studienpatienten, die vorzeitig aus der Studie ausscheiden (bei freiwillig ausscheidenden Patienten mit deren Zustimmung).
Das Studienende ist für jeden Patienten mit erfolgter Abschlussuntersuchung geplant.

6.6.6 Follow-Up

Die Studie endet für den einzelnen Patienten am neunten postoperativen Tag oder bei vorheriger Entlassung / Verlegung in eine Klinik außerhalb der Charité – Universitätsmedizin Berlin sowie bei vorzeitigem Studienabbruch (siehe auch Kapitel 8.1 und 8.2). Ein generelles „Follow-Up“ wird nicht durchgeführt. Mit der Abschlussuntersuchung am 9. postoperativen Tag noch nicht abgeschlossene AEs werden im Rahmen eines „Follow-Up“ von 7 Tagen, noch nicht abgeschlossene SAEs im Rahmen einer „Follow-Up von 21 Tagen, nachverfolgt (siehe auch Kapitel 1 und 9.4). Dem Vertreter des Sponsors bleibt es vorbehalten im Einzelfall von den hier festgelegten Follow-Up-Fristen abzuweichen.

6.6.7 Laboruntersuchungen

Entsprechend der im Behandlungsschema definierten Zeitpunkte zur Blutentnahme (BE1 – BE6) wird die Bestimmung jeweils folgende Parameter von den zuständigen Labors der Charité – Universitätsmedizin Berlin durchgeführt:

Laboruntersuchungen für die ersten 33 eingeschlossenen Patienten der Behandlungsgruppen:

Nr.	Name	Einheit / Maß	Referenzbereich
**Blutbild (im Zentrallabor bestimmt)**			
01	Leukozyten	/nl	
**Diff. Blutbild, manuell (im Zentrallabor bestimmt)**			
02	Myelozyten	%	
03	Stabkernige	%	
04	Segmentkernige	%	
05	Lymphozyten	%	
06	Monozyten	%	
**Durchflusszytometrie**			
**Diff. Blutbild**			
07	Lymphozyten rel.	%	
08	Lymphozyten abs.	/nl	1,5 – 3,0
09	Monozyten rel.	%	2- 10
10	Monozyten abs.	/nl	0,0 – 0,5
11	Granulozyten rel.	%	50 – 80
12	Granulozyten abs.	%	3,0 – 6,5
**Lymphozytensubpopulationen**			
13	CD3+ T-Zellen abs.	/nl	0,9 – 2,2
14	CD3+ T-Zellen in % der Lymphozyten	%	60 – 85
15	CD4+ T-Zellen in % der T-Zellen	%	

16	CD4+ T-Zellen abs	/nl	0,5 – 1,2
17	CD4+ T-Zellen in % der Lymphozyten	%	30 – 60
18	CD8+ T-Zellen in % der T-Zellen	%	
19	CD8+ T-Zellen abs.	/nl	0,3 – 0,8
20	CD8+ T-Zellen in % der Lymphozyten	%	20 - 40
21	CD8+ CD4+ T-Zellen in % der T-Zellen	%	< 10
22	CD8- CD4- T-Zellen in % der T-Zellen	%	< 15
23	CD4/CD8 Ratio		1,1 – 3,0
24	CD 19+ B-Zellen abs.	/nl	0,1 – 0,4
25	CD19+ B-Zellen in % der Lymphozyten	%	5 – 25
26	CD 16+ NK-Zellen abs.	/nl	0,1 – 0,4
27	CD16+ NK-Zellen in % der Lymphozyten	%	5 – 25
**Monozytäre Immunkompetenz**			
28	HLA-DR – Expression / Monozyten	Antigene/Zelle	> 15000
**T-Zellen (CD25/CD62/HLA-DR)**			
29	CD4+ T-Zellen abs.	/nl	
30	CD4+ CD25+ / CD4+ T-Zellen rel.	%	
31	CD4+ CD25+ abs.	/nl	
32	CD4+ CD62+ / CD4+ T-Zell rel.	%	
33	CD4+ CD62 abs.	/nl	
34	CD4+ CD25+ CD62+ / CD4+ T-Zellen rel.	%	
35	CD4+ CD25+ CD62+ abs.	/nl	
36	CD4+ CD25+ + CD62+(Treg)/CD4-Zellen rel.	%	
37	CD4+ CD25+ + CD62+ (treg) abs.	/nl	
**LÖSLICHE MEDIATOREN**			
**Heparin Plasma**			
38	Interleukin-10 (Immunlite)	pg/ml	
39	Tumornekrosefaktor-alpha (Immulite)	pg/ml	
**EDTA-Plasma**			
40	Interleukin-6 (Immulite)	pg/ml	
Nr.	Name	Einheit / Maß	Referenzbereich
**FUNKTIONSTESTE – Heparin-Blut**			
**Ex vivo-Vollblutstimulationstest (ConA 24h), T-Zellfunktion**			
41	Tumornekrosefaktor-alpha (CBA)	pg/ml	2400 – 3500
42	Interferon-gamma (CBA)	pg/ml	M: 3000 – 10000<br>F: 7500 – 10000
43	Interleukin-2 (CBA)	pg/ml	150 – 230
44	Interleukin-4 (CBA)	pg/ml	10 – 23
45	Interleukin-5 (CBA)	pg/ml	11 – 31
46	Interleukin-10 (CBA)	pg/ml	350 - 470
47	Interleukin-17 (CBA)	pg/ml	?
**Ex vivo-Vollblutstimulationstest (LPS 4h), Monozytenfunktion**			
48	Tumornekrosefaktor-alpha (Immulite)	pg/ml	-
**Ex vivo-Vollblutstimulationstest (LPS 24h), Monozytenfunktion**			
49	Interleukin-10 (immulite)	pg/ml	-
**Real-Time-PCR: Transkriptionsfaktoren und Regulatorproteine**<br>(Beachten Sie hierzu die Ausführungen zur *„Quantifizierung von Expressionsmustern“* im Kapitel 6.6.8)			
50	HPRT	$2^{-\Delta Ct}$	-
51	T-bet	$2^{-\Delta Ct}$	-
52	Foxp3	$2^{-\Delta Ct}$	-
53	Eomes	$2^{-\Delta Ct}$	-
54	GATA-3	$2^{-\Delta Ct}$	-
55	SOCS-1	$2^{-\Delta Ct}$	-
56	SOCS-3	$2^{-\Delta Ct}$	-
57	SOCS-5	$2^{-\Delta Ct}$	-
58	STAT-1	$2^{-\Delta Ct}$	-
59	STAT-3	$2^{-\Delta Ct}$	-
60	STAT-5	$2^{-\Delta Ct}$	-
61	IL-6	$2^{-\Delta Ct}$	-

62	IL-10	$2^{-\Delta Ct}$	-
63	IFNγ	$2^{-\Delta Ct}$	-
64	IL-23	$2^{-\Delta Ct}$	-
65	TNF-α	$2^{-\Delta Ct}$	-
66	NFκB	$2^{-\Delta Ct}$	-
67	RORγt	$2^{-\Delta Ct}$	-
68	TGF-ß	$2^{-\Delta Ct}$	-
69	PU.1	$2^{-\Delta Ct}$	-
70	IL-17	$2^{-\Delta Ct}$	-
**Infektiologie**			
71	anti-HIV1		-
72	anti-HIV2		-
73	anti-HBs	IU/ml	-
74	HBs-Antigen		-
75	anti-HCV		-
**Alkoholmarker** (Beachten Sie hierzu die Ausführungen im Kapitel 6.6.9)			
76	PEth*	µmol/l	< 0,36
77	EtG*	µmol/l	< 0,20
78	CDT*	%	< 2,5
79	GGT*	U/l	Männer < 55<br>Frauen < 38
80	MCV*	Fl	81-100
**Lösliche Mediatoren im Plasma**			
81	Procalcitonin	pg/ml	< 500
**Sonstige**			
82	Treg	-	-
83	Th17	-	-
**Wissenschaftliche Begleituntersuchungen** (Beachten Sie hierzu die Ausführungen im Kapitel 6.6.10)			
84*	TLR-2-Polymorphismus	-	-
85*	TLR-4-Polymorphismus	-	-
**Urin**			
86	ßHCG	-	-
87*	EtG	µmol/l	≤ 2,2

**entsprechende Parameter werden nur einmalig, im Rahmen der ersten Blutentnahme (BE1) bestimmt.*

Laboruntersuchungen für die folgenden eingeschlossenen Patienten der Behandlungsgruppen (ab 34. Studienpatienten) ():

Nr.	Name	Einheit / Maß	Referenzbereich
**Blutbild (im Zentrallabor bestimmt)**			
01	Leukozyten	/nl	
**Diff. Blutbild, manuell (im Zentrallabor bestimmt)**			
02	Myelozyten	%	
03	Stabkernige	%	
04	Segmentkernige	%	
05	Lymphozyten	%	
06	Monozyten	%	
**Durchflusszytometrie**			
**Diff. Blutbild**			
07	Lymphozyten rel.	%	
08	Lymphozyten abs.	/nl	1,5 – 3,0
09	Monozyten rel.	%	2- 10
10	Monozyten abs.	/nl	0,0 – 0,5
11	Granulozyten rel.	%	50 – 80
12	Granulozyten abs.	%	3,0 – 6,5
**Monozytäre Immunkompetenz**			
28	HLA-DR – Expression / Monozyten	Antigene/Zelle	> 15000

**Infektiologie**			
71	anti-HIV1		-
72	anti-HIV2		-
73	anti-HBs	IU/ml	-
74	HBs-Antigen		-
75	anti-HCV		-
**Alkoholmarker**<br>(Beachten Sie hierzu die Ausführungen im Kapitel 6.6.9)			
76	PEth*	µmol/l	< 0,36
77	EtG*	µmol/l	< 0,20
78	CDT*	%	< 2,5
79	GGT*	U/l	Männer < 55<br>Frauen < 38
80	MCV*	Fl	81-100
**Lösliche Mediatoren im Plasma**			
81	Procalcitonin	pg/ml	< 500
**Urin**			
86	ßHCG	-	-
87*	EtG	µmol/l	≤ 2,2

******entsprechende Parameter werden nur einmalig, im Rahmen der ersten Blutentnahme (BE1) bestimmt.*

6.6.8 Quantifizierung von Expressionsmustern

Bei den Untersuchungen der RNA- und Proteindaten (siehe Tabelle Punkt 50 – 60) handelt es sich um eine reine Quantifizierung von Expressionsdaten (Aktivität von Genen). Die Proben werden nicht auf Sequenzunterschiede hin untersucht. Damit handelt es sich hierbei nicht um eine „genetische Untersuchung“.

6.6.9 Alkoholmarker

Zur Objektivierung eines Alkoholkonsums stehen eine Reihe von Alkohol-Biomarkern zur Verfügung (siehe Tabelle Punkte 66 – 70 und 77). Die gängigsten Laborparameter zum Nachweis eines chronischen Alkoholkonsums sind die γ-GT, das MCV und das CDT im Blut. Keiner der Marker ist allein genügend sensitiv (MCV 34–89%, γ-GT 34–85%, CDT 39–94%) oder spezifisch (MCV 26–91%, γ-GT 11–85%, CDT 82–100%), wobei die Sensitivität und Spezifität immer jeweils vom untersuchten Patientenkollektiv abhängig ist [85]. Durch Kombination dieser Parameter lässt sich die Sensitivität und Spezifität zwar verbessern, einen chronischen Alkoholkonsum kann man jedoch mit diesen Marker allein trotzdem nicht nachweisen [86]. Unter bestimmten Umständen haben diese Marker außerdem eine zusätzlich verringerte Aussagekraft, z.B. sind bei einer fortgeschrittenen Leberfunktionseinschränkung sowohl eine erhöhte γ-GT als auch CDT nicht spezifisch für das Vorliegen einer Alkoholkrankheit [87]. Deutlich besser, weil hochspezifisch und sensitiv, ist daher die Bestimmung von Alkohol-Metaboliten im Blut und Urin. Durch eine Bestimmung der Konzentration von Phosphatidylethanol (PEth) im Blut kann ein starker Alkoholkonsum während eines mittelfristig zurückliegenden Zeitraums (2-3 Wochen) mit einer Spezifität von 100% und einer Sensitivität von 94,5% nachgewiesen werden [88]. Für den Nachweis eines kürzer zurückliegenden Alkoholkonsums eignet sich die Bestimmung der Konzentration von Ethyglucuronid (EtG) im Blut (ca. 12-24h) bzw. Urin (ca. 1 Woche) [89].
Die Messung von Alkoholmarkern sind Bestandteil des Charité-Algorithmus zum Screening auf gefährlichen oder schädlichen Alkoholkonsum bzw. Abhängigkeit der Universitätsklinik für Anästhesiologie mit Schwerpunkt operative Intensivmedizin, Campus Virchow-Klinikum und Campus Charité Mitte, Charité – Universitätsmedizin Berlin, für alle intensivstationären Patienten (siehe Appendix 12).

6.6.10 Wissenschaftliche Begleituntersuchung

Für viele Krankheiten existieren prädisponierende genetische Faktoren. Dies konnte durch Familien- und Zwillingsuntersuchungen festgestellt werden. In einer dänische Studie an Adoptivkindern wurde gezeigt, dass insbesondere Infektionskrankheiten eine starke genetische Komponente haben und Umwelteinflüsse weniger als angenommen zur Mortalität beitragen [90]. Natürlich vorkommende Sequenzvariationen (Polymorphismen) im Genom tragen dabei wesentlich zur Krankheitssuszeptibilität bei. Hierbei liegen 90% aller Polymorphismen als Variationen eines einzelnen Nukleotids („single nucleotide polymorphism“, SNP) vor [91]. SNPs werden definiert als Position im Genom, an denen sich die DNA bei normalen Individuen in einer einzelnen Base unterscheidet.
Die Erkennung von Pathogenen wird durch Rezeptoren des angeborenen Immunsystems vermittelt, die als „pathogen recognition receptors“ (PRRs) bezeichnet werden. Zu den PRRs gehören CD14, der „Macrophage Scavenger Receptor“ (MSR), Toll-like-Rezeptoren und Komplement-Rezeptoren [92].
Im Rahmen dieser Studie sollen solche Polymorphismen im TLR-2- und TLR-4-Gen bestimmt werden. Durch die Schlüsselstellung der Toll-like-Rezeptoren im angeborenen und adaptiven Immunsystem haben genetische Variationen in TLR-Genen einen Einfluss auf Immunabwehr und Pathogenese inflammatorischer Krankheiten. Insbesondere von den Rezeptoren TLR-2 und TLR-4 ist bekannt, dass sie in der Abwehr von Pathogenen von entscheidender Bedeutung sind. Während TLR-2 insbesondere Bestandteile der Wand gram-positiver Bakterien erkennt, werden gram-negative Keime von TLR-4 detektiert [34-38]. Mutationen von TLR-2 und -4 erhöhen das Risiko bakterieller Infektionen [39; 40]. Eine geringere Produktion proinflammatorischer Zytokine durch Monozyten scheint bei den Patienten mit Polymorphismen mitverantwortlich zu sein [41]. TLR-Polymorphismen sind von großer klinischer Relevanz, da ca. 10% der Bevölkerung Mutationsträger sind (Bornstein et al. 2004; Schröder et al., 2005).
Für TLR-2 und TLR-4 sind inzwischen eine Vielzahl von Polymorphismen entdeckt worden und in der SNP-Database des „National Center of Biotechnology Information“ (NCBI) hinterlegt. Die Blutentnahme für die Untersuchung auf TLR2- und TLR-4-Polymorphismen erfolgt am Tag der Operation, präoperativ (BE1). Für die DNA-Extraktion werden 2 ml Vollblut (EDTA) benötigt. Die genetische Analyse wird durchgeführt durch das Zentralinstitut für Laboratoriumsmedizin und Pathobiochemie, Charité – Universitätsmedizin Berlin, Campus Virchow-Klinikum (Leiter: Prof. Dr. med. Rudolf Tauber). Die Proben werden nach Analyse durch das Zentralinstitut für Laboratoriumsmedizin und Pathobiochemie der Charité durch dieses umgehend vernichtet. Der Patient kann jederzeit der Weiterverarbeitung seiner im Rahmen des wissenschaftlichen Begleitprogramms erhobenen Proben widersprechen und ihre Vernichtung verlangen.
Über diese wissenschaftliche Begleituntersuchung erhält jeder Patient im Rahmen der Aufklärung eine gesonderte Patienteninformation. Für diese Untersuchung wird dementsprechend eine gesonderte Einwilligung des Patienten benötigt (siehe Appendix Nr. 3). Die Teilnahme an dieser wissenschaftlichen Begleituntersuchung ist keine Voraussetzung für die generelle Studienteilnahme. Die ausschließliche Teilnahme an der wissenschaftlichen Begleituntersuchung ist nicht möglich.

6.7 Dauer der Studienteilnahme für den einzelnen Patienten

<u>Ende der Studienteilnahme</u>

Bezüglich des Zeitplanes der Studie, insbesondere die Dauer der Studienteilnahme für den einzelnen Patienten sei auf das *Ablaufschema* (Kapitel 1) sowie die Kapitel 6.6.5 und 6.6.6 in diesem Antrag verwiesen.

<u>Wechsel des Behandlungsarms</u>

Ein Wechsel des Behandlungsarms in einen anderen als den ursprünglich zugewiesenen ist nicht vorgesehen.

7 Risiko-Nutzen-Abwägung

Risiken, Nebenwirkungen, Belastungen, Vor- und Nachteile für den Teilnehmer

Multiple immunsuppressive Bedingungen wie neoplastische Grunderkrankungen und die Operation selbst, aber auch Nebendiagnosen wie chronischer Alkoholkonsum oder Komplikationen in der postoperativen Phase nach großen allgemeinchirurgischen Eingriffen, führen zu einem erhöhten Auftreten postoperativer Infektionen und steigern damit Morbidität und Mortalität dieses Patientenkollektives [5; 6; 9; 10]. In einer Vielzahl von Untersuchungen konnte gezeigt werden, dass große chirurgische Eingriffe regelhaft mit einer postoperativen Immundysbalance vergesellschaftet sind. Diese Immundysbalance kann letztendlich in eine Immundepression münden, wodurch der immunkompromittierte Zustand dieser Patienten zusätzlich verstärkt wird. Eine Immundepression ist nachweislich mit einem signifikant erhöhten Auftreten infektionsbedingter Komplikationen verbunden [8]. Insbesondere Patienten welche sich einer Pankreas- oder Ösophagusresektionen unterziehen, zeigen eine hohe Rate postoperativer Infektionen (50-60%) die in über 10% der Fälle zur Sepsis führen [11; 12]. Die Sicherheit inaktivierter Grippeimpfstoffe wurde in zahlreichen randomisierten Studien geprüft [13-15]. Unter Immunsuppression sind in der Regel Lebendimpfstoffe kontraindiziert. Der Grippeimpfstoff ist aber ein Totimpfstoff aus inaktivierten weil gespaltenen Grippeviren, weswegen eine virale Erkrankung durch die Impfbestandteile nicht zu befürchten ist. Bei Impfungen von Patienten verschiedener Altersstufen mit Lymphomen, Leukämien oder soliden Tumoren während oder nach Chemotherapie wurde über meist zufriedenstellende Impfresultate bei guter Verträglichkeit der Impfung berichtet. Im Rahmen von an unserer Klinik durchgeführten Vorläuferstudien konnte kein Zusammenhang zwischen dem Auftreten unerwünschter (AEs) oder schwerwiegender unerwünschter Ereignissen (SAEs) und einer mehrmaligen perioperativen Grippeimpfung beobachtet werden [1]. Verdachtsfälle unerwarteter schwerwiegender Nebenwirkungen traten im Verlauf der publizierten und der laufenden Studie nicht auf (Punkt 2.2.1 und 2.2.2)

Durch Vakzinierung mit Influenza-Impfstoff bzw. Verabreichung von GM-CSF zum Zeitpunkt der postoperativen Immundepression könnte eine Immunparalyse verhindert werden welche nachweislich mit einem signifikant erhöhten Auftreten infektionsbedingter Komplikationen verbunden ist [7]. Insbesondere bei großen abdominalchirurgischen Eingriffen wie Pankreas- und Ösophagusresektionen sind postoperative Infektionsraten von bis zu 34 % und eine allgemeine Komplikationsrate von bis zu 48 % beschrieben [30-33]. Eine mögliche Reduktion der Inzidenz postoperativer Infektionen und anderer Komplikationen könnte die Morbidität und Mortalität dieser Patienten nachhaltig senken. Eine damit möglicherweise verbundene Verkürzung der intensivstationären Behandlung und Gesamtkrankenhausverweildauer könnte mit einem potentiell besseren Outcome für die Patienten verbunden sein.
Beide Prüfpräparate sind hinsichtlich ihres Wirkungs- und Risikoprofils in einer Vielzahl von Studien untersucht worden (*siehe hierzu bitte auch das vereinfachte IMPD sowie die Tabellarische Zusammenfassung zu den klinischen Studien mit GM-CSF im Kapitel 2.2.2)*. Diese belegen die Sicherheit in der Anwendung am Patienten unter Berücksichtigung der entsprechenden Kontraindikationen.

Die prä, peri- und postoperative Behandlung der Patienten erfolgt nach den Standards der Universitätsklinik für Anästhesiologie mit Schwerpunkt operative Intensivmedizin – Charité Universitätsmedizin Berlin. Durch die additive Behandlung mit einem der Prüfpräparate wird die Standardbehandlung der Patienten in keinster Weise nachteilig beeinflusst.

8 Abbruch und Weiterbehandlung

8.1 Vorzeitiger Studienabbruch eines einzelnen Patienten

Vorzeitiges Ausscheiden eines Patienten aus der Studie (Abbruchkriterien)
Die folgenden Ereignisse können als Begründung angesehen werden:

- Widerruf der Einwilligung
- schwere Krankheitserscheinungen (z.B. anaphylaktischer Schock, Perikarditis, schwere Krämpfe, Vaskulitis, Enzephalomyelitis, Guillain-Barre-Syndrom, etc.) insofern nicht eine andere Ursache (z.B. chirurgische, anästhesiologische oder diagnostische Prozeduren) erkannt wird und die Entblindung des verabreichten Studienmedikamentes notwendig ist oder eine ab diesem Zeitpunkt vorgesehene Gabe von Studienmedikamenten für den Patienten von Nachteil wäre oder der Patient auf andere Art und Weise bezüglich der Krankheitserscheinungen und ihrer Behandlung von einem Studienabbruch profitieren würde.
- Eintritt einer Schwangerschaft
- jede andere Situation, bei der nach Ansicht des Prüfarztes eine weitere Teilnahme an der klinischen Prüfung nicht im besten Interesse des Patienten sein würde
- Umstände für eine therapeutische Intervention, die durch den Prüfplan nicht zugelassen sind
- Nachträgliches Auftreten eines Ausschlusskriteriums
- signifikante Protokollverletzungen (z.B. Verabreichung der Studienmedikamente nicht möglich, Nichtdurchführen der Operation, vorzeitige Entblindung, etc.)

8.2 Vorzeitiger Abbruch der gesamten klinischen Prüfung

Vorzeitiges Studienende / Abbruch der gesamten Studie
Als Gründe für einen vorzeitigen Abbruch der gesamten Studie kommen in Frage:

- medizinische oder ethische Gründe, die die Fortsetzung der Studie gefährden
- Entscheidung der Studienleitung bei unvertretbaren Risiken und Toxizitäten unter Nutzen-Risiko-Abwägung
- deutliche Häufung von den unter 8.1. bezeichneten, einen Studienabbruch bei den Patienten notwendig machenden schweren Krankheitserscheinungen.
- bei unzureichender Rekrutierungsrate
- neue (wissenschaftliche) Erkenntnisse während der Laufzeit der klinischen Prüfung, die die Sicherheit der Studienteilnehmer gefährden können (positive Nutzen-Risiko-Abwägung nicht mehr gegeben)

Aus der bisherigen Datenlage sowie eigenen Vorläuferuntersuchungen ist nicht zu erwarten, dass die Gesamtstudie abgebrochen werden muss.

Entscheidungsgremium
Die Entscheidung über einen eventuellen Abbruch der Gesamtstudie wird durch den verantwortlichen Hauptprüfer getroffen.

8.3 Plan für die Weiterbehandlung und medizinische Betreuung nach Aus-/Abschluss

Weiteres Vorgehen nach dem vorzeitigen Ausscheiden
Grundsätzlich erfolgt auch nach vorzeitigem Ausscheiden wie zuvor die Behandlung nach den Standards der Universitätsklinik für Anästhesiologie mit Schwerpunkt operative Intensivmedizin, Campus Virchow-Klinikum und Campus Charité Mitte, Charité – Universitätsmedizin Berlin.

Spezifische Verfahrensweisen, z.B. nach dem Auftreten von SAEs entnehmen Sie bitte Kapitel 9 zur *Verfahrensweise bei Unerwünschten Ereignissen*.
Bei vorzeitigem Ausscheiden eines Patienten aus den unter 9.1 aufgeführten Gründen ist der Patient in Bezug auf mögliche Nebenwirkungen von Mutagrip und Leukine nachzuuntersuchen.

Weiteres Vorgehen nach Studienabschluss für den einzelnen Patienten
Die Patienten werden nach Studienabschluss wie auch zuvor nach den Standards der Universitätsklinik für Anästhesiologie mit Schwerpunkt operative Intensivmedizin, Campus Virchow-Klinikum und Campus Charité Mitte, Charité – Universitätsmedizin Berlin, weiterbehandelt.

9 Unerwünschte Ereignisse

9.1 Definitionen (nach Richtlinie 2001/20/EG)

Unerwünschtes Ereignis (Adverse Event - AE)

„Jedes schädliche Vorkommnis das einem Patienten oder einem Prüfungsteilnehmer widerfährt, dem ein Arzneimittel verabreicht wurde, und das nicht unbedingt in kausalem Zusammenhang mit dieser Behandlung steht“
Dies können Erkrankungen, Krankheitszeichen oder Symptome sein, die nach Einschluss des Patienten in die Studie eintreten (nach Beginn der Gabe der Prüfpräparate) oder sich verschlechtern.
Die Ausprägung wird wie folgt bewertet:

- gering
- mäßig
- schwer
- ob die Kriterien für ein SAE erfüllt sind

Für jedes Ereignis ist eine Kausalitätsbewertung vorzunehmen:

- kein Zusammenhang
- möglicher Zusammenhang
- wahrscheinlicher Zusammenhang
- sicherer Zusammenhang
- nicht beurteilbar

Schwerwiegendes unerwünschtes Ereignis (Serious Adverse Event - SAE)

Schwerwiegend ist ein unerwünschtes Ereignis, das

- tödlich oder lebensbedrohend,
- eine stationäre Behandlung oder deren Verlängerung erforderlich macht,
- zu einer bleibenden oder schwerwiegenden Behinderung oder Invalidität führt,
- oder eine kongenitale Anomalie oder einen Geburtsfehler zur Folge hat.

Verdachtsfall einer unerwarteten schwerwiegenden Nebenwirkung“ (Serious Unexpected Suspected Adverse Reaction - SUSAR)

Ein Verdachtsfall einer unerwarteten schwerwiegenden Nebenwirkung (Suspected Unexpected Serious Adverse Reaction – SUSAR) liegt vor, wenn schädliche und unbeabsichtigte Begleiterscheinungen mit der Gabe des Arzneimittels in zeitlichem Zusammenhang stehen (auf Spätschäden muss hierbei gesondert geachtet werden) und nach angemessener Recherche andere Ursachen als die Gabe des Arzneimittels für die Begleiterscheinungen ausgeschlossen werden können.

9.2 Beurteilung der Intensität

Leicht: Das unerwünschte Ereignis ist vorübergehend und vom Patienten leicht zu ertragen.

Mäßig: Das unerwünschte Ereignis bereitet dem Patienten Unannehmlichkeiten und behindert ihn bei seinen üblichen Tätigkeiten.

Schwer: Das unerwünschte Ereignis bereitet dem Patienten erhebliche Störungen seiner üblichen Aktivitäten.

9.3 Beurteilung des Kausalzusammenhanges

Für die Beurteilung des Zusammenhangs zwischen der Anwendung des Prüfproduktes und einem AE werden folgende Definitionen verwendet:

Sicher: Eine Reaktion, die einem nachvollziehbaren zeitlichen Ablauf nach der Anwendung des Prüfproduktes folgt oder bei der die Arzneimittelkonzentration in Körpergewebe oder -flüssigkeit gemessen wurde, einem bekannten oder erwarteten Antwortmuster auf das verdächtigte Prüfprodukt folgt und nach Absetzen oder Dosisreduktion verschwindet und bei erneuter Exposition wieder auftritt.

Wahrscheinlich: Eine Reaktion, die einem nachvollziehbaren zeitlichen Ablauf nach der Anwendung des Prüfproduktes folgt, einem bekannten oder erwarteten Antwortmuster auf das verdächtigte Prüfprodukt folgt und nach Absetzen oder Dosisreduktion verschwindet und nicht durch die bekannten Merkmale des klinischen Zustandes des Patienten erklärt werden kann.

Möglich: Eine Reaktion, die einem nachvollziehbaren zeitlichen Ablauf nach der Anwendung des Prüfproduktes folgt, einem bekannten oder erwarteten Antwortmuster auf das verdächtigte Prüfprodukt folgt, die aber leicht auch durch eine Reihe anderer Faktoren hervorgerufen worden sein könnte.

Ohne Zusammenhang: Eine Reaktion, bei der ausreichend Informationen vorliegen für die Annahme, dass kein Zusammenhang mit dem Prüfprodukt besteht.

Nicht beurteilbar: Eine Einschätzung des Zusammenhangs ist nicht möglich.

9.4 Dokumentation von AEs und SAEs

Alle nach Beginn der Gabe der Prüfpräparate aufgetretenen schwerwiegenden unerwünschten Ereignisse (SAEs) sowie alle unerwünschten Ereignisse (AEs) werden dokumentiert, unabhängig davon, ob nach Meinung des Prüfarztes ein ursächlicher Zusammenhang mit dem Prüfmedikament besteht oder nicht. Die Dokumentation umfasst die Art des Ereignisses, Beginn, Dauer, Ausprägung/Schweregrad und Kausalität.

Im Zusammenhang stehende Krankheitszeichen, Symptome und Laborwertveränderungen werden zu einer einzigen Erkrankung zusammengefasst werden. Für die Dokumentation steht der eCRF zur Verfügung. SAEs werden zusätzlich auf einem gesonderten SAE-Bogen dokumentiert.

Labordaten, die außerhalb des Normbereichs liegen, werden vom Prüfarzt hinsichtlich ihrer klinischen Bedeutung bewertet und - bei entsprechender Relevanz - ebenfalls als unerwünschtes Ereignis zu erfasst.
Alle unerwünschten Ereignisse werden bis zum Abklingen oder bis zur Stabilisierung verfolgt.

Ein generelles „Follow-Up" wird nicht durchgeführt. *Mit der Abschlussuntersuchung am 9. postoperativen Tag noch nicht abgeschlossene AEs werden im Rahmen eines „Follow-Up" von 7 Tagen, noch nicht abgeschlossene SAEs im Rahmen einer „Follow-Up von 21 Tagen, nachverfolgt (siehe auch Kapitel 1 und 9.4). Dem Vertreter des Sponsors bleibt es vorbehalten im Einzelfall von den hier festgelegten Follow-Up-Fristen abzuweichen.*

9.5 Meldung von SAEs und von Verdachtsfällen schwerwiegender unerwarteter unerwünschter Nebenwirkungen (SUSARs)

Exkurs: Meldeverpflichtungen und -fristen in klinischen Prüfungen für SAE/SUSARs (nach GCP-VO §§ 12, 13 und Leitlinie ENTR/CT 3

Prüfarzt ➔ Vertreter des Sponsors

Art	Frist	
AE	Ggf. lt. Protokoll	Schriftlicher Bericht (z.B. auch relevante Laborbefunde)
SAE	Unverzüglich (spätestens aber innerhalb von 24 h)	Schriftlicher Bericht (SAE-Bogen); Ausnahmen lt. Protokoll

Vertreter des Sponsors ➔ Behörden / Prüfärzte

Art	Frist	EK	BOB	Prüfärzte
SAE	Auf Anforderung d. Behörden		x	
SUSAR	Einzelfallbericht innerhalb von 15 Tagen	X	x	x
SUSAR (Tod)	Einzelfallbericht in 7 Tage	x	x	x
Folgemeldung	Weitere 8 Tage	X	x	x

Der Prüfarzt unterrichtet den Vertreter des Sponsors unverzüglich (innerhalb 24 Stunden) über das Auftreten eines schwerwiegenden unerwünschten Ereignisses und übermittelt ihm anschließend einen ausführlichen schriftlichen Bericht.
Die Meldung erfolgt telefonisch und per Fax an:

Prof. Dr. Claudia Spies
Universitätsklinik für Anästhesiologie mit Schwerpunkt operative Intensivmedizin
Charité – Universitätsmedizin Berlin,
Charité Campus Mitte
Charitéplatz 1, 10117 Berlin
Charité Campus Virchow Klinikum
Augustenburger Platz 1, 13353 Berlin
Tel.: 030-450 55 11 02
E-mail: claudia.spies@charite.de

Für jedes dieser Ereignisse wird ein Berichtsbogen (Anlage) ausgefüllt und umgehend an die angegebene Adresse weitergeleitet. Wenn die erforderlichen Informationen zu diesem Zeitpunkt nicht verfügbar sind, werden Folgeberichte abgefasst. Bei Todesfällen wird, falls möglich, eine Kopie des Autopsieberichts beigelegt.

Ausnahmenregelung

Im Rahmen dieser Studie sollen die folgenden schwerwiegenden Ereignisse von der Meldepflicht ausgenommen werden:

- Schwerwiegende oder unerwartete Ereignisse, die während des Run-Ins und der Screening-Phase vor Verabreichung der Studienmedikation eintreten.
- Ereignisse, die regelhaft durch die geplante Operation eintreten. Hierzu zählen insbesondere:

    - Gabe von Standardmedikamente im postoperativen klinischen Verlaufs (Standardanalgetika, Standardsedativa, Standardantibiotika, Antiemetika, Antikoagulantia),
    - eine postoperative Nachbeatmung bis zum 1.postoperativen Tag (wird nicht als Adverse Event gewertet),
    - eine nicht-invasive Nachbeatmung mittels BIPAP oder CPAP bis zum 6. postoperativen Tag (wird nicht als Adverse Event gewertet),
    - einmaliger Temperaturanstieg (≥ 38,5°C),
    - PONV (Postoperative Nausea and Vomiting),
    - postoperatives Shivering,
    - postoperative Heiserkeit bis zum 3. postoperativen Tag einschließlich,
    - Schmerzen, die mit einer Standardschmerztherapie behandelt werden können,
    - ein Anstieg von Entzündungsparametern ohne klinische Infektionszeichen welcher im postoperativen Verlauf unvermeidlich ist (wird nicht als Adverse Event gewertet),
    - Ein postoperativer Abfall des Hämoglobins sowie der Thrombozytenzahl im Blut (wird nur als AE angezeigt, wenn der Transfusionsbedarf postoperativ 2 EKs übersteigt oder ein Revision notwendig wird),
    - Anstieg der Retentionswerte, wenn kein Interventionsbedarf besteht (d.h. forcierte Diurese, Nierenersatzverfahren),
    - eine Re-Intubation bis zum 1. postoperativen Tag,
    - eine Kreislaufunterstützung mittels niedrig-dosierter Katecholamine (bis 0,15 µg/kg/min),
    - allgemeine Symptome, wie vermehrte Müdigkeit, Gewichtsverlust, Gewichtszunahme, Übelkeit, Unwohlsein, Leistungsminderung im postoperativen Verlauf

Der Vertreter des Sponsors dokumentiert ausführlich alle ihm von den Prüfärzten mitgeteilten unerwünschten Ereignisse. Diese Aufzeichnungen übermittelt der Sponsor der zuständigen Bundesoberbehörde und den zuständigen Behörden anderer Mitgliedstaaten der EU und

anderen Vertragsstaaten des Abkommens über den EWR, in deren Hoheitsgebiet die Studie durchgeführt wird, auf Anforderung.

Der Vertreter des Sponsors meldet jeden ihm bekannt gewordenen Verdachtsfall einer unerwarteten schwerwiegenden Nebenwirkung (SUSAR) unverzüglich, spätestens aber innerhalb von 15 Tagen nach Bekanntwerden, der zuständigen Ethikkommission, der zuständigen Bundesoberbehörde und den zuständigen Behörden anderer Mitgliedstaaten der EU und anderen Vertragsstaaten des Abkommens über den EWR, in deren Hoheitsgebiet die Studie durchgeführt wird. Weiterhin unterrichtet er alle an der Studie beteiligten Prüfärzte.

Der Vertreter des Sponsors übermittelt bei einem SUSAR, welches zu einem Todesfall geführt hat oder lebensbedrohlich ist, unverzüglich, spätestens aber innerhalb von 7 Tagen nach Bekanntwerden, der zuständigen Ethikkommission, der zuständigen Bundesoberbehörde und den zuständigen Behörden anderer Mitgliedstaaten der EU und anderen Vertragsstaaten des Abkommens über den EWR, in deren Hoheitsgebiet die Studie durchgeführt wird, sowie allen beteiligten Prüfärzten alle für die Bewertung wichtigen Informationen und innerhalb von höchsten 8 weiteren Tagen die weiteren relevanten Informationen.

Der Vertreter des Sponsor unterrichtet unverzüglich, spätestens aber innerhalb von 15 Tagen nach Bekanntwerden, die zuständige Ethikkommission, die zuständige Bundesoberbehörde und die zuständigen Behörden anderer Mitgliedstaaten der EU und anderen Vertragsstaaten des Abkommens über den EWR, in deren Hoheitsgebiet die Studie durchgeführt wird, über jeden Sachverhalt, der eine erneute Überprüfung der Nutzen-Risiko-Bewertung des Prüfpräparates erfordert. Hierzu gehören insbesondere:

- Einzelfallberichte von erwarteten schwerwiegenden Nebenwirkungen mit einem unerwarteten Ausgang
- Erhöhung der Häufigkeit erwarteter schwerwiegender Nebenwirkungen, die als klinisch relevant bewertet wird
- SUSARs, die sich ereigneten, nachdem die betroffene Person die klinische Prüfung bereits beendet hat (....Wochen, ...Monate, ...Jahre nach Studienende/-ausschluss)
- Ereignisse im Zusammenhang mit der Studiendurchführung oder der Entwicklung des Prüfpräparates, die möglicherweise die Sicherheit der betroffenen Personen beeinträchtigen können

Personenbezogene Daten werden vor ihrer Übermittlung immer pseudonymisierter. Vor Meldung eines SUSARs wird die Verblindung für diesen Patienten aufgehoben.

9.6 Ansprechpartner und Verantwortlicher für die Meldungen

Prof. Dr. Claudia Spies
Universitätsklinik für Anästhesiologie mit Schwerpunkt operative Intensivmedizin
Charité – Universitätsmedizin Berlin,
Charité Campus Mitte
Charitéplatz 1, 10117 Berlin
Charité Campus Virchow Klinikum
Augustenburger Platz 1, 13353 Berlin
Tel.: 030 - 450 55 11 02
Fax: 030 - 450 531 911
E-mail: claudia.spies@charite.de
.

9.7 Data Monitoring Committee

Um eine lückenlose Befunddokumentation der klinischen Prüfung zu gewährleisten, wird ein Monitoring durchgeführt, in welchem in regelmäßigen Abständen die Studienprotokolle eingesehen und auf Vollständigkeit sowie Plausibilität überprüft werden.

10 Dokumentation

10.1 Prüfbögen (CRF)

Die Daten werden in einem ( ) CRF dokumentiert. Die Bögen sind mit Kugelschreiber auszufüllen, Bleistifteintragungen sind nicht erlaubt. Korrekturen sind wie folgt vorzunehmen: Der falsche Eintrag wird mit einer einfachen Linie durchgestrichen, die korrekte Information daneben eingetragen und vom Prüfarzt mit Datum paraphiert und ggf. mit Angabe des Grundes der Korrektur versehen. Datenfelder, die wegen fehlender Information nicht ausgefüllt werden können, sind zu kommentieren.
Die Bögen sind zeitnah von Personen auszufüllen, die zur Dokumentation berechtigt sind (definiert in der sogenannten Studienpersonalliste), anschließend vom Prüfarzt zu kontrollieren, mit Datum zu unterschreiben und im Investigator Site File bis zur Datenverarbeitung abzuheften.

Alle Befunde der Laboratoriumsmedizin bei dieser Studie sind geschwärzt und mit Pseudonym zu kennzeichnen, zu validieren und mit Name und Unterschrift des Studienpersonals und aktuellem Datum zu versehen. Zusätzlich muss die Validierung im CRF aufgezeichnet werden. Auf ein externes Monitoring der Laborparameter wird verzichtet.
Alle patientenbezogenen Daten werden in pseudonymisierter Form erfasst. Jeder Patient ist durch eine Patientennummer bzw. ein Pseudonym, die bei der Registrierung zugewiesen werden, unverwechselbar gekennzeichnet. Der Prüfarzt führt eine vertrauliche Patientenliste, in der die Patientennummern mit dem vollen Patientennamen verbunden sind. Zu dieser Liste hat nur das lokale Studienteam und der Monitor Zugriff. Die Originalakten können von Monitoren, Auditoren und Inspektoren eingesehen werden.

10.2 Prüfarztordner

Im Prüfarztordner werden alle studienrelevanten Dokumente gemäß ICH GCP Kapitel 8 im Prüfzentrum abgelegt. Der Prüfarztordner wird im Prüfzentrum, Universitätsklinik für Anästhesiologie mit Schwerpunkt operative Intensivmedizin, Campus Virchow-Klinikum, Mittelalle 2, 4. OG, Raumnummer 4.0431, in einem verschlossenen Stahlschrank aufbewahrt.

10.3 Dokumentation der Studienmedikation (Drug Accountability)

Die Ausgabe und Verblindung der Studienmedikation wird von der Apotheke durchgeführt. Für jeden einzelnen Patienten wird ein Protokoll (Drug Account) über den Verbleib der Studienmedikation ab Annahme von der Apotheke bis zur Vernichtung geführt. Dieses Protokoll (Drug Account) wird im Prüfarztordner abgelegt. Nach der Ausgabe durch die Apotheke werden die Prüfpräparate ohne Zwischenlagerung dem Patienten durch den verantwortlichen Prüfarzt verabreicht. Die Blister (Spritzen und Perfusorspritzen nach Applikation) werden in einem dafür speziell vorgesehenen Behältnis gesammelt bis der Monitor die Drug Accountability überprüft hat. Im Anschluss müssen die Prüfpräparate auf der Station vom Studienpersonal dokumentiert entsorgt werden. Sollte das Prüfpräparat nach Ausgabe durch die Apotheke nicht dem Patienten verabreicht werden, so ist dies im Drug Account zu vermerken. Auch dann erfolgt die Lagerung in dem hierfür speziell vorgesehenen Behältnis. Nach der Drug Accountability-Kontrolle vom Monitor erfolgt für die unbenutzten Prüfpräparate die dokumentierte Vernichtung durch das Studienpersonal.

11 Qualitätsmanagement

Die klinische Untersuchung wird analog den Grundsätzen für die ordnungsgemäße Durchführung der klinischen Prüfung von Arzneimitteln (Bundesanzeiger 1987, 243) und den EG-GCP Richtlinien (International Conference on Harmonization (ICH): Topic E 6: Guideline for Good Clinical Practice, 1996, ICH: Topic E 9: Statistical Principles for Clinical Trials, 1998), durchgeführt.
Um dem geforderten Anspruch gerecht zu werden, Erkenntnisse nach anerkannten wissenschaftlichen Verfahren zu gewinnen, sind Qualitätssicherungssysteme und Vorgehensvorschriften vorgesehen (Quality assurance).
Die Qualitätssicherung des Studienablaufes erfolgt durch:

- Monitoring nach schriftlichen SOPs,
- Compliance-Kontrolle und
- indem erforderliche Prüfplanänderungen der Ethikkommission und dem PEI unverzüglich mitgeteilt werden (Amendments).

11.1 Überwachung des Studienablaufs und der Datenqualität

Als Qualitätsindikatoren für den Studienablauf gelten folgende Kriterien:

- Einhaltung der Rekrutierungsrate
- Einhaltung der Auswahlkriterien
- Einhaltung des Randomisierungsprinzips / der Verblindung
- Einhaltung der protokollgemäßen Behandlung
- Einhaltung der Untersuchungs- und Bewertungstermine

11.2 Monitoring

Die Clinical Research Organisation (CRO) der Universitätsklinik für Anästhesiologie mit Schwerpunkt operative Intensivmedizin beschäftigt geschultes Fachpersonal zur Durchführung eines unabhängigen klinischen Monitorings und wird die Überwachung dieser Studie vornehmen.

Das Monitoring soll bei 40 % der Patienten und etwa 40 % der Parameter durchgeführt werden. Dazu wird der Monitorin kompletter Zugang zu allen notwendigen Daten gewährt werden. Die Aufgaben der Monitorin beinhalten, dass sie in Form und Inhalt die gemessenen Daten und Charakteristika überprüft und den korrekten Transfer von der Patientenakte in das CRF verifiziert.
Zur Initiierung der Studie, nach Einschluss der ersten fünf Patienten, nach 20 Patienten und nach Abschluss des Einschlusses der Studienpatienten erfolgt ein Monitoring der Studie.

12 Dateneingabe und Datenmanagement

Alle Patienten erhalten nach ihrer Einwilligung in die Studienteilnahme ein Pseudonym. Damit werden alle patientenbezogenen Daten in pseudonymisierter Form erfasst.

( )

Das Pseudonym generiert sich aus Screening-Code und Randomisierungs-Nummer:

Screening-Nr.	Random-Nr	Pseudonym
AAA	1	**AAA1**

Der Prüfarzt führt eine vertrauliche Patientenidentifikationsliste, in der die Pseudonyme mit dem vollen Patientennamen verbunden sind. Jeder Patient ist durch die Pseudonymisierung unverwechselbar gekennzeichnet. Zu dieser Liste hat nur das lokale Studienteam und der Monitor Zugriff. Die Quelldokumente sowie der ()CRF können von Monitoren, Auditoren und Inspektoren eingesehen werden.

12.1 Datenerhebung / Dokumentationsbögen

Die Datenerhebung erfolgt anhand von Prüfbögen (Dokumentationsbögen / Case Report Forms), die von der Studienleitung zur Verfügung gestellt werden.

12.2 Datenverarbeitung

In der Universitätsklinik für Anästhesiologie mit Schwerpunkt operative Intensivmedizin der Charité – Universitätsmedizin Berlin werden die Daten per EDV erfasst. Die Überprüfung der Richtigkeit der Daten erfolgt durch Range-, Validitäts- und Konsistenzchecks. Nicht plausible oder fehlende Daten können nach Rücksprache mit dem Prüfarzt korrigiert bzw. ergänzt werden. Die Korrekturbelege werden zusammen mit den Prüfbogen aufbewahrt.
Die validierten Daten werden in einer elektronischen Datenbank abgelegt. Am Studienende wird nach Eingabe aller Eintragungen die Datenbank geschlossen. Dieser Vorgang wird dokumentiert.

Als Statistik-Software wird IBM SPSS Statistics, in aktueller Version, Inc., Chicago, Illinois 60606, USA eingesetzt.

12.3 Generierung des Pseudonyms

Bei der Bildung des Pseudonyms folgt der Bezeichnung von 3 Screening-DUMMY-Buchstaben bevor sich die fortlaufende Randomnummer anschließt.

z.B. für den ersten Patienten mit den folgenden Daten:
Max Mustermann, geboren 15.Mai.1967
Das Pseudonym wird sein: AAA 1

Z.B. für die zweite Patientin mit den folgenden Daten:
Eva Meyer, geboren 17.Juni.1957
Das Pseudonym wird sein: AAB 2

13 Statistische Analyse

Die biometrische Planung und die statistische Auswertung der Pilotstudie erfolgt in Kooperation mit Prof. Dr. K.-D. Wernecke, Charité – Universitätsmedizin Berlin und SOSTANA GmbH.

Für die Abfassung des statistischen Teils des Protokolls wird besonders auf die einschlägigen internationalen Guidelines verwiesen, insbesondere auf folgende Guidelines der International Conference on Harmonisation (ICH):

- ICH E3: Structure and Contents of Clinical Study Reports
- ICH E6: Good Clinical Practice (GCP). Consolidated Guideline
- ICH E9: Note for Guidance on Statistical Principles in Clinical Trials

Wichtige Aspekte werden nachfolgend zusammengestellt.

13.1 Fallzahlschätzung

Die festgelegte Fallzahl von je 20 Patienten für alle drei Studienarme (N = 60) orientiert sich ausschließlich an den Gegebenheiten der Klinik sowie den Struktureigenschaften der primären Zielgröße und wurde nicht statistisch geplant (siehe auch Kapitel 3.3 zum *Studiendesign*).

13.2 Randomisierung

Die Randomisierung in die vorgesehenen Behandlungsarme (vgl. Studiendesign) erfolgt in Form von Blockbildung. Die Randomisierungsliste wird durch den Studienkoordinator (Biometriker) verwaltet. Der Studienkoordinator übergibt der Apotheke eine Liste mit den Randomisierungsnummern und der jeweiligen Zuteilung zu einer der drei Behandlungen. Weder dem behandelnden Arzt noch dem Patienten ist diese Liste zugänglich, aus der Verpackung bzw. Verabreichung der Medikation ist ebenso wenig eine Information über die Art der Therapie zu entnehmen. Bei Einschluss des Patienten in die Studie wird ihm die laufende Randomisierungsnummer zugeordnet. Der Patient behält diese Nummer während der gesamten Prüfung. Wenn die klinische Prüfung bei einem Patienten aus medizinischen oder anderen Gründen abgebrochen werden muss, wird dafür kein neuer Patient in die Prüfung aufgenommen (keine Berücksichtigung von „Drop-outs“).

13.3 Statistische Auswertung

13.3.1 Hypothesen

Gegenstand der Primärhypothese ist, ob eine postoperative Vakzinierung mit Influenza-Impfstoff (Verum 1) oder eine postoperative Behandlung mit Sargramostim (Verum 2) im Stadium der schweren Immundepression, im Vergleich zu Placebo, zu einer Verbesserung der Immunreaktivität bei Ausbleiben einer Immunparalyse, gemessen anhand der Normalisierung der monozytären HLA-DR Expression (Antigene/Monozyt), führt.

H_0 (Nullhypothese): |HLA-DR-Expression (Verum 1) – HLA-DR-Expression (Placebo)| = 0
H_A (Alternativhypothese):|HLA-DR-Expression (Verum 1) – HLA-DR-Expression (Placebo)| ≠ 0

H_0 (Nullhypothese): |HLA-DR-Expression (Verum 2) – HLA-DR-Expression (Placebo)| = 0
H_A (Alternativhypothese):|HLA-DR-Expression (Verum 2) – HLA-DR-Expression (Placebo)| ≠ 0

H_0 (Nullhypothese): |HLA-DR-Expression (Verum 1) – HLA-DR-Expression (Verum 2)| = 0
H_A (Alternativhypothese):|HLA-DR-Expression (Verum 1) – HLA-DR-Expression (Verum 2)| ≠ 0

13.3.2 Definition der Auswertungspopulation

Die Analyse bezüglich der Haupt- und Nebenzielkriterien erfolgt sowohl gemäß einer Intent-to-treat (ITT) bzw. Full-Analysis-Set (FAS) als auch einer Per-protocol (PP)- Strategie (Valid-Case-Kollektiv). Die Resultate werden präsentiert und miteinander verglichen.

Intent-to-treat-Population

In die ITT-Stichprobe gehen alle Patienten ein, die

- in einen Behandlungsarm randomisiert wurden und
- mindestens einen protokollgerechten Behandlungstag erfahren haben.

Falls in der ITT-Population bei einem Patienten das primäre Zielkriterium nicht bestimmt werden kann, so wird dieser im Rahmen eines Worst-Case-Szenarios als Patient ohne klinischen Benefit gewertet.

Per-protocol-Population

In die Valid-Case-Stichprobe (PP -Kollektiv) gehen alle Patienten ein, die

- in der ITT-Stichprobe analysiert wurden
- protokollgemäß therapiert wurden
- bei den Ausschlusskriterien nur sicherheitsrelevante Parameter verletzen.

Population für Sicherheitsanalyse

Bezüglich der Sicherheitsanalyse gelten alle Patienten, die mindestens einen Behandlungstag erfahren haben, als auswertbar bezüglich der Toxizität. Die Prüfärzte sind für die Überwachung der Sicherheit der Patienten, die in die Studie aufgenommen werden, verantwortlich.

()

13.3.3 Auswertung primärer und sekundärer Zielparameter

Primäre Zielgröße: HLA-DR-Expression auf Monozyten

Sekundäre Zielgrößen:

- Postoperative Infektionsrate
- Postoperative Delirrate (CAM-ICU, NuDESC, DDS)
- Beatmungsstunden, ITS-Liegedauer, Charité-Liegedauer, APACHE-II-Score, SAPS-II-Score, SOFA-Score, TISS-28-Score
- Die ersten 33 eingeschlossenen Patienten der Behandlungsgruppen werden nach folgenden Parametern ausgewertet:

1. Zytokinproduktion im Serum, das Th_1/Th_2-Verhältniss, das Th_{17}/T_{reg}-Verhältnis, andere Immunfunktionsparameter
2. Quantitative Expression von Transkriptionsfaktoren (T-bet, Eomesodermin, GATA-3, Foxp3, RORγt, PU.1) und Proteinen (SOCS-3, SOCS-1, TGF-ß, IL-17) sowie die Synthese der entsprechenden Effektorproteinen

Für Informationen zu den oben beschriebenen primären und sekundären Zielgrößen wird auf die entsprechenden erklärenden Kapitel verwiesen.

13.3.4 Auswertung der Sicherheit

Eine Sicherheitsanalyse ist bei den Behandlungsgruppen vorzusehen, unabhängig davon, wie die Hauptzielsetzung der Studie definiert ist. Es erfolgt mindestens eine Evaluation getrennt für

jeden Therapiearm bzgl. der Anzahl unerwünschten Ereignisse. Desweiteren wird auf den jährlich zu erstellenden Sicherheitsbericht sowie auf den Abschlussbericht verwiesen.

13.3.5 Mögliche Zwischenauswertungen

Es ist keine Zwischenauswertung vorgesehen.

13.3.6 Auswertungsmethodik

Für alle Zielgrößen werden die Befunde ausschließlich exploratorisch untersucht und deskriptiv ausgewertet, d.h. statistische Maßzahlen wie Mittelwert und Varianz (metrische Merkmale), Median und Interquartildifferenz (ordinale Merkmale) sowie erreichte Häufigkeiten bei nominalen Merkmalen bestimmt. Die erhaltenen Ergebnisse dienen zur Planung einer nachfolgenden prospektiven kontrollierten klinischen Prüfung mit entsprechender statistischer Planung der Fallzahl.
Möglicherweise durchgeführte statistische Test zur Prüfung der unter 13.3.1 aufgestellten Hypothesen (und weiterer) sind ausschließlich explorativer Natur, d.h. können interessante Hinweise für die wissenschaftliche Fragestellung der Studie bedeuten, sind aber nicht im konfirmatorischen Sinne zu interpretieren bzw. zu verallgemeinern. Aus dem gleichen Grunde erfolgt keine Adjustierung des Fehlers 1. Art, falls mehrfach getestet wird.
Die Auswertungen sind jeweils nach möglichen Einflussfaktoren stratifiziert durchzuführen, darüber hinaus ist eine getrennte Analyse für einzelne Strata mit einem Vergleich der Ergebnisse vorgesehen.

Die statistische Analyse für metrisch-skalierte Daten erfolgt mittels Mann-Whitney U-Test (für unabhängige Gruppen bzw. Wilcoxon-Test (für abhängige Gruppen). Bei nominal skalierten klinischen Daten werden die Unterschiede mit Hilfe des Chi-Quadrat-Tests auf Signifikanz untersucht.

Für die statistische Prüfung zur Erkennung von Unterschieden bezüglich der zeitlichen Immunparameterverläufe zwischen den Gruppen (Influenza-Impfung versus Placebo, Sargramostim versus Placebo, Influenza-Impfung versus Sargramostim) und Wechselwirkungen zwischen Zeitverlauf und Gruppe sowie zur Analyse des Zeitverlaufes innerhalb der Gruppen wird die nichtparametrische Varianzanalyse für wiederholte Messungen angewandt.
Die statistische Auswertung der Zusammenhänge der untersuchten Parameter mit der Infektionsinzidenz erfolgt für alle Gruppen getrennt durch Receiver Operating Characteristics (ROCs), Rang Korrelation nach Spearman oder mittels Abhängigkeitsprüfung mit dem Chi-Quadrat-Test. Ein $p < 0,5$ wird als statistisch signifikant betrachtet.

14 Berichterstattung

14.1 Biometrischer Bericht

Die statistische Auswertung und die Erstellung des biometrischen Berichts wird durch Prof. Dr. K.-D. Wernecke, Charité – Universitätsmedizin Berlin und SOSTANA GmbH, in Zusammenarbeit mit dem Vertreter des Sponsors / Hauptprüfer der klinischen Prüfung durchgeführt. Der Dritte, die SOSTANA GmbH erhält nur pseudonymisierte Daten. Alle in diesem Bericht enthaltenen Informationen sind vertraulich.

14.2 Abschlussbericht

Nach Abschluss der biometrischen Auswertung wird ein integrierter Bericht von Univ.-Prof. Dr. Claudia Spies in Zusammenarbeit mit dem Studienkoordinator Prof. Dr. K.-D. Wernecke und den Prüfärzten erstellt.
Der an die Ethikkommission und das PEI zu sendende Abschlussbericht enthält den Sicherheitsbericht, eine Synopsis und das publizierte Paper.

Es ist sichergestellt, dass der im Protokoll benannte Biometriker Prof. Dr. K.-D. Wernecke (siehe Verantwortlichkeiten) die Überwachung der statistischen Auswertung wahrnehmen kann durch die Sostana GmbH.

14.3 Publikation

Die Veröffentlichung der Studienergebnisse erfolgt unabhängig davon, wie die Ergebnisse ausfallen. Die Präsentation der Ergebnisse im Rahmen einer Publikation wird den im CONSORT-Statement (www.consort-statement.org) festgelegten Umfang entsprechen. Diese Studie wird vor Einschluss des ersten Patienten in einer öffentlich zugänglichen Datenbank (z. B. www.controlled-trials.com) registriert werden, um nach Vorliegen der Studienergebnisse diese in renommierten Fachzeitschriften veröffentlichen zu können.
Die Veröffentlichung oder ein Vortrag der Ergebnisse bedürfen einer vorhergehenden Kommentierung und Genehmigung durch den Hauptprüfer der klinischen Prüfung Frau Univ.-Prof. Dr. C. Spies. Für alle Veröffentlichungen gilt, dass der Datenschutz für alle Patientendaten gewahrt bleibt.
Mit ihrer Unterschrift unter dem Prüfplan erklärt der Hauptprüfer sein Einverständnis, dass die Ergebnisse dieser klinischen Prüfung bei nationalen und internationalen Überwachungsbehörden vorgelegt werden. Gleichzeitig erklären sich die Prüfer damit einverstanden, dass in diesem Zusammenhang ihre Namen, Anschriften, Qualifikationsmerkmale und der Umfang ihrer Beteiligung an der klinischen Prüfung bekannt gegeben werden.

15 Ethische, rechtliche und verwaltungstechnische Aspekte

15.1 Rechtliche Voraussetzungen für die Studie

<u>Votum der Ethikkommission (gem. AMG § 42 (1) und GCP-V § 7)</u>

Protokoll, Patienteninformation und Einwilligungserklärung werden der (für den Hauptprüfer zuständigen) Ethikkommission (Bezeichnung der Kommission) zur Begutachtung vorgelegt. Die Studie wird erst nach Erhalt des zustimmenden Votums begonnen.
Die Ethikkommission wird (vom Vertreter des Sponsors) über alle Änderungen im Protokoll (gem. GCP-V § 10) und über alle Ereignisse, die die Sicherheit der Patienten beeinträchtigen könnten, umgehend informiert. Ferner wird die Ethikkommission über alle dem Vertreter des Sponsors bekannt gewordene Verdachtsfälle von unerwarteten schwerwiegenden Nebenwirkungen sowie über das reguläre oder vorzeitige Ende der Studie unterrichtet.
Die Prüfärzte sind verpflichtet, sich bei der für sie zuständigen Ethikkommission anzumelden (Einreichen der Qualifikationsnachweise), bevor sie Patienten in die Studie aufnehmen. Es ist erforderlich, die Ethikkommission über Protokolländerungen (gem. GCP-V § 10) zu informieren.

Genehmigung der Bundesoberbehörde (gem. AMG § 42 (2) und GCP-V § 7)

Die Studie wird der zuständigen Bundesoberbehörde (PEI) zur Genehmigung vorgelegt. Mit der Studie wird erst dann begonnen, wenn diese Genehmigung vorliegt.

Meldung bei den Landesbehörden (gem. AMG § 67)

Die Durchführung dieser Studie wird der zuständigen Behörde (Landesamt für Gesundheit und Soziales Berlin) gemeldet. Der Sponsor und sämtliche Prüfärzte sind dort namentlich genannt.

Patienteninformation und Einverständniserklärung

Aufklärung der Patienten

Vor Aufnahme in die Studie (z.B. Randomisierung) wird jeder Patient vom behandelnden Arzt über Wesen, Ziele, erwartete Vorteile und mögliche Risiken der Studie mündlich und schriftlich aufgeklärt.

Einwilligung zur Studienteilnahme

Jeder Patient muss seine schriftliche Einwilligung zur Teilnahme an der Studie erklären. Dem Patienten muss dabei ausreichend Zeit und Gelegenheit gegeben werden, um vor der Einleitung von Studienmaßnahmen über seine Teilnahme zu entscheiden und offene Fragen zu klären.

Die Einwilligungserklärung wird vom Patienten und vom behandelnden Arzt unterzeichnet und eigenhändig datiert. Ist der Patient einwilligungsfähig aber nicht in der Lage, eigenhändig zu unterschreiben, muss ein Zeuge die erfolgte mündliche Aufklärung durch Unterschrift bestätigen.

Ein Muster der Patienteninformationen und der Einwilligungserklärung sind in den Appendices (Nr. 1, 2 und 3) beigefügt. Äußere Form und Sprache sind den Gepflogenheiten des Prüfzentrums anzupassen. Auf Anforderung sind die endgültigen Formblätter der zuständigen Ethikkommission zur Begutachtung vorzulegen.

Die Patienteninformationen sowie die Einwilligungserklärung liegen in zweifacher Ausfertigung vor. Ein Exemplar verbleibt beim Prüfarzt, das andere ist dem Patienten auszuhändigen.

Probandenversicherung

Für diese vorliegende klinische Studie ist eine Versicherung (gem. AMG § 40 Absatz 1 Satz 3 Nr. 8) abgeschlossen worden.

Für die Studie besteht seit 05.07.2007 Versicherungsschutz bei Ecclesia Versicherungsdienst GmbH (Ecclesia Versicherungsdienst GmbH, Klingenbergstrasse 4, 32758 Detmold, Tel.: 05231 / 603- 0, Fax: 05231 / 603-197), Versicherungsschein Nr. 70-5644584-4 bis zum 31.01.2008. Der Umfang der Versicherungssumme pro Patient beträgt 500.000 Euro.

Datenschutz

Die Patienten werden darüber informiert, dass ihre krankheitsbezogenen Daten in pseudonymisierter Form gespeichert und für wissenschaftliche Auswertungen (Publikationen, Zulassungsdossiers) verwendet werden. Die Patienten haben das Recht, über die gespeicherten Daten informiert zu werden. Sie werden auch darüber aufgeklärt, dass ihre pseudonymisierten Daten im Rahmen der gesetzlichen Meldepflichten zur Arzneimittelsicherheit an die zuständigen Bundesoberbehörden, auch in Mitgliedsstaaten der Europäischen Union, in denen die Studie durchgeführt wird, sowie an die zuständigen Ethikkommissionen weitergegeben werden. Patienten, die dieser Weitergabe nicht zustimmen dürfen an der Studie nicht teilnehmen.

15.2 Aufbewahrung der Daten und Zugang zu den Daten

Die Originale aller zentralen Studiendokumente einschließlich Dokumentationsbogen werden in der Studienzentrale (Universitätsklinik für Anästhesiologie mit Schwerpunkt operative Intensivmedizin, Campus Virchow-Klinikum, Mittelallee 8, 1. OG, Raum- Nr.: 1.3816) für mindestens 10 Jahre nach Abschluss der klinischen Prüfung (GCP-V § 13(10)) aufbewahrt.
Der Hauptptüfer des Prüfzentrums bewahrt die angefallenen administrativen Dokumente (Schriftverkehr mit Ethikkommission, Überwachungsbehörde, Studienleitung, Studienzentrale), die Patientenidentifikationsliste, die unterschriebenen Einwilligungserklärungen, Kopien der Dokumentationsbogen und der allgemeinen Studiendokumentation (Protokoll, Amendments) für die oben genannte Zeit auf.
Originaldaten der Studienpatienten (Krankenakten) werden entsprechend der für das Prüfzentrum gültigen Archivierungsfrist, aber nicht weniger als 10 Jahre aufbewahrt.
Es wird garantiert, dass entsprechende Daten sowie Dokumente für den Prüfarzt, Hauptprüfer, der Zulassungsbehörde, der unabhängigen Ethikkommission oder anderen Überwachungsbehörden einsehbar sind. Nicht personengebundene Daten sind in pseudonymisierter Form einsehbar.

16 Literaturverzeichnis

1. Sander M, Irwin M, Sinha P, Naumann E, Kox WJ, Spies CD. Suppression of interleukin-6 to interleukin-10 ratio in chronic alcoholics: association with postoperative infections., *Intensive Care Med.* 2002;28:285-292.

2. Spies CD, Von D, Eggers V, et al.. Altered cell-mediated immunity and increased postoperative infection rate in long-term alcoholic patients., *Anesthesiology.* 2004;100:1088-1100.

3. Docke WD, Hoflich C, Davis KA, et al.. Monitoring temporary immunodepression by flow cytometric measurement of monocytic HLA-DR expression: a multicenter standardized study., *Clin Chem.* 2005;51:2341-2347.

4. Spies CD, Kern H, Schroder T, et al.. Myocardial ischemia and cytokine response are associated with subsequent onset of infections after noncardiac surgery., *Anesth Analg.* 2002;95:9-18, table.

5. Volk HD, Reinke P, Docke WD. Immunological monitoring of the inflammatory process: Which variables? When to assess?, *Eur J Surg Suppl.* 1999:70-72.

6. Allen ML, Peters MJ, Goldman A, et al.. Early postoperative monocyte deactivation predicts systemic inflammation and prolonged stay in pediatric cardiac intensive care., *Crit Care Med.* 2002;30:1140-1145.

7. Strohmeyer JC, Blume C, Meisel C, et al.. Standardized immune monitoring for the prediction of infections after cardiopulmonary bypass surgery in risk patients., *Cytometry B Clin Cytom.* 2003;53:54-62.

8. Berguer R, Bravo N, Bowyer M, Egan C, Knolmayer T, Ferrick D. Major surgery suppresses maximal production of helper T-cell type 1 cytokines without potentiating the release of helper T-cell type 2 cytokines., *Arch Surg.* 1999;134:540-544.

9. Brune IB, Wilke W, Hensler T, Holzmann B, Siewert JR. Downregulation of T helper type 1 immune response and altered pro-inflammatory and anti-inflammatory T cell cytokine balance following conventional but not laparoscopic surgery., *Am J Surg.* 1999;177:55-60.

10. Decker D, Schondorf M, Bidlingmaier F, Hirner A, von Ruecker AA. Surgical stress induces a shift in the type-1/type-2 T-helper cell balance, suggesting down-regulation of cell-mediated and up-regulation of antibody-mediated immunity commensurate to the trauma., *Surgery.* 1996;119:316-325.

11. Near KA, Stowers AW, Jankovic D, Kaslow DC. Improved immunogenicity and efficacy of the recombinant 19-kilodalton merozoite surface protein 1 by the addition of oligodeoxynucleotide and aluminum hydroxide gel in a murine malaria vaccine model., *Infect Immun.* 2002;70:692-701.

12. Jones HP, Hodge LM, Fujihashi K, Kiyono H, McGhee JR, Simecka JW. The pulmonary environment promotes Th2 cell responses after nasal-pulmonary immunization with antigen alone, but Th1 responses are induced during instances of intense immune stimulation., *J Immunol.* 2001;167:4518-4526.

13. Gorse GJ, Patel GB, Belshe RB. HIV type 1 vaccine-induced T cell memory and cytotoxic T lymphocyte responses in HIV type 1-uninfected volunteers., *AIDS Res Hum Retroviruses.* 2001;17:1175-1189.

14. Gorse GJ, Corey L, Patel GB, et al.. HIV-1MN recombinant glycoprotein 160 vaccine-induced cellular and humoral immunity boosted by HIV-1MN recombinant glycoprotein 120 vaccine. National Institute of Allergy and Infectious Diseases AIDS Vaccine Evaluation Group., *AIDS Res Hum Retroviruses.* 1999;15:115-132.

15. Evans TG, Fitzgerald T, Gibbons DC, Keefer MC, Soucier H. Th1/Th2 cytokine responses following HIV-1 immunization in seronegative volunteers. The AIDS Vaccine Evaluation Group., *Clin Exp Immunol.* 1998;111:243-250.

16. Blazevic V, Trubey CM, Shearer GM. Comparison of in vitro immunostimulatory potential of live and inactivated influenza viruses., *Hum Immunol.* 2000;61:845-849.

17. Spies, C., Kip, M., Lau, A., Sander, M., Breuer, J. P., Meyerhoefer, J., et al. (in press). Influence of vaccination and surgery on HLA-DR expression in patients with upper aerodigestive tract cancer. *J Int Med Res*.

18. Reed SG, Nathan CF, Pihl DL, et al.. Recombinant granulocyte/macrophage colony-stimulating factor activates macrophages to inhibit Trypanosoma cruzi and release hydrogen peroxide. Comparison with interferon gamma., *J Exp Med.* 1987;166:1734-1746.

19. Kurt-Jones EA, Mandell L, Whitney C, et al.. Role of toll-like receptor 2 (TLR2) in neutrophil activation: GM-CSF enhances TLR2 expression and TLR2-mediated interleukin 8 responses in neutrophils., *Blood.* 2002;100:1860-1868.

20. Schneider C, von Aulock S, Zedler S, Schinkel C, Hartung T, Faist E. Perioperative recombinant human granulocyte colony-stimulating factor (Filgrastim) treatment prevents immunoinflammatory dysfunction associated with major surgery., *Ann Surg.* 2004;239:75-81.

21. Hartung T, Von Aulock S, Schneider C, Faist E. How to leverage an endogenous immune defense mechanism: the example of granulocyte colony-stimulating factor., *Crit Care Med.* 2003;31:S65-S75.

22. Nierhaus A, Montag B, Timmler N, et al.. Reversal of immunoparalysis by recombinant human granulocyte-macrophage colony-stimulating factor in patients with severe sepsis., *Intensive Care Med.* 2003;29:646-651.

23. Rosenbloom AJ, Linden PK, Dorrance A, Penkosky N, Cohen-Melamed MH, Pinsky MR. Effect of granulocyte-monocyte colony-stimulating factor therapy on leukocyte function and clearance of serious infection in nonneutropenic patients., *Chest.* 2005;127:2139-2150.

24. Borgermann J, Friedrich I, Scheubel R, et al.. Granulocyte-macrophage colony-stimulating factor (GM-CSF) restores decreased monocyte HLA-DR expression after cardiopulmonary bypass., *Thorac Cardiovasc Surg.* 2007;55:24-31.

25. Flohe S, Borgermann J, Dominguez FE, et al.. Influence of granulocyte-macrophage colony-stimulating factor (GM-CSF) on whole blood endotoxin responsiveness following trauma, cardiopulmonary bypass, and severe sepsis., *Shock.* 1999;12:17-24.

26. Drossou-Agakidou V, Kanakoudi-Tsakalidou F, Sarafidis K, et al.. In vivo effect of rhGM-CSF And rhG-CSF on monocyte HLA-DR expression of septic neonates., *Cytokine.* 2002;18:260-265.

27. Govaert TM, Dinant GJ, Aretz K, Masurel N, Sprenger MJ, Knottnerus JA. Adverse reactions to influenza vaccine in elderly people: randomised double blind placebo controlled trial., *BMJ.* 1993;307:988-990.

28. Margolis KL, Poland GA, Nichol KL, et al.. Frequency of adverse reactions after influenza vaccination., *Am J Med.* 1990;88:27-30.

29. Margolis KL, Nichol KL, Poland GA, Pluhar RE. Frequency of adverse reactions to influenza vaccine in the elderly. A randomized, placebo-controlled trial., *JAMA.* 1990;264:1139-1141.

30. Bachellier P, Nakano H, Oussoultzoglou PD, et al.. Is pancreaticoduodenectomy with mesentericoportal venous resection safe and worthwhile?, *Am J Surg.* 2001;182:120-129.

31. Nguyen TC, Sohn TA, Cameron JL, et al.. Standard vs. radical pancreaticoduodenectomy for periampullary adenocarcinoma: a prospective, randomized trial evaluating quality of life in pancreaticoduodenectomy survivors., *J Gastrointest Surg.* 2003;7:1-9; discussion 9-11.

32. Riediger H, Adam U, Fischer E, et al.. Long-term Outcome After Resection for Chronic Pancreatitis in 224 Patients., *J Gastrointest Surg.* 2007.

33. Yeo CJ, Cameron JL, Sohn TA, et al.. Pancreaticoduodenectomy with or without extended retroperitoneal lymphadenectomy for periampullary adenocarcinoma: comparison of morbidity and mortality and short-term outcome., *Ann Surg.* 1999;229:613-22; discussion 622-4.

34. Underhill DM, Ozinsky A, Smith KD, Aderem A. Toll-like receptor-2 mediates mycobacteria-induced proinflammatory signaling in macrophages., *Proc Natl Acad Sci U S A.* 1999;96:14459-14463.

35. Takeuchi O, Hoshino K, Kawai T, et al.. Differential roles of TLR2 and TLR4 in recognition of gram-negative and gram-positive bacterial cell wall components., *Immunity.* 1999;11:443-451.

36. Poltorak A, Smirnova I, He X, et al.. Genetic and physical mapping of the Lps locus: identification of the toll-4 receptor as a candidate gene in the critical region., *Blood Cells Mol Dis.* 1998;24:340-355.

37. Poltorak A, He X, Smirnova I, et al.. Defective LPS signaling in C3H/HeJ and C57BL/10ScCr mice: mutations in Tlr4 gene., *Science.* 1998;282:2085-2088.

38. (Beutler B.). Innate immunity: an overview., *Mol Immunol.* 2004;40:845-859.

39. Lorenz E, Mira JP, Cornish KL, Arbour NC, Schwartz DA. A novel polymorphism in the toll-like receptor 2 gene and its potential association with staphylococcal infection., *Infect Immun.* 2000;68:6398-6401.

40. Agnese DM, Calvano JE, Hahm SJ, et al.. Human toll-like receptor 4 mutations but not CD14 polymorphisms are associated with an increased risk of gram-negative infections., *J Infect Dis.* 2002;186:1522-1525.

41. Kang TJ, Lee SB, Chae GT. A polymorphism in the toll-like receptor 2 is associated with IL-12 production from monocyte in lepromatous leprosy., *Cytokine.* 2002;20:56-62.

42. Bornstein SR, Schumann RR, Rettori V, McCann SM, Zacharowski K. Toll-like receptor 2 and Toll-like receptor 4 expression in human adrenals., *Horm Metab Res.* 2004;36:470-473.

43. Epidemiologisches Bulletin 2007. *Empfehlung der Ständigen Impfkommission (STIKO).* Ständige Impkommission am Robert-Koch Institut.

44. Keitel WA, Atmar RL, Cate TR, et al.. Safety of high doses of influenza vaccine and effect on antibody responses in elderly persons., *Arch Intern Med.* 2006;166:1121-1127.

45. Bober LA, Grace MJ, Pugliese-Sivo C, et al.. The effect of GM-CSF and G-CSF on human neutrophil function., *Immunopharmacology.* 1995;29:111-119.

46. (Kellihan MJ.). Drug formulary review process for sargramostim and filgrastim: focus on analysis of adverse drug reactions., *Clin Ther.* 1993;15:927-937.

47. Winter JN, Lazarus HM, Rademaker A, et al.. Phase I/II study of combined granulocyte colony-stimulating factor and granulocyte-macrophage colony-stimulating factor administration for the mobilization of hematopoietic progenitor cells., *J Clin Oncol.* 1996;14:277-286.

48. Meropol NJ, Petrelli NJ, Lipman BJ, et al.. Granulocyte-macrophage colony-stimulating factor as infection prophylaxis in high-risk oncologic surgery., *Am J Surg.* 1996;172:299-302.

49. North JH,, Weber TK, Rodriguez-Bigas MA, Meropol NJ, Petrelli NJ. The management of infectious and noninfectious anorectal complications in patients with leukemia., *J Am Coll Surg.* 1996;183:322-328.

50. Schaefer H, Engert A, Grass G, et al.. Perioperative granulocyte colony-stimulating factor does not prevent severe infections in patients undergoing esophagectomy for esophageal cancer: a randomized placebo-controlled clinical trial., *Ann Surg.* 2004;240:68-75.

51. Schafer H, Hubel K, Bohlen H, et al.. Perioperative treatment with filgrastim stimulates granulocyte function and reduces infectious complications after esophagectomy., *Ann Hematol.* 2000;79:143-151.

52. Weiss M, Elsharkawi M, Welt K, Schneider EM. Transient leukocytosis, granulocyte colony-stimulating factor plasma concentrations, and apoptosis determined by binding of annexin V by peripheral leukocytes in patients with severe sepsis., *Ann N Y Acad Sci.* 2003;1010:742-747.

53. Weiss M, Voglic S, Harms-Schirra B, et al.. Effects of exogenous recombinant human granulocyte colony-stimulating factor (filgrastim, rhG-CSF) on neutrophils of critically ill patients with systemic inflammatory response syndrome depend on endogenous G-CSF plasma concentrations on admission., *Intensive Care Med.* 2003;29:904-914.

54. Pilla L, Patuzzo R, Rivoltini L, et al.. A phase II trial of vaccination with autologous, tumor-derived heat-shock protein peptide complexes Gp96, in combination with GM-CSF and interferon-alpha in metastatic melanoma patients., *Cancer Immunol Immunother.* 2006;55:958-968.

55. Heard SO, Fink MP, Gamelli RL, et al.. Effect of prophylactic administration of recombinant human granulocyte colony-stimulating factor (filgrastim) on the frequency of nosocomial infections in patients with acute traumatic brain injury or cerebral hemorrhage. The Filgrastim Study Group., *Crit Care Med.* 1998;26:748-754.

56. Ahmad A, Laborada G, Bussel J, Nesin M. Comparison of recombinant granulocyte colony-stimulating factor, recombinant human granulocyte-macrophage colony-stimulating factor and placebo for treatment of septic preterm infants., *Pediatr Infect Dis J.* 2002;21:1061-1065.

57. Apte SM, Vadhan-Raj S, Cohen L, et al.. Cytokines, GM-CSF and IFNgamma administered by priming and post-chemotherapy cycling in recurrent ovarian cancer patients receiving carboplatin., *J Transl Med.* 2006;4:16.

58. Atzpodien J, Reitz M. GM-CSF plus antigenic peptide vaccination in locally advanced melanoma patients., *Cancer Biother Radiopharm.* 2007;22:551-555.

59. Barrio MM, de Motta PT, Kaplan J, et al.. A phase I study of an allogeneic cell vaccine (VACCIMEL) with GM-CSF in melanoma patients., *J Immunother (1997).* 2006;29:444-454.

60. Bilgin K, Yaramis A, Haspolat K, Tas MA, Gunbey S, Derman O. A randomized trial of granulocyte-macrophage colony-stimulating factor in neonates with sepsis and neutropenia., *Pediatrics.* 2001;107:36-41.

61. Bruno S, Bussolati B, Scacciatella P, et al.. Combined administration of G-CSF and GM-CSF stimulates monocyte-derived pro-angiogenic cells in patients with acute myocardial infarction., *Cytokine.* 2006;34:56-65.

62. Bunn PAJ, Crowley J, Kelly K, et al.. Chemoradiotherapy with or without granulocyte-macrophage colony-stimulating factor in the treatment of limited-stage small-cell lung cancer: a prospective phase III randomized study of the Southwest Oncology Group., *J Clin Oncol.* 1995;13:1632-1641.

63. Connor RF, Hurd D, Pettenati MJ, Koty P, Molnár I. Addition of sargramostim (GM-CSF) to imatinib results in major cytogenetic response in a patient with chronic myeloid leukemia., *Leuk Res.* 2006;30:1249-1252.

64. Deng Z, Yang C, Deng H, et al.. Effects of GM-CSF on the stem cells mobilization and plasma C-reactive protein levels in patients with acute myocardial infarction., *Int J Cardiol.* 2006;113:92-96.

65. Hasskamp JH, Elias EG, Zapas JL. In vivo effects of sequential granulocyte-macrophage colony stimulating factor (GM-CSF) and interleukin-2 (IL-2) on circulating dendritic cells (DC) in patients with surgically resected high risk cutaneous melanoma., *J Clin Immunol.* 2006;26:331-338.

66. Hueman MT, Stojadinovic A, Storrer CE, et al.. Levels of circulating regulatory CD4+CD25+ T cells are decreased in breast cancer patients after vaccination with a HER2/neu peptide (E75) and GM-CSF vaccine., *Breast Cancer Res Treat.* 2006;98:17-29.

67. Hueman MT, Stojadinovic A, Storrer CE, et al.. Analysis of naïve and memory CD4 and CD8 T cell populations in breast cancer patients receiving a HER2/neu peptide (E75) and GM-CSF vaccine., *Cancer Immunol Immunother.* 2007;56:135-146.

68. Kirman I, Belizon A, Balik E, et al.. Perioperative sargramostim (recombinant human GM-CSF) induces an increase in the level of soluble VEGFR1 in colon cancer patients undergoing minimally invasive surgery., *Eur J Surg Oncol.* 2007;33:1169-1176.

69. Korzenik JR, Dieckgraefe BK, Valentine JF, Hausman DF, Gilbert MJ. Sargramostim for active Crohn's disease., *N Engl J Med.* 2005;352:2193-2201.
70. Liljefors M, Nilsson B, Mellstedt H, Frodin JE. Influence of varying doses of granulocyte-macrophage colony-stimulating factor on pharmacokinetics and antibody-dependent cellular cytotoxicity., *Cancer Immunol Immunother.* 2007.

71. McAleese JJ, Bishop KM, A'Hern R, Henk JM. Randomized phase II study of GM-CSF to reduce mucositis caused by accelerated radiotherapy of laryngeal cancer., *Br J Radiol.* 2006;79:608-613.

72. Mels AK, Statius Muller MG, van Leeuwen PA, et al.. Immune-stimulating effects of low-dose perioperative recombinant granulocyte-macrophage colony-stimulating factor in patients operated on for primary colorectal carcinoma., *Br J Surg.* 2001;88:539-544.

73. Meropol NJ, Wood DE, Nemunaitis J, et al.. Randomized, placebo-controlled, multicenter trial of granulocyte-macrophage colony-stimulating factor as infection prophylaxis in oncologic surgery. Leukine Surgical Prophylaxis Research Group., *J Clin Oncol.* 1998;16:1167-1173.

74. Oosterling SJ, Mels AK, Geijtenbeek TB, et al.. Preoperative granulocyte/macrophage colony-stimulating factor (GM-CSF) increases hepatic dendritic cell numbers and clustering with lymphocytes in colorectal cancer patients., *Immunobiology.* 2006;211:641-649.

75. Orozco H, Arch J, Medina-Franco H, et al.. Molgramostim (GM-CSF) associated with antibiotic treatment in nontraumatic abdominal sepsis: a randomized, double-blind, placebo-controlled clinical trial., *Arch Surg.* 2006;141:150-3; discussion 154.

76. Pettila V, Takkunen O, Varpula T, Markkola A, Porkka K, Valtonen V. Safety of granulocyte colony-stimulating factor (filgrastim) in intubated patients in the intensive care unit: interim analysis of a prospective, placebo-controlled, double-blind study., *Crit Care Med.* 2000;28:3620-3625.

77. Powell A, Creaney J, Broomfield S, Van Bruggen I, Robinson B. Recombinant GM-CSF plus autologous tumor cells as a vaccine for patients with mesothelioma., *Lung Cancer.* 2006;52:189-197.

78. Presneill JJ, Harris T, Stewart AG, Cade JF, Wilson JW. A randomized phase II trial of granulocyte-macrophage colony-stimulating factor therapy in severe sepsis with respiratory dysfunction., *Am J Respir Crit Care Med.* 2002;166:138-143.

79. Quittet P, Ceballos P, Lopez E, et al.. Low doses of GM-CSF (molgramostim) and G-CSF (filgrastim) after cyclophosphamide (4 g/m2) enhance the peripheral blood progenitor cell harvest: results of two randomized studies including 120 patients., *Bone Marrow Transplant.* 2006;38:275-284.

80. Schwaab T, Tretter CP, Gibson JJ, et al.. Tumor-related immunity in prostate cancer patients treated with human recombinant granulocyte monocyte-colony stimulating factor (GM-CSF)., *Prostate.* 2006;66:667-674.

81. Somani J, Lonial S, Rosenthal H, Resnick S, Kakhniashvili I, Waller EK. A randomized, placebo-controlled trial of subcutaneous administration of GM-CSF as a vaccine adjuvant: effect on cellular and humoral immune responses., *Vaccine.* 2002;21:221-230.

82. Vuylsteke RJ, Molenkamp BG, van Leeuwen PA, et al.. Tumor-specific CD8+ T cell reactivity in the sentinel lymph node of GM-CSF-treated stage I melanoma patients is associated with high myeloid dendritic cell content., *Clin Cancer Res.* 2006;12:2826-2833.

83. Weiss M, Gross-Weege W, Harms B, Schneider EM. Filgrastim (RHG-CSF) related modulation of the inflammatory response in patients at risk of sepsis or with sepsis., *Cytokine.* 1996;8:260-265.

84. Datenbank mit verdachtsfällen von impfkomplikationen und impfnebenwirkungen. *Paul-Ehrlich-Institut,* . from http://52625146fm.pei.de/fmi/iwp/cgi?-db=ADRDB&-loadframes

85. Neumann T, Spies C. Use of biomarkers for alcohol use disorders in clinical practice., *Addiction.* 2003;98 Suppl 2:81-91.

86. Rinck D, Frieling H, Freitag A, et al.. Combinations of carbohydrate-deficient transferrin, mean corpuscular erythrocyte volume, gamma-glutamyltransferase, homocysteine and folate increase the significance of biological markers in alcohol dependent patients., *Drug Alcohol Depend.* 2007;89:60-65.

87. DiMartini A, Day N, Lane T, Beisler AT, Dew MA, Anton R. Carbohydrate deficient transferrin in abstaining patients with end-stage liver disease., *Alcohol Clin Exp Res.* 2001;25:1729-1733.

88. Hartmann S, Aradottir S, Graf M, et al.. Phosphatidylethanol as a sensitive and specific biomarker: comparison with gamma-glutamyl transpeptidase, mean corpuscular volume and carbohydrate-deficient transferrin., *Addict Biol.* 2007;12:81-84.

89. Halter CC, Dresen S, Auwaerter V, Wurst FM, Weinmann W. Kinetics in serum and urinary excretion of ethyl sulfate and ethyl glucuronide after medium dose ethanol intake., *Int J Legal Med.* 2007.

90. Sorensen TI, Nielsen GG, Andersen PK, Teasdale TW. Genetic and environmental influences on premature death in adult adoptees., *N Engl J Med.* 1988;318:727-732.

91. Collins FS, Brooks LD, Chakravarti A. A DNA polymorphism discovery resource for research on human genetic variation., *Genome Res.* 1998;8:1229-1231.

92. Gordon D, Finch SJ, Nothnagel M, Ott J. Power and sample size calculations for case-control genetic association tests when errors are present: application to single nucleotide polymorphisms., *Hum Hered.* 2002;54:22-33.

17 Appendices

()

- Appendix 04: Richmond Agitation Sedation Scale (RASS)
- Appendix 05: Delirium Detection Scale (DDS)
- Appendix 06: Confusion Assessment Method for the Intensive Care Unit (CAM-ICU)
- Appendix 07: Nursing Delirium Screening Scale (Nu-DESC)
- Appendix 08: Intensivmedizinische Scoringsysteme
- Appendix 09: Fagerström
- Appendix 10: Alcohol Use Disorders Identification Test (AUDIT)
- Appendix 11: CAGE
- Appendix 12: Charité-Algorithmus zum Screening auf gefährlichen oder schädlichen Alkoholkonsum bzw. Abhängigkeit
- Appendix 13: Verantwortlichkeiten

Appendix 04

ChartéCentrum für Anästhesiologie, OP-Management und Intensivmedizin

Richmond Agitation Sedation Scale (RASS)

Punkte	Ausdruck	Beschreibung
+ 4	Streitlustig	Offene Streitlust, gewalttätig, unmittelbare Gefahr für das Personal
+ 3	Sehr agitiert	Zieht oder entfernt Schläuche oder Katheter, aggressiv
+ 2	Agitiert	Häufige ungezielte Bewegungen, atmet gegen das Beatmungsgerät
+ 1	Unruhig	Ängstlich aber Bewegungen nicht aggressiv oder lebhaft
0	Aufmerksam und ruhig	
- 1	Schläfrig	Nicht ganz aufmerksam aber erwacht anhaltend durch Stimme (> 10s)
- 2	Leichte Sedierung	Erwacht kurz mit Augenkontakt durch Stimme (< 10s)
- 3	Mäßige Sedierung	Bewegung oder Augenöffnen durch Stimme (aber keinen Augenkontakt)
- 4	Tiefe Sedierung	Keine Reaktion auf Stimme aber Bewegung oder Augenöffnen durch körperlichen Reiz
- 5	Nicht erweckbar	Keine Reaktion auf Stimme oder körperlichen Reiz

Appendix 05

Delirium Detection Score (DDS)

Item	Beschreibung	Punkte
Orientierung	Orientiert zu allen Qualitäten, Fähigkeit zur Konzentration<br>Nicht sicher orientiert zu Ort/Zeit, Unfähigkeit zur Konzentration<br>Nicht orientiert zu Ort und/oder Zeit<br>Nicht orientiert zu Ort, Zeit und Person	0<br>1<br>4<br>7
Halluzinationen	normale Aktivität<br>gelegentlich leichte Halluzinationen<br>permanent leichte Halluzinationen<br>permanent schwere Halluzinationen	0<br>1<br>4<br>7
Agitiertheit	normale Aktivität<br>leicht gesteigerte Aktivität<br>moderate Unruhe<br>schwere Unruhe	0<br>1<br>4<br>7
Angst	keine<br>gelegentlich leichte Angst<br>gelegentlich moderate Angst<br>Panikattacken	0<br>1<br>4<br>7
Schweißausbrüche	Keine<br>Meist unbemerkt, v. a. an den Händen<br>Schweißperlen auf der Stirn<br>Starkes Schwitzen	0<br>1<br>4<br>7

Appendix 06

Confusion Assessment Method for the Intensive Care Unit (CAM- ICU)

**1. akuter Beginn oder fluktuierender Verlauf**			**positiv bei einer „ja“ Antwort**	
**A**	hat sich der mentale Status verändert? (Baseline zuerst definieren)			
	☐ ja	☐ nein		
**Oder**				
**B**	Zeigten sich wechselnde mentale Zustände in den letzten 24h im Sinne eines veränderten RASS, GCS oder DDS?			
	☐ ja	☐ nein		
**2. Unaufmerksamkeit**			**positiv bei > 2 falschen Antworten:**	
**A**	Vorlesen einer Buchstabenreihe (S **A** V E **A** H **A** **A** R T), der Patient soll bei „**A**“ die Hand drücken (“Minuspunkt“ bei nicht oder falsch drücken)			
	☐ > 2 Minuspunkte	☐ ≤ 2 Minuspunkte		
**Oder**				
**B**	Zeigen von 5 Bildern (je 3 s), dann zeigen von diesen 5 und 5 anderen;der Patient soll die ersten Bilder wieder erkennen:			
	☐ > 2 falsche Antworten	☐ ≤ 2 falsche Antworten		
**3. Unorganisiertes Denken :**			**positiv bei ≥ 2 falschen Antworten**	
**A**	1. Schwimmt ein Stein auf Wasser?<br>2. Schwimmen Fische im See?<br>3. Wiegt 1 Kilo mehr als 2 Kilo?<br>4. Kann man mit einem Hammer einen Nagel einschlagen?			
	☐ >2 falsche Antworten	☐ ≤ 2 falsche Antworten		
**Oder**				
**B**	1. „Halten Sie **x** Finger hoch“<br>2. „Nun dasselbe mit der anderen Hand“ (Zahl nicht wiederholen)			
	☐ ≥ 2 falsch	☐ < 2 falsch		
**4. Veränderte Bewusstseinslage**			**positiv wenn RASS nicht = 0**	
	☐ RASS ungleich 0	☐RASS = 0		
**wenn 1+2 und entweder 3 oder 4 positiv ist, hat der Patient ein Delirium**				

Appendix 07

Nursing Delirium Screening Scale (Nu-DESC)

Nursing Delirium Screening Scale (Nu-DESC)
(Nu-DESC ≥ 2 → Delir)

**Symptom:**	Punkteverteilung 0 – 1 – 2 Punkte
**1. Desorientierung:**<br>(Desorientierung zu Zeit und oder Ort<br>falsche Einordnung der umgebenden Personen)	0 – 1 – 2
**2. Unangemessenes Verhalten:**<br>(z.B. Ziehen an Kathetern, unkooperatives Verhalten,<br>Selbstgefährdung durch Uneinsichtigkeit,<br>Missverstehen der Situation)	0 – 1 – 2
**3. Unangemessene Kommunikation:**<br>(Unpassendes Kommunikation zu Ort und Situation,<br>z.B. zusammenhanglose- oder gar keine<br>Kommunikation,<br>unverständliche sprachliche Äußerungen)	0 – 1 – 2
**4. Illusionen / Halluzinationen:**<br>(Sehen und oder Hören nicht vorhandener Dinge,<br>Verzerrung optischer Eindrücke)	0 – 1 – 2
**5. Psychomotorische Retardierung:**<br>(Verlangsamte Antworten / Reaktionen,<br>wenige oder keine spontane Äußerung / Aktivität<br>auch auf Stimulation<br>Bewusstseinsstörung<br>(RASS< -1))	0 – 1 – 2
**Summe**	

Appendix 08

Intensivmedizinische Scoringsysteme

APACHE II-Score (Acute Physiological And Chronic Health Evaluation)

Der APACHE-Score steht für akut physiologische und chronische Gesundheitsevaluation, also eine integrierte Betrachtung des akuten und chronischen Gesundheitszustands. Der APACHE-Score bemüht sich um eine Einstufung des Schweregrads der Erkrankungen im Hinblick auf die Sterbewahrscheinlichkeit (Mortalität) des Patienten.
Der APACHE-Score kann täglich erhoben werden und erfordert die Angabe der schlechtesten Werte der letzten 24 Stunden. Die physiologischen Parameter des Patienten werden je nach Abweichung von den Normalwerten gewichtet. Bei der täglichen Erhebung wird jedoch der Verlauf an den Vortagen nicht berücksichtigt.

SAPS II-Score (Simplified Acute Physiology Score)

Der SAPS II ist ein weit verbreitetes Instrument zur Schweregrad-Klassifikation von (erwachsenen) Intensivpatienten. Er wurde an einer großen multizentrischen und internationalen Datensammlung unter wesentlicher Beteiligung europäischer Intensivstationen entwickelt.

SOFA-Score (Sequential Organ Failure Assessment)

Der SOFA Score ist ein von Experten der European Society for Intensive Care Medicine (ESICM) festgelegter Konsens zur objektiven Beschreibung der Organ-(dys)-funktion - nicht nur bei Sepsis. Grundlage der täglichen Erhebung (24-Stunden-Zeiträume) sind die jeweils schlechtesten Werte für jedes Organsystem.

TISS 28-Score (Therapeutic Intervention Scoring System)

Der TISS 28-Score ist ein Instrument zur Erfassung und Dokumentation von diagnostischen, therapeutischen und pflegerischen Aktivitäten auf einer Intensivstation. Er

Appendix 09

erhebt täglich 28 Maßnahmen der Intensivtherapie, jede mit einer Punktzahl zwischen 1 und 4 bewertet. Der Summenwert aller durchgeführten Maßnahmen pro Tag wird dokumentiert.

Fagerström

**Frage**	**Wahlmöglichkeit**	**Bewertung**
Wann nach dem Aufstehen rauchen Sie Ihre erste Zigarette?	innerhalb von 5 min	3
	6 bis 30 min	2
	31 bis 60 min	1
	nach 60 min	0
Finden Sie es schwierig, an Orten, wo das Rauchen verboten ist (z.B. Kirche, Bücherei, Kino usw.) das Rauchen zu unterlassen?	ja	1
	nein	0
Auf welche Zigarette würden Sie nicht verzichten wollen?	die erste am Morgen	1
	andere	0
Wieviele Zigaretten rauchen Sie im allgemeinen pro Tag?	bis 10	0
	11 bis 20	1
	21 bis 30	2
	31 und mehr	3
Rauchen Sie am Morgen im allgemeinen mehr als am Rest des Tages?	ja	1
	nein	0
Kommt es vor, dass Sie rauchen, wenn Sie krank sind und tagsüber im Bett bleiben müssen?	ja	1
	nein	0
**Ihre Punkteanzahl**		

Appendix 10

Alcohol Use Disorders Identification Test (AUDIT)

0 P.<br>1 P<br>2 P.<br>3 P.<br>4 P.	**1. Wie oft nehmen Sie ein alkoholisches Getränk zu sich?**<br>☐Nie<br>☐1 x im Monat oder weniger<br>☐2 – 4 x im Monat<br>☐2 – 4 x in der Woche<br>☐4 x oder mehr die Woche	0 P.<br>1 P<br>2 P.<br>3 P.<br>4 P	**6. Wie oft brauchten Sie während der letzten 12 Monate am Morgen ein alkoholisches Getränk, um sich nach einem Abend mit viel Alkoholgenuss wieder fit zu fühlen?**<br>☐Nie<br>☐weniger als einmal im Monat<br>☐einmal im Monat<br>☐einmal in der Woche<br>☐täglich oder fast täglich
0 P.<br>1 P<br>2 P.<br>3 P.<br>4 P.	**2. Wenn Sie alkoholische Getränke zu sich nehmen, wie viel trinken Sie dann typischerweise an einem Tag?**<br>☐1 oder 2<br>☐3 oder 4<br>☐5 oder 6<br>☐7 - 9<br>☐10 oder mehr	0 P.<br>1 P<br>2 P.<br>3 P.<br>4 P	**7. Wie oft hatten Sie während der letzten 12 Monate wegen Ihrer Trinkgewohnheiten Schuldgefühle oder Gewissensbisse?**<br>☐Nie<br>☐weniger als einmal im Monat<br>☐einmal im Monat<br>☐einmal in der Woche<br>☐täglich oder fast täglich
0 P.<br>1 P<br>2 P.<br>3 P.<br>4 P.	**3. Wie oft trinken Sie 6 oder mehr Gläser Alkohol bei einer Gelegenheit?**<br>☐Nie<br>☐weniger als 1x im Monat<br>☐einmal im Monat<br>☐einmal in der Woche<br>☐täglich oder fast täglich	0 P.<br>1 P<br>2 P.<br>3 P.<br>4 P	**8. Wie oft haben Sie sich während der letzten 12 Monate nicht mehr an den vorangegangenen Abend erinnern können, weil Sie getrunken hatten?**<br>☐Nie<br>☐weniger als einmal im Monat<br>☐einmal im Monat<br>☐einmal in der Woche<br>☐täglich oder fast täglich

0 P.<br>1 P<br>2 P.<br>3 P.<br>4 P.	**4. Wie oft haben Sie in den letzten 12 Monaten erlebt, dass Sie nicht mehr mit dem Trinken aufhören konnten, nachdem Sie einmal begonnen hatten?**<br>☐ Nie<br>☐ weniger als 1x im Monat<br>☐ einmal im Monat<br>☐ einmal in der Woche<br>☐ täglich oder fast täglich ☐	0 P.<br>2 P.<br>4 P.	**9. Haben Sie sich oder eine andere Person unter Alkoholeinfluss schon einmal verletzt?**<br>☐ Nein<br>☐ Ja, aber nicht im letzten Jahr<br>☐ Ja, während des letzten Jahres<br>☐
0 P.<br>1 P<br>2 P.<br>3 P.<br>4 P.	**5. Wie oft passierte es in den letzten 12 Monaten, dass Sie wegen des Trinkens Erwartungen, die man an Sie in der Familie, im Freundeskreis und im Berufsleben hat, nicht mehr erfüllen konnten?**<br>☐ Nie<br>☐ weniger als 1x im Monat<br>☐ einmal im Monat<br>☐ einmal in der Woche<br>☐ täglich oder fast täglich ☐	0 P.<br>2 P.<br>4 P.	**10. Hat ein Verwandter, Freund oder auch ein Arzt schon einmal Bedenken wegen Ihres Trinkverhaltens geäußert oder vorgeschlagen, dass Sie Ihren Alkoholkonsum einschränken?**<br>☐ Nein<br>☐ Ja, aber nicht im letzten Jahr<br>☐ Ja, während des letzten Jahres<br>☐
			**Gesamtpunktzahl** ☐

Appendix 11

CAGE

Um schädlichen Alkoholkonsum handelt es sich nach CAGE, wenn von folgenden Fragen zwei oder mehr positiv beantwortet werden.

1. Haben Sie schon einmal das Gefühl gehabt, dass Sie weniger Alkohol trinken sollten?

2. Haben Sie sich schon einmal geärgert, dass andere Ihr Trinkverhalten kritisiert haben?

3. Haben Sie sich wegen Ihres Alkoholkonsums schon einmal schlecht oder schuldig gefühlt?

4. Haben Sie jemals bereits morgens Alkohol getrunken, um Ihre Nerven zu beruhigen oder einen Kater loszuwerden?

Appendix 12

Charité-Algorithmus zum Screening auf gefährlichen oder schädlichen Alkoholkonsums bzw. Abhängigkeit

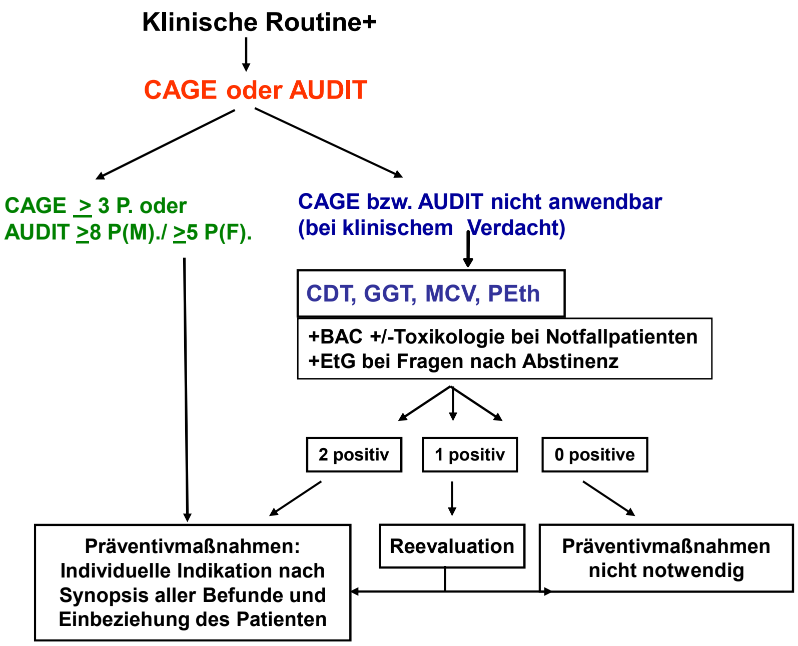

Appendix 13

Verantwortlichkeiten

Vertreter des Sponsor und Hauptprüfer der klinischen Prüfung	Prof. Dr. med. Claudia Spies<br>Universitätsklinik für Anästhesiologie mit Schwerpunkt operative Intensivmedizin<br>Charité – Universitätsmedizin Berlin,<br>Charité Campus Mitte<br>Charitéplatz 1, 10117 Berlin<br>Charité Campus Virchow-Klinikum<br>Augustenburger Platz 1, 13353 Berlin<br>Tel.: +49 (0) 30 450 55 11 02<br>Fax: +49 (0) 30 450 55 10 19<br>E-Mail: claudia.spies@charite.de
Anzahl der Prüfzentren	monozentrisch<br><br>Universitätsklinik für Anästhesiologie mit Schwerpunkt operative Intensivmedizin, Charité Campus Virchow- Klinikum und Campus Charité Mitte<br>Charité – Universitätsmedizin Berlin<br>Charité Campus Mitte<br>Charitéplatz 1, 10117 Berlin<br>Charité Campus Virchow-Klinikum<br>Augustenburger Platz 1, 13353 Berlin<br>Tel.: +49 (0) 30 450 55 11 02<br>Fax: +49 (0) 30 450 55 11 19<br><br>Der Patienteneinschluss findet ausschließlich am Campus Virchow-Klinikum statt.
Weitere Prüfärzte	Alawi Lütz, Alexander Schiemann, Lilit Sargsyan, Marco Paupers, Ulrike Wittkowski, Markus Renius<br><br>Universitätsklinik für Anästhesiologie mit Schwerpunkt operative Intensivmedizin<br>Charité – Universitätsmedizin Berlin,<br>Charité Campus Virchow Klinikum<br>Augustenburger Platz 1, 13353 Berlin<br>Charité Campus Mitte<br>Charitéplatz 1, 10117 Berlin<br>Tel.: 030-450 531 025<br>Fax: 030-450 551 019

Weitere beteiligte Personen und Institutionen, Prüflabore etc.	Prof. Dr. med. Peter Neuhaus<br>Universitätsklinik für Allgemein-, Visceral und Transplantationschirurgie<br>Charité Campus Virchow-Klinikum<br>Augustenburger Platz 1, 13353 Berlin<br>Tel.: + 49 30 450 55 20 01<br>Fax: +49 30 450 55 29 00
Monitoring ()	Kathrin Scholtz<br>Universitätsklinik für Anästhesiologie mit Schwerpunkt operative Intensivmedizin<br>Charité – Universitätsmedizin Berlin<br>Campus Virchow Klinikum<br>13353 Berlin<br>Tel.: +49 (0) 30 450 551 133<br>Fax: +40 (0) 30 450 551 909
**Biometrie und Datenmangement**	SOSTANA GmbH<br>Prof. Dr. Klaus-Dieter Wernecke<br>Wildensteiner Straße 27<br>10318 Berlin<br>Tel: +49 30 50158763<br>Fax: +49 30 50158906<br>E-Mail: kdwernecke@sostana.com
**Verantwortlicher der Klinikapotheke**	Dr. Regina Rybka-Golm<br>Klinikapotheke<br>Campus Virchow-Klinikum<br>Augustenburger Platz 1, 13353 Berlin<br>Tel.: +49 30 450 56 10 41<br>Fax: +49 30 450 56 19 04
**Referenzinstitutionen mit Nennung der Verantwortlichen (Labore)**	Zentralinstitut für Laboratoriumsmedizin und Pathobiochemie, Campus Virchow-Klinikum, Charité – Universitätsmedizin Berlin<br>Direktor: Prof. Dr. med. R. Tauber<br>Augustenburger Platz 1<br>13353 Berlin<br>Tel.: 030 450 569 001<br>Fax: 030 450 569 900<br><br>Institut für Medizinische Immunologie, Campus Charité Mitte, Charité – Universitätsmedizin Berlin<br>Direktor: Prof. Dr. med. H.-D. Volk<br>Augustenburger Platz 1<br>13353 Berlin<br><br>Tel: 030 450 524 062<br>Fax:030 450 524 932

	Universitätsklinik für Anästhesiologie mit Schwerpunkt operative Intensivmedizin<br>Direktorin: Prof. Dr. med. Claudia Spies<br>Charité – Universitätsmedizin Berlin,<br>Charité Campus Mitte<br>Charitéplatz 1, 10117 Berlin<br>Charité Campus Virchow-Klinikum<br>Augustenburger Platz 1, 13353 Berlin<br>Tel.: 030 450 531 012 / 52<br>Fax: 030 450 531 911
