# Supplementary material for: Influence of Granulocyte-Macrophage Colony-Stimulating Factor or Influenza Vaccination on HLA-DR, Infection and Delirium Days in Immunosuppressed Surgical Patients: Double Blind, Randomised Controlled Trial
Source: PLoS One. 2015 Dec 7;10(12):e0144003. doi: 10.1371/journal.pone.0144003 (PMC4671639; doi:10.1371/journal.pone.0144003)
Supplement: S2 Protocol — (DOC) [file pone.0144003.s004.doc]

Study synopsis

| **Study title** | A study comparing granulocyte macrophage-colony stimulating factor (GM-CSF), vaccination and placebo in patients who suffer from immune depression after resection of either pancreas or oesophagus  Code: ART VI |
| --- | --- |
| **Kind of project** | *Clinical Trial according to German Drug Law (AMG) Phase III*  *EudraCT-Nr.: 2007-003111-31* |
| **Sponsor** | Charité, Universitätsmedizin – Berlin  Charitéplatz 1  10117 Berlin  Tel.: 030-450 570 142  Fax: 030-450 570 914 |
| **Principal investigator** | Prof. Dr. Claudia Spies  Klinik für Anästhesiologie m.S. operative Intensivmedizin  Augustenburger Platz 1, 13353 Berlin  Tel. +49 450 551102  Fax +49 450 551909  claudia.spies@charite.de |
| **Study center** | Single center:  Klinik für Anästhesiologie m.S. operative Intensivmedizin  Campus Charité Mitte and Campus Virchow Klinikum,  Charité - Universitätsmedizin Berlin |
| **Duration of trial** | Start of the study: 26/10/2008  Expected end of the study: 30/09/2011 (insurance confirmation) |
| **Aims** | The aim of the present study is to investigate the effects of a postoperative vaccination or application of GM-CSF in patients undergoing elective esophageal or pancreatic surgery on immune response and postoperative infection rate.  Primary outcome parameters  HLA-DR Expression on Monocytes  Secondary outcome parameters  Post-operative infection  Post-operative delirium  Hospital Stay  ICU Stay  Time on Ventilator (TOV)  ICU-scores APACHE II score, SAPS II score, SOFA score, TISS-28 score  Immune parameters (for the first 33 study patients) |
| **Study design** | Prospective, randomised, double-blinded, double-dummy, placebo-controlled, monocentre pilot study.  Patients with planned oesophageal or pancreatic surgery are screened and evaluated.  If HLA-DR is ≤10.000 antibodies per cell in the morning of postoperative day one patients are randomly and blinded assigned to placebo or GM-CSF (Sargramostim, Leukine©) or vaccination with Mutagrip© group. |
| **Study medication: intervention medication/**  **compare intervention medication** | On day one after surgery (POD 1) the included patients are randomised and receive blinded perfusion over 24 hours with a perfusion rate of 1ml/h and one subcutaneous injection. The groups with verum receive either vaccine (Mutagrip©) subcutaneous or intravenous GM-CSF (Leukine©). The substances for subcutaneous injection consist of placebo (0.9% sodium chloride) or verum (vaccination; Mutagrip©). The substances for perfusion over 24 hours consist of placebo (0.9% sodium chloride) or verum (GM-CSF; Leukine©). In the placebo group 0.9% sodium chloride is given as subcutaneous injection and 24 hours perfusion. Sodium chloride 0.9% (Braun©) 0.5 ml is subcutaneously injected and/or 24ml of sodium chloride 0.9% (Braun©) are intravenously administered.  On the second and third day after surgery the administration of the substances is continued for 24h when HLA-DR is ≤10.000 antibodies per cell in blood sample. |
| **Safety** | During the trial adverse events (AE) and severe adverse events (SAE) have to be recorded. If an adverse events has not been resolved at discharge examination (9. postoperative day) it has to be followed up for further 7 days; not resolved serious adverse events at discharge examination (9. postoperative day) are followed up until the 21. postoperative day. |
| **Number of patients** | Planned number of patients is 60 (20 patients in each group: vaccination group, GM-CSF and placebo group). |
| **Inclusion criteria** | Inclusion criteria:  Patients were included if   - elective oesophageal or pancreatic surgery - ≥18 years old, - informed consent, written explanation, - no pregnancy, effective contraception or postmenopausal period over two years - no participation in another study concerning German Drug Law (AMG) one month before and during the study - HLA-DR expression on monocytes ≤ 10.000 antibodies per cell on day one after surgery. |
| **Exclusion criteria** | Exclusion criteria:   - age < 18 years - pregnant or lactating - infection with HIV or hepatitis B and C - consent was declined - storage of patient’s anonymized data was declined - participation in another study concerning German Drug Law (AMG) - employee of Charité - acquired or congenital blood cell disease, leukaemia - allergy regarding Mutagrip© and Leukine© and their ingredients - chemotherapy or radiotherapy within the last 28 days - proven infection within the last 7 days - autoimmune diseases - pharmacological immunosuppression within the last four weeks - instable cardiac disease with angina pectoris - untreated arrhythmia - symptomatic cardiac anomalies - thromboembolic events - bodyweight <50kg - monocyte HLA-DR >10.000 monoclonal antibodies per cell on day 1 after surgery - thrombocytes ≤ 100.000/μl - neutrophiles ≤ 1.500/μl - haemoglobin ≤ 8g/dl - bilirubin > 2g/dl - creatinine > 1,5g/dl - Aspartate aminotransferase (AST)/ alanine aminotransferase (ALT) > 90 U/l |
| **Statistical methods** | For all targets, the results are only exploratory investigated and analyzed descriptively, i.e. statistical measures such as mean and variance (Metric traits), median and interquartile difference  (Ordinal characteristics) and reached frequencies are determined by nominal characteristics. The obtained results are  used to plan a subsequent prospective controlled clinical trial with appropriate statistical planning of sample size. Performed statistical tests are solely exploratory nature and can lead to interesting hints for scientific questioning of the study, but should not be interpreted confirmatory or generalized. For the same reason no adjustment of first type error was adjusted if tested several times. The evaluations each should be performed stratified for possible influencing factors. The evaluation of the study results follows both the "intent-to-treat" – as well as the "per-protocol" -principle.  Statistical software is performed by the SAS, Version 8.2, SPSS, version 13, S-PLUS 2000 and StatXact 5 of CYTEL  The fixed sample size of 20 patients per group is based solely on the circumstances of the Clinic and the structural properties of the primary target and was not statistically planned. |

|  | Run-in | Screening | Screening | 1. Visit | 2. Visit | 3. Visit | 4. Visit | 5. Visit | 6. Visit | 7. Visit | 8. Visit | 9. Visit | **Follow-up** | **Follow-up** |
| --- | --- | --- | --- | --- | --- | --- | --- | --- | --- | --- | --- | --- | --- | --- |
|  | vOP | OP | 1. pOP | 1. pOP | 2. pOP | 3. pOP | 4. pOP | 5. pOP | 6. pOP | 7. pOP | 8. pOP | 9. pOP | **16. pOP** | **30. pOP** |
| Patient information | ✓ | - |  | - | - | - | - | - | - | - | - | - | - | - |
| Informed consent | ✓ | - |  | - | - | - | - | - | - | - | - | - | - | - |
| Patients history | ✓ | - |  | - | - | - | - | - | - | - | - | - | - | - |
| Check of in- and exclusion criteria | ✓ | ✓ |  | - | - | - | - | - | - | - | - | - | - | - |
| Clinical examination/diagnosis | ✓ | ✓ |  | - | - | - | - | - | - | - | - | - | - | - |
| Pregnancy test* | ✓ | - |  | - | - | - | - | - | - | - | - | - | - | - |
| Blood of the target parameters | - | **✓** | **✓** | - | **✓** | **✓** | **✓** | **✓** | - | - | - | - | - | - |
| Randomization | - | - | - | **✓** | - | - | - | - | - | - | - | - | - | - |
| Application of study medication | - | - |  | **✓** | **✓** | **✓** | - | - | - | - | - | - | - | - |
| Check of concomitant medication | ✓ | ✓ |  | ✓ | ✓ | ✓ | ✓ | ✓ | ✓ | ✓ | ✓ | ✓ | - | - |
| Evaluation of intensive medical security parameters** | - | ✓ |  | ✓ | ✓ | ✓ | ✓ | ✓ | ✓ | ✓ | ✓ | ✓ | - | - |
| Documentation of AEs, SAEs und SUSARs | ✓ | ✓ |  | ✓ | ✓ | ✓ | ✓ | ✓ | ✓ | ✓ | ✓ | ✓ | - | - |
| Documentation of AE’s^#4^ | - | - |  | - | - | - | - | - | - | - | - | - | ✓ | - |
| Documentation of SAE’s^#5^ | - | - |  | - | - | - | - | - | - | - | - | - | ✓ | ✓ |
| Final examination^#6^ | - | - |  | - | - | - | - | - | - | - | - | ✓ | - | - |

*women of childbearing potential; childbearing potential defined as: up to 2 years after menopause

**previous completion of documentation of the intensive care parameters if shifted to peripheral ward: RR, HF, ZVP, arterial pressure, temperature, APACHE-II-Score, TISS-28-Score, SAPS-II, SOFA-Score

^#4^Subject to unfinished AEs at study end (postoperative day 9)

^#5^Subject to unfinished SAEs at study end (postoperative day 9)

^#6^In case of untimely study termination the final examination must be carried out at the time of study termination
