# Supplementary material for: Influence of Granulocyte-Macrophage Colony-Stimulating Factor or Influenza Vaccination on HLA-DR, Infection and Delirium Days in Immunosuppressed Surgical Patients: Double Blind, Randomised Controlled Trial
Source: PLoS One. 2015 Dec 7;10(12):e0144003. doi: 10.1371/journal.pone.0144003 (PMC4671639; doi:10.1371/journal.pone.0144003)
Supplement: S1 Diagram — (DOC) [file pone.0144003.s006.doc]

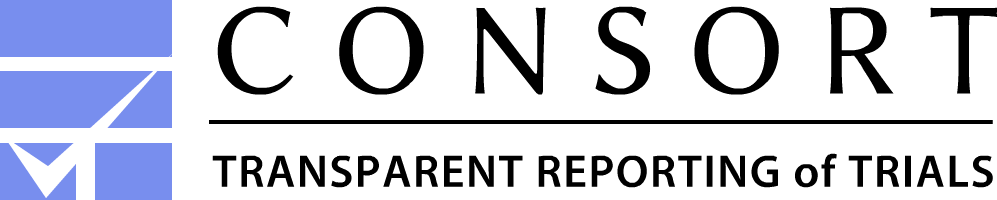
**CONSORT 2010 Flow Diagram**

**Allocation**

**Analysis**

**Follow-Up**

**Enrollment**

Assessed for eligibility (n=319)

Excluded (n=235)

♦  Not meeting inclusion criteria (n=199)

♦  Declined to participate (n=36)

Written informed consent (n=84)

♦  excluded due HLA-DR > 10,000 on pod1 (n=18)

♦ excluded due to other reasons (n=3)

)

Analysed (n=20)
♦ Excluded from analysis (give reasons) (n=1, see above)

Lost to follow-up (give reasons) (n=1, died of multiorgan failure)
Discontinued intervention (give reasons) (n=1, see above)

Allocated to intervention **GM-CSF** (n=21)

♦ Received allocated intervention (n=21)

♦ Did not receive allocated intervention (give reasons) (n=0)

Lost to follow-up (give reasons) (n=0)

Discontinued intervention (give reasons) (n=0)

Allocated to intervention **Vaccination** (n=21)

♦ Received allocated intervention (n=21)

♦ Did not receive allocated intervention (give reasons) (n=0)

Analysed (n=21)
♦ Excluded from analysis (give reasons) (n=0)

Randomized (n=63)

Lost to follow-up (give reasons) (n=0)

Discontinued intervention (give reasons) (n=0)

Allocated to intervention **Placebo** (n=21)

♦ Received allocated intervention (n=21)

♦ Did not receive allocated intervention (give reasons) (n=0)

Analysed (n=20)
♦ Excluded from analysis (give reasons) (n=1, inadvertently unblinded)
